# The influence of type 2 diabetes and its metabolic correlates in middle-aged adults on cognition at mid and later life; A systematic review and meta-analysis

**DOI:** 10.1371/journal.pone.0327408

**Published:** 2025-12-05

**Authors:** Oisin C. Joyce, Cliodhna McHugh, David Mocker, Fiona Wilson, Áine M. Kelly

**Affiliations:** 1 Department of Physiology, School of Medicine, Level 2, Trinity Biomedical Sciences Institute, Trinity College Dublin, Dublin, Ireland; 2 John Stearne Library, Trinity Centre, St James’s Hospital, Dublin, Ireland; 3 Discipline of Physiotherapy, School of Medicine, Trinity Centre for Health Sciences, St James’s Hospital, Dublin, Ireland; Australian National University, AUSTRALIA

## Abstract

**Introduction:**

While previous studies have examined the link between Type 2 Diabetes Mellitus (T2DM) and cognitive function in middle-aged adults, no review has explored the long-term effects on cognition of T2DM when diagnosed at midlife. This review aims to investigate any association between T2DM and its metabolic correlates during midlife and measures of cognitive function, spanning from midlife into later life.

**Methods:**

Electronic databases (EMBASE, Medline, CINAHL, Web of Science) were explored from their establishment until December 2023 to identify studies exploring the link between T2DM in midlife (40–65 years) and cognitive function. T2DM was defined based on ESC and AHA criteria, encompassing diabetes status, FBG, HbA1c levels, and MetS presence. Cognitive function in mid and/or later life was categorised into functioning sub-domains, with tests selected to reflect predominant cognitive functions utilised. A random-effects meta-analysis was performed, and study quality assessed using the AXIS tool.

**Results:**

We included 151 studies of moderate to high quality. Studies were independently screened in a step-by-step process, with a subset of studies that met the criteria selected for inclusion in a meta-analysis. Due to limited availability of raw data for cognitive measures at later life, meta-analysis was conducted on studies assessing cognitive function at midlife only. In the case of later life, the majority of longitudinal studies reported negative relationships between midlife T2DM and cognitive function, specifically in the modalities of executive function and global cognition; findings for memory were conflicting. Qualitive assessment of 107 studies where cognitive function was assessed at midlife found no association between it and midlife T2DM. However, meta-analysis of 10 studies revealed a negative impact of T2DM on memory (MD = −0.19; 95% CI = −0.26 to −0.11; I2 = 25%), executive function (MD = −0.14; 95% CI = −0.25 to −0.04; I2 = 0%), and global cognition (MD = −0.26; 95% CI = −0.34 to −0.17; I2 = 0%).

**Discussion:**

This study highlights the impact of midlife T2DM on a variety of cognitive domains from midlife onwards, suggesting that timely diagnosis of T2DM and its careful management may be an important strategy in preserving cognitive function through the lifespan. Given the contrast in results from qualitative and quantitative analysis that we report in the case of midlife cognitive function, we also emphasise the value of combining both methodological approaches where possible.

## 1. Introduction

Despite being previously confined to older populations, there is a growing prevalence of Type 2 diabetes mellitus (T2DM) among adults, including those in midlife [1], with the number of people diagnosed globally expected to exceed 700 million by 2045 [2]. Exposure to T2DM during midlife has been shown to accelerate neurodegeneration and structural neuropathology [3], while high levels of blood glucose with advancing age can impact the circulatory, renal and peripheral nervous systems [4]. However, there remains a lack of comprehensive synthesis regarding the impact of T2DM on cognitive decline, despite evidence suggesting that individuals with diabetes are 1.5–2.0 times more likely to develop cognitive deterioration, ranging from subtle to severe, and even leading to dementia in some instances [5]. With 20% of adults aged 65–69 years estimated to have T2DM, understanding the relationship between T2DM and cognitive decline, particularly during midlife, is crucial for effective management and implementation of intervention strategies with the potential to slow progression of cognitive ageing and development of dementia in this population [6].

The physiological mechanisms underlying neurodegeneration and associated cognitive decline in the presence of T2DM are not fully understood but are considered multifactorial [7]. Altered glucose metabolism has been linked to minor reductions in cognition with more severe impairment found in those with prolonged T2DM [8,9]. Studies suggest that sustained hyperglycaemia can lead to macro- and micro- vasculature injury, specifically endothelial dysfunction, resulting in cerebral vasculature injury [10] and impairment of neuronal function [11]. Neuroimaging studies report that cognitive impairment in diabetic patients mirrors the pathology of vascular dementia and Alzheimer’s Disease, through lower cerebral blood flow [12], reduced total brain volume [13], and structural alterations to the hippocampus and basal ganglia [14]. The contribution of this cerebral regional atrophy is linked with reduced executive function and memory [15]. Additionally, T2DM increases oxidative stress and endothelial dysfunction, which are associated with reduced psychomotor speed, mental flexibility, and attention [16]. Therefore, T2DM has the potential to impact cognitive function through multiple mechanisms, likely beginning at the onset of T2DM and progressing through the lifespan, precipitating diminished cognitive reserve and/or resilience [13].

Greater understanding of the relationship between T2DM and cognition during ageing, especially from early middle-age onwards, could inform appropriate age-dependent and time-sensitive management of T2DM [17]. A negative relationship between T2DM and cognitive function at middle age has been previously reported [18,19]. However, no review has evaluated the impact of T2DM at midlife on different aspects of cognitive function across the lifespan, to include and thus allow comparison of both lifestages of midlife and later life. Therefore, the aim of this review was to systematically assess the impact of midlife T2DM on cognitive function, measured across different domains, at midlife and later life. The secondary aim was to determine whether there is a relationship between metabolic correlates of T2DM (fasting blood glucose [FBG], glycosylated haemoglobin [HbA1c] and metabolic syndrome [MetS]) and cognitive function.

## 2. Methods

This systematic review was undertaken in accordance with the PRISMA (Preferred Reporting Items for Systematic Reviews and Meta-Analyses) statement (PRISMA; www.prisma-statement.org) and registered with PROSPERO (CRD42021238293; https://www.crd.york.ac.uk/prospero/). The presented review is a sub-analysis of a larger registered review of midlife cardiovascular health and later life cognition [20].

### 2.1 Search strategy

Electronic databases, including MEDLINE, EMBASE, Web of Science, and CINAHL were searched from their inception to December 2024; searches were not restricted for language or publication date. The comprehensive search strategy and screening protocols employed have been detailed extensively in our previously published paper, [20]. Briefly, the search strategy comprised of key words, MeSH terms, common medical terms, and a combination of these including, but not limited to, middle age, midlife, cardiovascular disease, cardiovascular risk, diabetes mellitus, fasting blood glucose, cognition, and cognitive defect. The search strategy focused on the inclusion of longitudinal, prospective, and follow-up studies to ensure later life cognition was captured It is important to highlight that the current search strategy is specifically tailored to focus on T2DM. A manual search of the reference lists of included studies was undertaken to identify any additional articles for inclusion. The full search strategy can be found as supplementary material (see S1 File).

**Fig 1:**
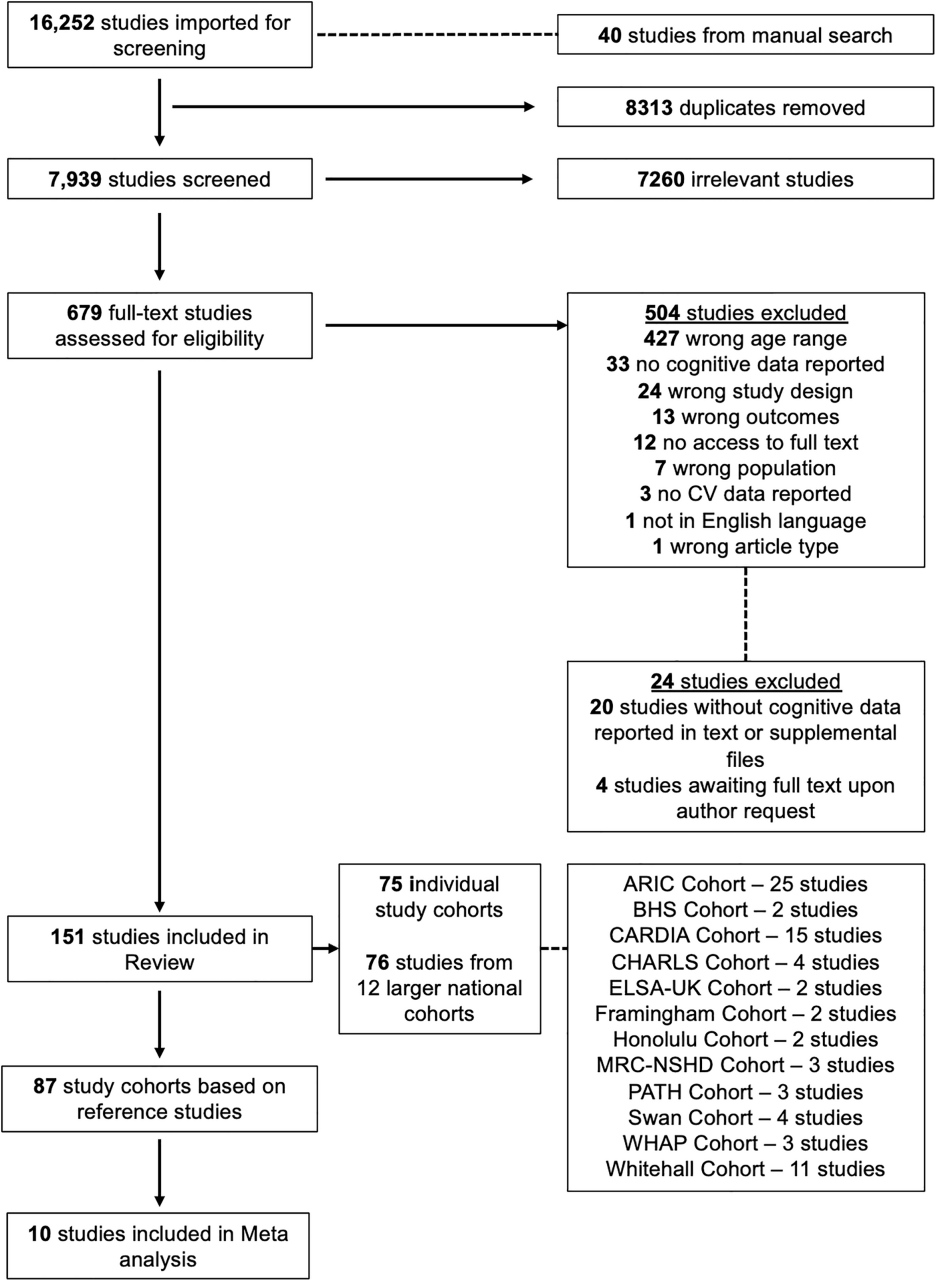
Flow chart of the study selection process.

The step-by-step search process can be seen in Fig 1. Two authors (O.C.J. and C.McH.) independently screened titles and abstracts and subsequent full texts using Covidence (https://www.covidence.org/home). Disagreements between authors were resolved by consensus, and if a compromise was not agreed, a third author (F.W or A.K) was consulted.

### 2.2 Eligibility criteria

Studies were deemed eligible for inclusion based on the following criteria: human participants, middle aged adults between ages of 40–65 years, at least one outcome measure of diabetes according to the European Society of Cardiology (ESC [21]) and American Diabetes Association (ADA [22]), including diabetes status (Yes or No) and metabolic metrics; FBG, HbA1c levels, and the presence or absence of MetS reported at later life, midlife, or both. Cognitive domains included memory, attention, executive function, intelligence, and global cognitive functioning (see S1 File). Studies were excluded if cognitive testing was undertaken by a proxy or designated respondent, such as a friend or family member, if the participant cohorts included those with midlife dementia or any form of pre-existing cognitive impairment and if the study design was review based (systematic, meta-analysis or narrative), trial registrations, letters and editorials, and regulatory information.

### 2.3 Data extraction

Relevant data were extracted in accordance with STROBE guidelines [23] including study aims, participant characteristics, measures of cognition and cardiovascular risk factors alongside relevant outcome data as group means, standard deviation (SD), standard error (SE) of the mean, statistical significance, and precision estimates. Studies were assigned a reference number for data extraction. Pro-forma data extraction was piloted against a selection of papers prior to commencing data extraction. Where multiple studies from prospective longitudinal cohorts were included, the most recent publication was chosen as the reference study. Endnote version 20 was then used to create a database.

### 2.4 Risk of bias and methodological assessment

Methodological quality was determined using the AXIS tool (Critical appraisal tool to assess the quality of cross-sectional studies) [24] independently by two authors (O.C.J. and C.McH.). This appraisal tool used a series of 20 questions to determine study quality and risk of bias by means of answering ‘Yes’, ‘No’ or ‘Unsure’ as seen in previous reviews [25]. Answers were colour coded highlighting the impact of response towards overall bias and study quality: green, positive impact on quality of study; red, negative impact on quality of study; and amber, unknown impact on quality of study. Disagreements between authors were resolved by consensus, and if a compromise was not agreed, a third author (F.W or A.K) was consulted. Study quality was classified as low, moderate, or high determined by the number of positive, negative and unknown impacts on study quality identified.

### 2.5 Statistical analysis

The weighted mean of all cognitive measures, FBG, and HbA1c values were calculated whereby each value was assigned a weight corresponding to its r frequency; i.e., number of participants. These weights were then multiplied by their respective values and summed, with the total weighted sum divided by the sum of the weights to obtain the weighted mean, providing a measure of central tendency that accounts for the varying importance of individual data points [26]. This was undertaken using the following formula:


$$\text{Weighted Average}=\frac{\sum_{i=\text{1}}^{n}X_{i}w_{i}}{\sum_{i=\text{1}}^{n}w_{i}}$$

where *x_i_* represents each individual value in the dataset; *w_i_* represents the weight corresponding to each value *x_i_*; and *n* is the total number of values in the dataset. Qualitative analysis was used to determine associations between T2DM and its correlates (FBG, HbA1c and MetS) and cognition (positive, negative, or neutral) at both midlife and later life across all studies. A qualitative analysis approach was employed to extract and synthesise findings from all included studies. Specifically, we identified and documented relationships as reported by the authors, considering them as study results for inclusion in our review findings. This method allowed us to capture and present the nuanced interpretations and insights within the context of their respective studies and in line with our aim(s) and outcome measures. Cognitive outcome measures were grouped according to cognitive domain and further sub-divided where appropriate based on included cognitive tests.

A random effects meta-analysis was undertaken among select studies to compare differences across each cognitive domain between two independent groups: presence of T2DM vs. absence of T2DM. This meta-analysis was deemed appropriate to calculate the pooled summary effect of midlife T2DM diagnosis on cognition at midlife. Group mean differences, 95% confidence intervals (CIs) and P-values were calculated using Review Manager (RevMan) software ([Computer programme], Version 5.4, Copenhagen: The Nordic Cochrane Centre, The Cochrane Collaboration, 2020). Sub-grouping for meta-analyses included study design and quality. The heterogeneity between studies was established using the I^2^ statistic. I^2^ values of 25%, 50%, and 75% (p > 0.05) correspond to low, moderate, and high degrees of heterogeneity respectively [27]. Where high levels of heterogeneity (I^2^ > 75%) were detected and a sufficient availability of studies was present, sensitivity analysis was conducted for each cognitive domain by systematically removing one study at a time to assess its impact on the variability of the results. This helped to ensure the robustness of our findings to various decisions made during the review process, such as eligibility criteria and data completeness to identify potential influential factors and address uncertainties, ensuring the reliability and validity of our conclusions. By ensuring findings were not overly dependent on arbitrary decisions, sensitivity analysis helped maintain the reliability of our meta-analysis. Studies removed due to the sensitivity analysis are represented by a 0.0% weight in the forest plots.

Furthermore, in conducting our meta-analysis we employed a methodological approach to avoid double counting of participants and potential overestimation of results. While each study may have assessed multiple cognitive tests within a specific cognitive domain, we included only one primary cognitive test result per domain (most common cognitive test reported across studies included) to prevent redundancy and maintain the independence of comparisons, adhering to recommended strategies outlined in the literature [28]. This approach ensured that each study contributed one independent comparison, addressing concerns regarding unit-of-analysis errors, and enhancing the credibility of our findings. By providing this clarification, we aimed to assure transparency and rigor of our approach, thereby maintaining the integrity and reliability of our meta-analysis results in assessing the influence of T2DM on cognition in middle-aged adults.

## 3. Results

### 3.1 Literature search

Fig 1 provides full detail of study selection. The initial search returned 16,252 records. Following the removal of duplicates and screening of titles and abstracts, 679 studies remained with 504 subsequently excluded during full text screening. A further 24 studies were excluded owing to no response from authors contacted for access to full text or raw cognitive data. Included studies were imported into a database using Endnote version 20 for data extraction in Microsoft Excel. In total, 151 studies published between 1995 and 2024 were included resulting in 87 reference studies.

### 3.2 Methodological and risk of bias assessment

Overall, studies included were deemed to be of moderate- to- high quality; 55 high, 64 moderate and 33 low quality studies (see S1 File). Studies shared common weaknesses, including failing to justify sample size (n = 139), not addressing non-responders (n = 134), not providing information about non-responders (n = 139), no clear assessment of statistical significance (n = 51), and/or failing to state whether ethical approval was granted (n = 17).

### 3.3 Study characteristics

Of all included studies, 130 studies assessed both males and females, while 12 studies assessed males only and 9 assessed females only. 51 studies examined data from 12 prospective longitudinal cohorts, with the largest numbers derived from the Atherosclerosis Risk in Communities (ARIC) cohort (n = 25), Coronary Artery Risk Development in Young Adults (CARDIA) cohort (n = 15), and Whitehall cohort (n = 11) (see S1 File). The remaining studies examined data from independent study cohorts. Studies were undertaken across 28 nations, including United States (n = 59), the United Kingdom (n = 20), China (n = 9), Australia (n = 6), and India (n = 8).

### 3.4 Participant characteristics

A total of 125,711 participants were pooled for analysis. The weighted mean age of participants was 52.6 ± 3.8 years. The weighted mean BMI was 27.3 ± 4.9 kg/m2. The mean height and weight were 165.8 ± 1.5 cm and 76.4 ± 13.1 kg, respectively. Of studies that provided data by sex (n = 61), 48,428 males and 54,444 females were included. Weighted mean BMI was 25.3 ± 3.2 kg/m^2^ and 26.4 ± 4.7 kg/m^2^ for males and females, respectively. Males and females were of similar age (53.5 ± 3.7 vs. 53.5 ± 3.2 years) and females were shorter than males (174 ± 7.6 vs. 161.2 ± 5.9 cm. The average weight by sex was unavailable.

### 3.5 Measures of T2DM and its metabolic correlates

Tables 1 and 2 details all measures of T2DM and MetS across studies. Mean FBG across the total participants was 93.8 ± 20 mg/dl and 4.8 ± 0.76 for HbA1c, assessed across 49 and 21 studies, respectively. Of studies that assessed diabetes status) 16,120 were diabetic and 112,270 were classified as non- diabetic. Of studies that assessed MetS (n = 7), 1,729 participants were classified as having MetS, whereas 10,474 did not.

**Table 1:** Summary Table of weighted average for all cognitive measures and associated diabetes metrics at baseline (i.e., midlife).

Cognitive Variable	No. of Studies	Weighted Average	Age (Years)	FBG (mg/dl)	HbA_1_c (mg/dl)
Memory Verbal Memory	**Total:** n = 18 **Immediate**: n = 5, **Delayed**: n = 5, **STW**: n = 1, **EBM**: n = 2, **RAVLT (Immediate &** **Delayed recall):** n = 3, **RAVLT (Learning Score):** n = 1, **RAVLT (Summary Metric):** n = 3 **SRT:** n = 1, **ROCF (Immediate &** **Delayed recall):** n = 1, **CERAD (Immediate & Delayed):** n = 1, **CVLT (Immediate & Delayed):** n = 1 **RCF (Delayed recall): **n = 1	**Immediate:** Total = 9.9, Males = 15.8 ± 4, Females = 17.6 ± 4.1 **Delayed:** Total = 8.6 ± 1.4, Males = 4.1 ± 1.6, Females = 4.2 ± 1.7 **STW**: Total = 50.5 **EBM**: Total = 10.2 ± 1.1 **RAVLT (Immediate & Delayed recall):** Total = 7.1 ± 2.7, 8.7 ± 3.4 **RAVLT (Learning Score):** Total = 36.8 ± 8.3 **RAVLT (Summary metric):** Total = 8.9 ± 3.2 **SRT:** Total = 34.3 **ROCF (Immediate &** **Delayed recall): **16.1** **± 7.6, 14.9 ± 7.8 **CERAD (Immediate & Delayed):** Total = 7.2 ± 1.1, 7.7 ± 1.5 **CVLT (Immediate & Delayed):** Total = 8.8 ± 2.1, 8.8 ± 3.2 **RCF (Delayed recall): **Total = 21.1 ± 7.6	**Total **= 50.9 ± 4.6, Males = 56.6 ± 7.1, Females = 56.2 ± 7.1	**Total **= 90.9 ± 19.3 **Delayed:** Not available **Immediate:** Not available **EBM:** Not available **RAVLT (Summary metric):** Total = 100.9 ± 17.2 **RAVLT (Immediate & Delayed recall, Learning Score):** Total = 101.5 ± 12.5, 131.9 ± 48.8 **SRT:** Total = 86.2 ± 20.9 **ROCF (Immediate &** **Delayed recall): **Total = 131.9 ± 48.8 **CERAD (Immediate & Delayed):** Total = 95.22 ± 21.96 **CVLT (Immediate & Delayed):** Total = 95.22 ± 21.96 **RCF (Delayed recall): **Total = 91	Total = 5.7 ± 0.4 Delayed: Not available Immediate: Not available EBM: Not available RAVLT (Summary metric): Not available RAVLT (Immediate & Delayed recall, Learning Score): Total = 7.0 ± 1.4, 5.7 ± 0.4 SRT: Not available ROCF (Immediate & Delayed recall): Total = 7.0 ± 1.4 CERAD (Immediate & Delayed): Not available CVLT (Immediate & Delayed): Not available RCF (Delayed recall): Total = 5.2
Episodic Memory	**Total:** n = 4	**Total** = 6.7 ± 2.1	**Total **= 56.9 ± 5.9, Males = 50.3 ± 8, Females = 51 ± 8.1	**Total** = 106.5 ± 19.8, Males = 95.4 ± 10.08, Females = 93.6 ± 9.72	Total = 5.3 ± 0.6
Semantic Memory	**Total:** n = 2	**Total** = 19.4 ± 0.3 Males = 15.2 ± 3.0, Females = 15.9 ± 2.8	**Total **= 50.7 ± 8, Males = 50.3 ± 8, Females = 51 ± 8.1	**Total **= 95.4 ± 9.9, Males = 95.4 ± 10.08, Females = 93.6 ± 9.72	Not available
Working Memory	**Total**: n = 23 **DSST:** n = 9 **CMS Score:** Total: n = 1 **DSB Test**: n = 8 **MIS (MoCA):** n = 1 **VRT**: n = 1 **LMT:** n = 1 **Composite:** n = 1 **WAIS:** n = 1	**DSST:** Total = 47.8 ± 11.7 **CMS Score:** Total = 76.6 ± 12.9 **DSB Test:** Total = 6.2 ± 1.9 **MIS (MoCA):** Total = 12.7 ± 2.4 **VRT:** Total = 11.3 **LMT:** Total = 17.2 ± 6.9 **Composite:** 12.88 ± 0.6 **WAIS:** Males = 38.6 ± 3.9, Females = 38.9 ± 4.7	**Total **= 52.1 ± 4.6	**Total **= 98.7 ± 24.9 **DSST:** Total = 108 ± 27 **CMS Score:** 89.54 ± 9.5 **DSB Test:** Total = 104.9 ± 19.3 **MIS (MoCA):** Not available **VRT:** Total = 86.2 ± 21.4 **LMT:** Total = 95.1 ± 11.7 **Composite: 176.8 ± 5.58** WAIS: Not available	Total = 7.0 ± 1.4 DSST: Not available CMS Score: Not available DSB Test: Total = 7.0 ± 1.4 MIS (MoCA): Not available VRT: Not available LMT: Not available Composite: Not available WAIS: Not available
Attention	**Total**: n = 14 **TMT-A:** n = 9 **CRT**: n = 4 **SiRT:** n = 3 **DSF Test:** n = 5 **AI (MoCA):** n = 1 **5-CMT:** n = 1 **ACE-lll:** n = 1	**TMT-A:** Total = 28.6 ± 10.03 **CRT:** Total = 783.1 ± 167.5 **SiRT:** Total = 250.9 ± 56.7 **DSF Test:** Total = 7.2 ± 1.9 **AI (MoCA):** 16.3 ± 1.8 **5-CMT:** 370.5 **ACE-lll:** 13.58 ± 0.29	**Total =** 51.4 ± 6.1, Males = 55.1 ± 6.8, Females = 56.9 ± 6.3	**Total** = 115.5 ± 25.2 **TMT-A:** Total = 101.2 ± 12.9 **CRT:** Total = 114.3 ± 25.8 **SiRT:** Total = 94.7 ± 5.3 **DSF Test:** Total = 126.0 ± 38.04 **AI (MoCA):** Not available **5-CMT:** Not available **ACE-lll:**176.8 ± 8.6	Total = 5.7 ± 0.2 TMT-A: Total = 5.7 ± 0.4 CRT: Total = 6.1 ± 0.3 SiRT: Total = 5.8 DSF Test: Total = 7.0 ± 1.4 AI (MoCA): Not available 5-CMT: Not available ACE-lll: Not available
Intelligence	Total: n = 5 **IQ**: n = 3 **MR:** n = 1 **AFQT:** n = 1	**IQ:** Total = 102.3 ± 10.6 **MR:** Total = 18.1 **AFQT:** Total = 61.8 ± 0.9	**Total **= 54.8 ± 3.9	**Total **= 91.4 ± 11.2 **IQ:** Total = 100.6 ± 19.1 **MR:** Not available **AFQT:** Not available	Total = Not available IQ: Not available MR: Not available AFQT: Not available
Executive Function Letter Cancellation	Total: n = 3 **LSST**: n = 1 **LCCS**: n = 1 **ACE-Language:** n = 1	**LSST:** Total = 282 **LCCS:** Total = 50 ± 7.3 **ACE-Language:** Total = 21.63 ± 0.37	**Total **= 49.27 ± 2.15	Total = 97.5 ± 9.7 **LSST:** Not available **LCCS:** Total = 89.17 ± 9.77 **ACE-Langugae:** Total = 176.8 ± 9.7	Total = 5.8 LSST: Total = 5.8 LCCS: Not available ACE-Language: Not available
Verbal Fluency	Total: n = 10 **WFT**: n = 4 **VIS (MoCA):** n = 1 **BeDT:** n = 1 **BuDT:** n = 1 **BNT**: n = 1 **WRT**: n = 1 **ACE-VF:** n = 1	**WFT:** Total = 29.9 ± 5.3, Male = 25.67 ± 6.4, Female = 24.81 ± 6.2 **VIS (MoCA):** Total = 6.48 ± 0.92 **BeDT:** Male = 12, Female: 12 **BuDT:** Male = 7, Female: 6 **BNT:** Total = 28 ± 2 **WRT:** Total = 30.4 ± 5.6 **ACE-VF:** Total = 4.38 ± 0.28	**Total **= 54.5 ± 5.6, Male = 56.6 ± 7.1, Female = 56.2 ± 7.1	**Total **= 97.6 ± 25.5 **WFT:** Total = 86.2 ± 20.9 **VIS (MoCA):** Not available **BeDT:** Not available **BuDT:** Not available **BNT:** Not available **WRT:** Total = 129.5 ± 39.9 **ACE-VF:** Total = 176.8 ± 5.58	Total = Not available WFT: Not available VIS (MoCA): Not available BeDT: Not available BuDT: Not available BNT: Not available WRT: Not available ACE-VF: Not available
Processing Speed	**Total:** n = 21 **TMT-B:** n = 8 **TrB-A: n = 2** **STIT:** n = 5 **WMT:** n = 1 **CES**: n = 3 **RVP (CANTAB):** n = 1 **SCWT:** n = 1 **EIS (MoCA):** n = 1 **LT**: n = 1 **VSS:** n = 2 **SWME:** n = 1	**TMT-B:** Total = 94.6 ± 3.9 **TrB-A: Total = 0.81 ± 0.69** **STIT:** Total = 29.3 ± 7.9 **WMT:** Total = 2.3 ± 1.1 **CES:** Total = 48.9 ± 0.1, Male = 52.7 ± 14.4, Female = 54.7 ± 0.1 **RVP (CANTAB):** Total = 0.9 **SCWT:** Total = 19.1 **EIS (MoCA):** Total = 11.6 ± 1.4 **LT:** Male = 39.8 ± 17.8, Female = 45.5 ± 26.6 **VSS:** Male = 329.1 ± 78.4, Female = 348.58 ± 78.27 **SWME:** Total = 2.7 ± 1.8	**Total** = 53.1 ± 5.2, Male = 56.6 ± 7.1, Female = 56.2 ± 7.1	**Total** = 98.66 ± 26.53 **TMT-B:** Total = 89.74 ± 22.76 **TrB-A:** Not Available **STIT:** Total = 89.74 ± 22.76 **WMT:** Total = 94.4 ± 9.35 **CES:** Not Available **RVP (CANTAB):** Not Available **SCWT:** Not Available **EIS (MoCA):** Not available **LT:** Not available **VSS:** Not Available **SWME:** Not Available	Total = 7.0 ± 1.37, Male = 5.58 ± 0.19, Female = 5.52 ± 0.12 TMT-B: Total = 7.0 ± 1.37, STIT: Total = 7.0 ± 1.37 WMT: Not available CES: Male = 5.58 ± 0.19, Female = 5.52 ± 0.12 RVP (CANTAB): Not available SCWT: Not available EIS (MoCA): Not available LT: Not available
Global Cognition	**Total:** n = 27 **MMSE:** n = 17 **MoCA:** n = 9 **IQCODE:** n = 1 **CAMCOG:** n = 1 **NART:** n = 1 **IST:** n = 1 **BPP:** n = 1 **ACE:** n = 2 **HRS-CS:** n = 1 **MINT:** n = 1	**MMSE**: Total = 26.9 ± 1.3, Males = 28.5 ± 1.9, Females = 27.9 ± 2.8 **MoCA**: Total = 24.5 ± 3.9, Male = 25 ± 2.9, Females = 25.5 ± 2.9 **IQCODE**: Total = 43.4 ± 3.01 **CAMCOG:** Total = 90 **NART:** Total = 28 **IST:** Total = 32.4 **BPP:** Total = 46.9 **ACE:** Total = 87.5 ± 0.4 **HRS-CS:** Total = 14.31 ± 4.06, Male = 14.2 ± 4.15, Female = 14.44 ± 3.96 **MINT:** Total = 31	**Total **= 54.5 ± 5.6, Males = 58.7 ± 3.18, Females = 58.4 ± 3.2	**Total** = 97.5 ± 20.3, Males = 103.3 ± 27.1, Females = 101.6 ± 29.2 **MMSE:** Total = 97.1 ± 20.4, Males = 103.3 ± 27.1, Females = 101.6 ± 29.2 **MoCA:** Total = 102.9 ± 13.7 **IQCODE:** Total = 96.4 ± 13.8 **CAMCOG**: Total = 86.4 **NART:** Total = 86.4 **IST**: Not available **BPP:** Not available **ACE:** Total = 176.8 ± 8.6 **HRS-CS:** Not available **MINT:** Total = 176.8 ± 8.6	Total = 5.02 ± 0.8 MMSE: Total = 5.02 ± 0.8 MoCA: Not Available IQCODE: Not Available CAMCOG: Not Available NART: Not Available IST: Not available BPP: Not available ACE: Not available HRS-CS: Not available MINT: Not Available
Inductive Reasoning	**Total**: n = 2	**AH-4:** Total = 73.1, Male = 49.2 ± 9.5, Female = 42.9 ± 11.6	**Total **= 52.8, Males = 55.1 ± 5.9, Females = 55.3 ± 5.9	**Total **= 86.4	Not available
Psychomotor Speed	Total: n = 6	**SDMT:** Total = 54.3 ± 10.02, Female = 50.5	Total = 46.7 ± 2.6, Females = 50.1 ± 2.6	Total = 92.9 ± 1.7	Not available
Visuospatial Organisation	Total: n = 7 **BDT:** n = 1 **VIS MoCA:** n = 1 **Vr-D: **n = 1 **VSA:** n = 1 **HVOT**: n = 1 **CDT**: n = 1 **FC**: n = 1	**BDT:** Total = 25.6 ± 0.6 **VIS MoCA:** Total = 6.48 ± 0.92 **Vr-D: **Total = 8.62 ± 3.24 **VSA: Total = 11.65 ± 0.34** **HVOT: 25 ± 3** **CDT: **Total = 2.1 ± 1.1, Male = 28 ± 5, Female = 55 ± 10 **FC:** Total = 9.1 ± 7.8	Total = 51.2 ± 7.4	Total = 127.9 ± 35.7 **BDT:** Total = 92.9 ± 1.6 **VIS MoCA:** Not available **VSA:** Total = 176.8 ± 5.58 **HVOT:** Not available **Vr-D: **Not available **CDT:** Total = 129.5 ± 39.9 **FC:** Total = 129.5 ± 39.9	Total = Not available BDT: Not available VIS MoCA: Not available VSA: Not available HVOT: Not available Vr-D: Not available CDT: Not available FC: Not available
Temporal Orientation	Total: n = 2	Total = 6.6 ± 0.9	Total = 54.5 ± 7.1	Not available	Total = 6.95 ± 1.5

**Abbreviations**: *ACE*, Addenbrooke’s cognitive examination, *AH-4*, Alice Heim 4-I, *BDT:* Block Design Test; *BeDT:* Benson Delay Test; *BNT:* Boston Naming Test; *BP*, Blood Pressure, *BPP*, Børge Priens Prøve, *BuDT: Buschke Delay Test; CAMCOG*, Cambridge Cognition Examination, *CANTAB*, Cambridge Neuropsychological Test Automated Battery, *CDT:* Clock Drawing Test; *CERAD*, Consortium to Establish a Registry for Alzheimer’s Disease, *CMS*, Chinese Clinical Memory Scale, *CRT:* Choice Reaction Time; *CVLT*, California Verbal Learning Test *DSB*, Digit Span Backwards, *CES:* Composite Executive Score; *DSF*, Digit Span Forward, *DSST*, Digit Symbol Substitution Test, *EBM:* East Boston Memory Test; *EIS*, Executive Index Score, *GCA,* General Cognitive Ability; *HRS-CS*, U.S. Health and Retirement Study Composite Score, *IST*, Intelligenz-Struktur-Test, *IQCODE*, Informant Questionnaire on Cognitive Decline in the Elderly, *IQ*, Intelligence Quotient, *LCCS:* Letter Cancellation Composite Score; *LSST*, Letter Search Speed Test, *LT:* Labyrinth Test; *McNS:* McNair Survey; *MINT*, Multilingual Naming Test, *MIS:* Memory Index Score; *MoCA*, Montreal Cognitive Assessment, *MMSE*, Mini-Mental State Exam, *MR:* Mental Rotation Test; *MVT:* Mill Hill Vocabulary Test; *NART*, National Adult Reading Test, *PFT: Phonemic Fluency Test; RAVLT*, Rey Auditory Verbal Learning Test, *ROCF*, Rey–Osterreith complex figure, *RVP:* Rapid Visual Processing; *SCWT:* Stroop Colour Word Test; *SDMT*, Symbol Digits Modalities Test, *SCS:* Spatial Composite Score; *SFT:* Semantic Fluency Test; *SRT:* Selective Reminding Test; *SiRT*: Simple Reaction Time; *STIT:* Stroop Test (Interference Time); *STW:* Spot the Word Test; *TMT-A*, Trail making Test Part A, *TMT-B*, Trail making Test Part B, *TrB-A*, Trail making Test Difference between Part B and A, *VIS*, Visuospatial Index Score, *VRT:* Visual Reproduction Test; *VSS:* Visual Search Speed; *WFT:* Word Fluency Test; *WDS:* WAIS-IV Digit Sequencing; *WAIS*, Wechsler Adult Intelligence Scale; *WMT:* Word Matching Test; *5-CMT:* – Choice Movement Test.

Data are present as mean ± standard deviation or number.

**Table 2:** Summary of weighted FBG and HbA_1_c levels, and prevalence of diabetes and MetS across included studies (*reference studies applied*).

	FBG (mg/dl)	No Studies	No. Participants	HbA_1_c (mg/dl)	No Studies	No. Participants	Diabetes	No Studies	MeTS	No Studies
Total	93.8 ± 20	49	39,632	4.77 ± 0.67	21	21,739	With: 16,120 Without: 112,270	55	With: 1,729 Without: 10,474	7
Male	103.3 ± 27.2	3	902	5.59 ± 0.45	5	1,763	With: 1,527 Without: 10,785	11	With: 250 Without: 985	1
Female	96 ± 27.6	4	1,945	5.56 ± 0.41	5	1,735	With: 1,366 Without: 11,980	10	With: 919 Without:2,875	2

Data are presented as mean ± standard deviation or number.

Males and females had similar levels of mean FBG (103.3 ± 27.2 vs 96.0 ± 27.6 mg/dl) and HbA1c levels (5.6 ± 0.5 vs. 5.6 ± 0.4 mg/dl). 250 males and 919 females presented with MetS. A higher number of males were classified as diabetic than females (1,527 vs. 1,366).

### 3.6 Associations between midlife T2DM and cognition in later life

Studies assessing midlife T2DM and later life cognition were of moderate-to-high quality. Of the 7 longitudinal cohort studies, a negative relationship between midlife T2DM and later life cognition was reported across several domains, including memory (n = 2, 29%), executive function (n = 3, 50%), global cognition (n = 4, 57%), and inductive reasoning (n = 1, 50%). In the case of the individual cohort studies, no relationship was found between midlife T2DM and cognitive function at later life among the domains of memory (n = 5, 71%), attention (n = 2, 100%), executive function (n = 3, 50%), processing speed (n = 1, 100%), global cognition (n = 3, 43%), visuospatial organisation (n = 1, 100%), and inductive reasoning (n = 1, 50%) (see Table 3).

**Table 3:** Summary of longitudinal studies with negative or null relationship between diabetes and cognitive measures at later life.

Author	Year	Setting	Study Quality	Cognitive Variables	Relationship
Anstey et al.	2014	PATH, Australia	Moderate	Memory, attention, executive function, processing speed, global cognition	0 Diabetes
Bancks et al.	2017	ARIC, USA	High	Memory, executive function, global cognition	- Diabetes (executive function, global cognition)
Bangen et al.	2013	Framingham, USA	High	Memory, executive function, global cognition, and visuospatial organisation	- Diabetes (executive function)
Bayes-Marin et al.	2020	Edad con Salud, Spain	High	Memory	- Diabetes (Memory)
Blodgett et al.	2020	MRC NSHD, UK	Moderate	Verbal Memory	0 Diabetes
Cherbuin et al.	2009	PATH, Australia	High	Global Cognition	- Diabetes (global cognition)
Dixon et al.	2021	SWAN, USA	Moderate	Executive function, working and episodic memory	0 Diabetes (working memory) - Diabetes (executive function, episodic memory)
Kaffashian et al.	2013	Whitehall II, UK	High	Memory, executive function, attention, global cognition, inductive reasoning	- Diabetes (global cognition)
Nunley et al.	2017	Pittsburgh Epidemiology of Diabetes Complications Study; USA	High	Memory, attention, executive function, global cognition, intelligence, and psychomotor speed	0 Diabetes
Tuligenga et al.	2014	Whitehall II study; UK	Moderate	Memory, executive function, inductive reasoning, and global cognition	- Diabetes (Memory, inductive reasoning, global cognition)

0 = No relationship; - = Negative relationship.

Of the individual longitudinal studies (n = 3), two studies (66%) reported no relationship with memory, attention, executive function, global cognition, intelligence, and psychomotor speed [29,30], and one study (33%) reported a negative relationship with memory [31] (see Table 3).

Overall, the majority of longitudinal studies (n = 7; 70%) reported negative relationships between midlife T2DM and later cognitive function, specifically executive function, and global cognition. However, conflicting findings were reported for memory.

### 3.7 Associations between measures of T2DM and its metabolic correlates and cognition at midlife

Table 2 and supporting material in the supplementary file provide details of the relationships between T2DM, MetS, FBG, and HbA1c levels and measures of cognitive function. Most studies (n = 70; 66%) reported no relationship between T2DM and cognitive function, including memory (n = 29, 66%), attention (n = 16, 76%), executive function (n = 26, 67%), global cognition (n = 25, 74%), psychomotor speed (n = 4, 80%) and visuospatial organisation (n = 7, 86%). All studies that included intelligence (n = 2) or temporal orientation (n = 1) reported no relationship with T2DM.

The majority of studies reported no relationship between FBG and memory (n = 26, 79%), attention (n = 15, 83%), executive function (n = 24, 80%), global cognition (n = 26, 87%), and psychomotor speed (n = 4, 80%), whereas all studies that included inductive reasoning (n = 2), intelligence (n = 5), temporal orientation (n = 1) and visuospatial organisation (n = 7) reported no relationship.

For HbA1c, studies predominately reported no relationship with memory (n = 9, 69%), attention (n = 6, 75%), executive function (n = 9, 64%) and global cognition (n = 5, 56%), whereas all studies that included intelligence (n = 1), psychomotor speed (n = 1), and visuospatial organization (n = 1) reported no relationship.

For MeTS, findings were conflicting. A negative relationship was reported with memory (n = 3, 75%), attention (n = 1, 50%), executive function (n = 2, 66%), global cognition (n = 1, 25%), inductive reasoning (n = 1, 100%), and psychomotor speed (n = 2, 66%), whereas the single study that reported on intelligence reported no relationship. There were no discernible differences in findings based on study design with most studies reporting no qualitative relationship between metabolic measures and cognitive function at midlife (individual cohorts: n = 23/32, 72%; large cohorts: n = 6/10, 60%). Studies of moderate and high quality consistently reported no relationship (n = 82, 80%) whereas findings from low quality studies were inconsistent.

### 3.8 Meta-analysis

**Fig 2:**
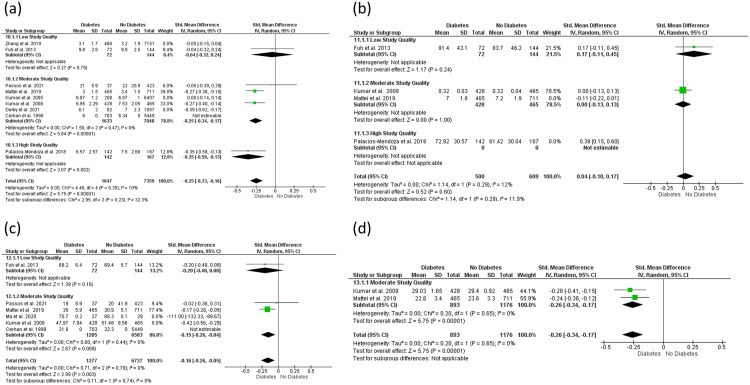
Forest plot examining the overall effect of diagnosed T2DM versus no diagnosis. **(a)** Memory function, **(b)** Attention, **(c)** Executive function, **(d)** Global Cognition.

**Fig 3:**
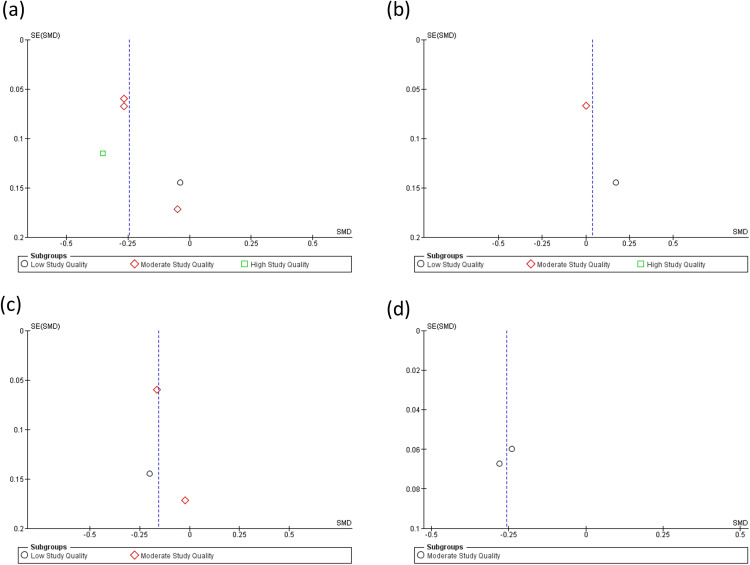
Funnel plots of meta-analysis assessing the differences between those with diabetes versus those without diabetes: (a) Memory, (b) Attention, (c) Executive Function, (d) Global Cognition. *SMD* = standardised mean difference; *SE* = standard error.

Meta-analysis was conducted to assess the relationship between midlife T2DM and midlife cognition; analysis was not possible for later life cognition as limited or no raw cognitive data were available. Ten studies across four cognitive domains (memory, executive function, attention, and global cognition) were suitable for meta-analysis. A total of 3,586 participants had a diagnosis of T2DM while 30,167 participants were absent of T2DM. T2DM had a negative effect on memory function across studies of low, moderate, and high quality (n = 6) [MD = −0.25; 95% CI = −0.33 to −0.16; I^2^ = 10%]. T2DM had no effect on attention across all levels of study quality (n = 2) with high heterogeneity [MD = 0.04; 95% CI = −0.10 to 0.17; I^2^ = 12%]. T2DM had a negative impact on executive function across studies of low and moderate quality (n = 4) [MD = −0.16; 95% CI = −0.26 to −0.05; I^2^ = 0%], and global cognition across studies of moderate quality (n = 2) [MD = −0.26; 95% CI = −0.34 to −0.17; I^2^ = 0%] (see Figs 2 and 3).

## 4. Discussion

The present review systematically assessed the impact of midlife T2DM on cognitive function at mid and later life. Findings on the relationship between midlife T2DM and cognitive function at later life varied according to study type. The majority of longitudinal cohort studies (66%) reported a negative relationship with memory, executive function, and global cognition whereas independent longitudinal studies were inconsistent, with both negative and null findings. Despite most studies reporting no association between midlife T2DM and cognition at midlife, meta-analytic findings indicated a negative impact on memory, executive function, and global cognition. No definitive associations could be made between FBG and HbA_1_c levels and cognition, while conflicting results were observed for MetS, due to scarcity of relevant studies.

Findings from this review suggest that some, but not all, cognitive domains are negatively impacted in later life by midlife T2DM, specifically memory, executive function, and global cognition. This is supported by a previous review, which further reported that exposure to pre-diabetes during mid-life was associated with a significant decline in global cognition in later life [32]. Similar to Papunen and colleagues, the associations between T2DM and cognitive function were not always clear, potentially due to the lack of a single standardised cognitive test, resulting in a large variation in types of cognitive assessment. However, findings from both reviews suggest a domain-specific cognitive decline, albeit at different rates, rather than global cognitive decline. This is of particular interest as previous research using imaging data suggests that cognitive decline in middle-aged individuals with diabetes is tied to higher grey matter volume loss in cortical regions linked to executive and memory functions [33]. However, our review did not include imaging-based studies, therefore direct inference cannot be made. Our findings further support the recent publications from the Lancet Commission on dementia prevention, intervention, and care which indicated that up to 45% of all dementia cases are linked to modifiable risk factors [34,35], including risk factors such as T2DM. In the context of an ageing population, intervention strategies at early and critical timepoints, such as midlife, are warranted to reduce the risk of later life cognitive decline among those with T2DM.

Although most studies found no discernible relationship between T2DM and midlife cognition, our meta-analysis suggests that memory, executive function, and global cognition, but not attention, are negatively impacted by T2DM. While our findings align with a previous meta-analysis of 24 studies, there were variations in effect sizes and study populations. This is potentially due to the difference in selected age ranges as we used a younger classification of midlife, as per WHO (40–65 years), as opposed to the 50–85 year age range used by Palta and colleagues [36]. Recent systematic evidence based on nine included studies reported an increased risk of cognitive impairment based on measures of global cognition in patients with T2DM due to glucose variability, although no defined age range was employed rendering it difficult to generalise results in comparison to those presented in this review [37]. Moreover, as indicated from a systematic review by You et al., the prevalence of MCI in T2DM was calculated at 45% across 12 studies [19]. Subgroup analysis by age in that review revealed a lower prevalence in T2DM patients above 60 years compared to those below 60 years, highlighting that midlife can provide a potential therapeutic window of intervention in the preservation of later-life brain health, in line with our present results. Our findings also broadly agree with those of Pelimanni and Jekhonen [18] whose meta-analysis revealed a poorer performance of midlife patients with T2DM relative to controls in the domains of processing speed, attention/concentration, executive functions and working memory. The subtle differences between the studies relate to the differing classification of cognitive tasks within broader cognitive domains. Such differences between studies have been highlighted in a recent important and timely ‘review of reviews’ that included seven meta-analyses and 2 narrative systematic reviews of the relationship between T2DM and cognitive performance [38]. This review addressed the difficulties in drawing conclusions from analysis of multiple systematic reviews and meta-analyses due to heterogeneity in methodology, quality and results of such studies. The authors concluded that while T2DM was associated with lower cognitive performance across several cognitive domains, high-quality meta-analyses on this subject that follow PRISMA guidelines and include sensitivity and subgroup analysis are still needed [38]. We believe that the present review fits these criteria and thus makes an important contribution to the literature.

Meta-analytic findings of domain-specific cognitive decline (memory, executive function, and global cognition) at midlife are in line with our findings at later life, supporting previous research reporting an increased risk of accelerated cognitive decline and dementia in individuals with T2DM [39]. While multiple cross-sectional and longitudinal investigations support this relationship [40], the underlying cause(s) and the timeframe at which T2DM influences cognitive aging, particularly in select domains, remains unclear. Although findings from our meta-analysis are limited due to the small number of studies included (n = 10), to our knowledge it is the first to analyse cognition and T2DM at both midlife and later life. A further factor that may influence cognitive function in this population is that T2DM is rarely present in isolation, with 70.9% of those diagnosed T2DM presenting with the comorbidity of elevated blood pressure [41], also known to negatively impact cognitive function at midlife [20].

Most studies included in the present review do not provide evidence of a relationship between FBG or HbA_1_c and cognition. This is in line with previous systematic evidence reporting that approximately 80% of cross sectional studies found no relationship between FBG and cognition, while the limited number of studies that reported a significant relationship were inconsistent, with bell-shaped associations reported in less than 10% of included studies [42]. Similarly, 53% of cross-sectional studies report no association between HbA_1_c concentrations and memory, executive function, or attention [42]. There is a need for a better understanding of the long-term effects of metabolic correlates of midlife T2DM to enable more insightful conclusions to be drawn about the trajectories of cognitive decline with age, for suitable intervention and management strategies to be put in place.

We acknowledge several limitations of the present review. The methods of test administration and follow-up times varied considerably across all studies, reducing ability for cross-study comparison. The inclusion of cofounding variables in individual study analysis such as age, hypertension, dyslipidemia, and educational attainment limited our ability to extract data for quantitative analysis. The ability to generalise results may be hindered by differences in the number of males and females across studies as well as ethnic and racial disparities. Thus we highlight here a notable gap in the existing literature due to the limited stratified reporting of results by sex or geographic region, which restricts the ability to explore these factors as potential sources of heterogeneity in meta-analyses. Furthermore, several of our association results were based on small numbers of studies (<3). As per our predefined eligibility criteria, we included only studies that met our strict methodological and reporting standards to minimise bias and ensure comparability across studies. We emphasise, therefore, that these findings should be interpreted with caution and viewed as preliminary evidence, which may help to identify gaps in the literature and inform priorities for future research. Further to this point, the use of cross-sectional studies in this review does not allow us to infer causality in the impact of T2DM on measures of cognitive function, although an association has been identified that warrants further investigation. Therefore, follow-up investigations including longitudinal studies, would be required to establish causality. Meta-analyses were limited due to the use of non-standardised neurocognitive tests and the absence of raw data in individual studies. Consequently, the meta-analysis should be viewed as a sub-analysis. Minor discrepancies between the qualitative and quantitative findings in this review largely reflect differences in the number and completeness of studies eligible for pooling, rather than substantive contradictions in the underlying evidence. It is also relevant to note that our review does not explicitly link reductions in cognitive function to any specific pathological factor associated with T2DM. A further limitation relates to the small size of the mean differences calculated across the four broad cognitive domains, even though these differences were statistically significant in three of four domains. These differences are smaller than mean clinically important differences reported in the literature [43], suggesting that they may not necessarily translate into clinically meaningful changes. However, small but significant changes consistent across studies may still be relevant at the population level or in early stages of cognitive decline. Finally, all cognitive tests were grouped by cognitive domain, while most tests of cognition will in fact assess several cognitive domains.

## 5. Conclusion

T2DM and associated cognitive decline pose a significant burden to patients, carers, and healthcare professionals. Findings from this review suggest a negative relationship between midlife T2DM and cognitive function across specific domains at later life. Despite qualitative findings suggesting no relationship with cognitive function at midlife, findings from our meta-analysis suggest a negative effect, specifically on memory, executive function, and global cognition. The downstream negative implications of midlife T2DM on cognitive function at later life are cause for concern given the growing prevalence of dementia and cognitive dysfunction, especially in the context of an ageing population. Future studies should aim to establish efficacious and evidence-based intervention strategies during this vulnerable period of ageing to promote healthy cognitive ageing in those with T2DM.

## Supporting information

S1 Checklist PRISMA checklist. (DOCX)

S1 File Search strategy and 7 supplementary tables. (DOCX)
