# Supplementary material for: The influence of type 2 diabetes and its metabolic correlates in middle-aged adults on cognition at mid and later life; A systematic review and meta-analysis
Source: PLoS One. 2025 Dec 5;20(12):e0327408. doi: 10.1371/journal.pone.0327408 (PMC12680190; doi:10.1371/journal.pone.0327408)
Supplement: S1 Checklist — (DOCX) [file pone.0327408.s001.docx]

| **Section and Topic** | **Item #** | **Checklist item** | **Location where item is reported** |
| --- | --- | --- | --- |
| **TITLE** | | |  |
| Title | 1 | Identify the report as a systematic review. | Manuscript Location: Title page  Page: 1  Details: "The influence of type 2 diabetes and its metabolic correlates in middle-aged adults on cognition at mid and later life; a systematic review and meta-analysis." |
| **ABSTRACT** | | |  |
| Abstract | 2 | See the PRISMA 2020 for Abstracts checklist. | Manuscript Location: Abstract section  Page: 2  Details: The abstract contains structured elements: Introduction, Methods, Results, and Discussion. It includes the number of studies reviewed, key findings, and conclusions about the impact of midlife T2DM on cognition. |
| **INTRODUCTION** | | |  |
| Rationale | 3 | Describe the rationale for the review in the context of existing knowledge. | Manuscript Location: Introduction section  Page: 4  Details: Discusses the growing prevalence of T2DM in middle-aged adults and the potential impacts on cognitive function, highlighting the need for a comprehensive review. |
| Objectives | 4 | Provide an explicit statement of the objective(s) or question(s) the review addresses. | Manuscript Location: Introduction section, final paragraph  Page: 5  Details: States the aim to systematically assess the impact of midlife T2DM on cognitive function across different domains from midlife into later life. |
| **METHODS** | | |  |
| Eligibility criteria | 5 | Specify the inclusion and exclusion criteria for the review and how studies were grouped for the syntheses. | Manuscript Location: Methods section, Eligibility Criteria subsection  Page: 6  Details: Lists inclusion criteria based on age, T2DM diagnosis, cognitive outcomes, and exclusion criteria regarding pre-existing cognitive impairments. |
| Information sources | 6 | Specify all databases, registers, websites, organisations, reference lists and other sources searched or consulted to identify studies. Specify the date when each source was last searched or consulted. | Manuscript Location: Methods section, Search Strategy subsection  Page: 5  Details: Specifies databases searched (e.g., MEDLINE, PubMed, Web of Science, CINAHL) and the search cut-off date (December 2023). |
| Search strategy | 7 | Present the full search strategies for all databases, registers and websites, including any filters and limits used. | Manuscript Location: Supplementary Material  Page: 29  Details: Detailed search strategy provided in the supplementary file, including terms and combinations used. |
| Selection process | 8 | Specify the methods used to decide whether a study met the inclusion criteria of the review, including how many reviewers screened each record and each report retrieved, whether they worked independently, and if applicable, details of automation tools used in the process. | Manuscript Location: Methods section, Search Strategy subsection  Page: 6  Details: Describes the use of Covidence for title and abstract screening, full-text review, and conflict resolution by third author if needed. |
| Data collection process | 9 | Specify the methods used to collect data from reports, including how many reviewers collected data from each report, whether they worked independently, any processes for obtaining or confirming data from study investigators, and if applicable, details of automation tools used in the process. | Manuscript Location: Methods section, Data Extraction subsection  Page: 6  Details: Mentions data extraction according to STROBE guidelines, with independent extraction by two authors and use of Endnote for database management. |
| Data items | 10a | List and define all outcomes for which data were sought. Specify whether all results that were compatible with each outcome domain in each study were sought (e.g. for all measures, time points, analyses), and if not, the methods used to decide which results to collect. | Manuscript Location: Methods section, Data Extraction subsection  Page: 6,7  Details: Specifies the variables extracted, including cognitive function measures, T2DM indicators, and demographic details. |
|  | 10b | List and define all other variables for which data were sought (e.g. participant and intervention characteristics, funding sources). Describe any assumptions made about any missing or unclear information. | Manuscript Location: Methods section, Data Extraction subsection  Page: 6,7 |
| Study risk of bias assessment | 11 | Specify the methods used to assess risk of bias in the included studies, including details of the tool(s) used, how many reviewers assessed each study and whether they worked independently, and if applicable, details of automation tools used in the process. | Manuscript Location: Methods section, Risk of Bias subsection  Page: 7  Details: Utilizes the AXIS tool to assess study quality, with independent assessments by two authors and resolution of disagreements by a third author. |
| Effect measures | 12 | Specify for each outcome the effect measure(s) (e.g. risk ratio, mean difference) used in the synthesis or presentation of results. | Manuscript Location: Methods section, Statistical Analysis subsection  Page: 7  Details: Describes using mean differences and 95% confidence intervals for cognitive outcomes in meta-analyses. |
| Synthesis methods | 13a | Describe the processes used to decide which studies were eligible for each synthesis (e.g. tabulating the study intervention characteristics and comparing against the planned groups for each synthesis (item #5)). | Manuscript Location: Methods section, Data Extraction subsection  Page:6  Details: Specifies the variables extracted, including cognitive function measures, T2DM indicators, and demographic details. |
|  | 13b | Describe any methods required to prepare the data for presentation or synthesis, such as handling of missing summary statistics, or data conversions. | Manuscript Location: Methods section, Data Extraction subsection  Page:6 |
|  | 13c | Describe any methods used to tabulate or visually display results of individual studies and syntheses. | Manuscript Location: Methods section, Data Extraction subsection  Page:6 |
|  | 13d | Describe any methods used to synthesize results and provide a rationale for the choice(s). If meta-analysis was performed, describe the model(s), method(s) to identify the presence and extent of statistical heterogeneity, and software package(s) used. | Manuscript Location: Methods section, Data Extraction subsection  Page:6 |
|  | 13e | Describe any methods used to explore possible causes of heterogeneity among study results (e.g. subgroup analysis, meta-regression). | Manuscript Location: Methods section, Data Extraction subsection  Page:6,7 |
|  | 13f | Describe any sensitivity analyses conducted to assess robustness of the synthesized results. | Manuscript Location: Methods section, Data Extraction subsection  Page:6,7 |
| Reporting bias assessment | 14 | Describe any methods used to assess risk of bias due to missing results in a synthesis (arising from reporting biases). | Manuscript Location: Methods section, Risk of Bias subsection  Page: 7  Details: Includes discussion of assessing publication bias through visual inspection of funnel plots and statistical tests. |
| Certainty assessment | 15 | Describe any methods used to assess certainty (or confidence) in the body of evidence for an outcome. | Manuscript Location: Methods section, Risk of Bias subsection  Page: 7  Details: Briefly mentions assessing the overall quality and certainty of the evidence but does not specify a particular tool like GRADE. |
| **RESULTS** | | |  |
| Study selection | 16a | Describe the results of the search and selection process, from the number of records identified in the search to the number of studies included in the review, ideally using a flow diagram. | Manuscript Location: Results section, Study Selection subsection and Figure 1  Page:9  Details: Provides a flow diagram (Figure 1) and narrative description of the screening process. |
|  | 16b | Cite studies that might appear to meet the inclusion criteria, but which were excluded, and explain why they were excluded. | Manuscript Location: Results section, Study Selection subsection and Figure 1  Page:9 |
| Study characteristics | 17 | Cite each included study and present its characteristics. | Manuscript Location: Results section, Study Characteristics subsection  Page:10  Details: Describes included studies with a summary table in supplementary material. |
| Risk of bias in studies | 18 | Present assessments of risk of bias for each included study. | Manuscript Location: Results section, Risk of Bias subsection  Page:10  Details: Summarizes risk of bias assessments with detailed results available in supplementary materials. |
| Results of individual studies | 19 | For all outcomes, present, for each study: (a) summary statistics for each group (where appropriate) and (b) an effect estimate and its precision (e.g. confidence/credible interval), ideally using structured tables or plots. | Manuscript Location: Results section, Cognitive Outcomes subsection  Page:10,11,12  Details: Provides summary data and effect estimates with forest plots for meta-analyses in supplementary material. |
| Results of syntheses | 20a | For each synthesis, briefly summarise the characteristics and risk of bias among contributing studies. | Manuscript Location: Results section  Page:12,13 |
|  | 20b | Present results of all statistical syntheses conducted. If meta-analysis was done, present for each the summary estimate and its precision (e.g. confidence/credible interval) and measures of statistical heterogeneity. If comparing groups, describe the direction of the effect. | Manuscript Location: Results section, Meta-Analysis subsection  Page:13  Details: Includes meta-analysis results with confidence intervals and heterogeneity measures (I²). |
|  | 20c | Present results of all investigations of possible causes of heterogeneity among study results. | Manuscript Location: Results section, Meta-Analysis subsection  Page:13 |
|  | 20d | Present results of all sensitivity analyses conducted to assess the robustness of the synthesized results. | Manuscript Location: Results section, Meta-Analysis subsection Page:13 |
| Reporting biases | 21 | Present assessments of risk of bias due to missing results (arising from reporting biases) for each synthesis assessed. | Manuscript Location: Results section, Reporting Bias subsection  Page:10  Details: Discusses potential publication bias and includes funnel plots in supplementary material. |
| Certainty of evidence | 22 | Present assessments of certainty (or confidence) in the body of evidence for each outcome assessed. | Manuscript Location: Results section, Certainty of Evidence subsection  Page:10  Details: Briefly discusses the certainty of the evidence in terms of study quality and consistency of results. |
| **DISCUSSION** | | |  |
| Discussion | 23a | Provide a general interpretation of the results in the context of other evidence. | Manuscript Location: Discussion section  Page:13-15  Details: Interprets findings in the context of existing literature, discusses limitations, and suggests directions for future research. |
|  | 23b | Discuss any limitations of the evidence included in the review. | Manuscript Location: Discussion section, Limitations subsection  Page:16  Details: Addresses limitations related to study designs, heterogeneity, and potential biases. |
|  | 23c | Discuss any limitations of the review processes used. | Manuscript Location: Discussion section, Limitations subsection  Page:16  Details: Addresses limitations related to study designs, heterogeneity, and potential biases. |
|  | 23d | Discuss implications of the results for practice, policy, and future research. | Manuscript Location: Discussion section & Conclusion  Page:16 |
| **OTHER INFORMATION** | | |  |
| Registration and protocol | 24a | Provide registration information for the review, including register name and registration number, or state that the review was not registered. | Manuscript Location: Methods section, Registration subsection  Page: 4  Details: Provides PROSPERO registration number. |
|  | 24b | Indicate where the review protocol can be accessed, or state that a protocol was not prepared. | Manuscript Location: Methods section, Registration subsection  Page: 5  Details: Provides PROSPERO registration number & associated link to access protocol |
|  | 24c | Describe and explain any amendments to information provided at registration or in the protocol. | Manuscript Location: Methods section, Protocol subsection  Page: 5  Details: Provides all relevant updates associated with registered protocol on PROSPERO |
| Support | 25 | Describe sources of financial or non-financial support for the review, and the role of the funders or sponsors in the review. | Manuscript Location: Funding section  Page: 1  Details: Lists funding sources and declares any conflicts of interest. |
| Competing interests | 26 | Declare any competing interests of review authors. | Manuscript Location: Conflict of Interest section  Page: 1  Details: Lists funding sources and declares any conflicts of interest. |
| Availability of data, code and other materials | 27 | Report which of the following are publicly available and where they can be found: template data collection forms; data extracted from included studies; data used for all analyses; analytic code; any other materials used in the review. | Appendices: Additional supporting information, such as detailed search strategies and extracted data tables, is included in the supplementary materials.  Page: 28 |

*From:*  Page MJ, McKenzie JE, Bossuyt PM, Boutron I, Hoffmann TC, Mulrow CD, et al. The PRISMA 2020 statement: an updated guideline for reporting systematic reviews. BMJ 2021;372:n71. doi: 10.1136/bmj.n71
