# Supplementary material for: The influence of type 2 diabetes and its metabolic correlates in middle-aged adults on cognition at mid and later life; A systematic review and meta-analysis
Source: PLoS One. 2025 Dec 5;20(12):e0327408. doi: 10.1371/journal.pone.0327408 (PMC12680190; doi:10.1371/journal.pone.0327408)
Supplement: S1 File — (DOCX) [file pone.0327408.s002.docx]

# **List of Supplementary Information**

**Appendix A.1 Search Strategy**

**Supplemental Table B.1.** Summary of all studies with negative relationships between diabetes, associated metrics, and cognitive measures at midlife.

**Supplemental Table B.2.** Summary of studies with no relationship between diabetes, associated metrics, and cognitive measures at midlife.

**Supplemental Table B.3.** Summary of negative relationships between diabetes metrics and cognition at midlife by study design and quality.

**Supplemental Table B.4.** Critical appraisal of included studies using the AXIS tool.

**Supplemental Table B.5.** List of reference studies used for longitudinal cohort studies.

**Supplemental Table B.6.** Diabetes status, associated metrics, and cognitive measures across all included studies.

**Supplemental Table B.7.** List of Cognitive tests used across all cognitive domains.

## **Appendix A.1 Search Strategy**

Searches were run on 4 separate occasions, 23/02/21, 19/04/22, 19/12/23 and 09/12/24.

### **EMBASE**

('cognitive defect'/exp OR 'cognition'/exp) AND 'risk factor'/exp

((cognition OR cognitive) NEAR/2 health*):ti,ab

((cognition OR cognitive) NEAR/2 function*):ti,ab

((Cognitive OR cognition OR memory) NEAR/2 (impair* OR disorder* OR defect* OR decline?)):ti,ab

((dementia OR Alzheimer?) NEAR/3 risk*):ti,ab

#1 OR #2 OR #3 OR #4 OR #5

'diabetes mellitus'/exp

(diabetes OR 'diabetes mellitus' OR diabetic*):ti,ab

'cholesterol'/exp OR 'total cholesterol level'/exp

(cholesterol OR cholesterine OR cholesterin):ti,ab

#7 OR #8 OR #9 OR #10

'middle aged'/exp

('middle age?' OR middleaged or midlife OR ‘mid life’ OR ‘mid age’ OR ‘aged 40–65’ OR ‘middle years’):ti,ab

#12 OR #13

'cohort analysis'/exp

'longitudinal study'/exp

'prospective study'/exp

'follow up'/exp

Cohort?:ti,ab

'case control study'/exp

(case* and control*):ti,ab

#15 OR #16 OR #17 OR #18 OR #19 OR #20 OR #21

#6 AND #11 AND #14 AND #22 AND [english]/lim

('conference abstract' OR 'conference report' OR letter OR editorial):it

#23 NOT #24

### **Medline**

(exp Cognition Disorders/ OR exp cognition/) AND Risk Factors/

((cognition OR cognitive) adj2 health*).ti,ab.

((cognition OR cognitive) adj2 function*).ti,ab.

((Cognitive OR cognition OR memory) adj2 (impair* OR disorder* OR defect* OR decline?)).ti,ab.

((dementia OR Alzheimer?) adj3 risk*).ti,ab.

or/1-5

exp Diabetes Mellitus/

(diabetes OR diabetes mellitus OR diabetic*).ti,ab.

exp Cholesterol/ OR Hypercholesterolemia/

(cholesterol OR cholesterine OR cholesterin).ti,ab.

or/7-10

Middle Aged/

(middle age? OR middleaged or midlife OR mid life OR mid age OR middle years).ti,ab.

or/12-13

exp cohort studies/

cohort$.tw.

controlled clinical trial.pt.

epidemiologic methods/

limit 18 to yr=1971-1988

or/15-17,19

6 AND 11 AND 14 AND 20

## **Web of Science**

TS =(((cognition OR cognitive) NEAR/2 health*) OR ((cognition OR cognitive) NEAR/2 function*) OR ((Cognitive OR cognition OR memory) NEAR/2 (impair* OR disorder* OR defect* OR decline?)) OR ((dementia OR Alzheimer?) NEAR/3 risk*))

TS =(diabetes OR “diabetes mellitus” OR diabetic* OR cholesterol OR cholesterine OR cholesterin)

TS =(“middle age?” OR middleaged or midlife OR “mid life” OR “mid age” OR “aged 40–65” OR “middle years”)

TS =(“cohort analysis” OR “longitudinal study” OR “prospective study” OR “follow up” OR Cohort* OR “case control stud*” OR (case* and control*))

#1 AND #2 AND #3 AND #4

## **CINAHL**

(MH "Cognition In Old Age" OR MH "Cognition Disorders In Old Age" ) AND (MH "Risk Factors")

TI ((cognition OR cognitive) N2 health*) OR AB ((cognition OR cognitive) N2 health*)

TI ((cognition OR cognitive) N2 function*) OR AB ((cognition OR cognitive) N2 function*)

TI ((Cognitive OR cognition OR memory) N2 (impair* OR disorder* OR defect* OR decline*)) OR AB ((Cognitive OR cognition OR memory) N2 (impair* OR disorder* OR defect* OR decline*))

TI ((dementia OR Alzheimer*) N3 risk*) OR AB ((dementia OR Alzheimer*) N3 risk*)

S1 OR S2 OR S3 OR S4 OR S5

(MH "Diabetes Mellitus+")

TI (diabetes OR “diabetes mellitus” OR diabetic*) OR AB (diabetes OR “diabetes mellitus” OR diabetic*)

(MH "Cholesterol+") OR (MH "Cholesterol, Dietary") OR (MH "Embolism, Cholesterol")

TI (cholesterol OR cholesterine OR cholesterin) OR AB (cholesterol OR cholesterine OR cholesterin)

S7 OR S8 OR S9 OR S10

(MH "Middle Age")

TI (“middle age*” OR middleaged or midlife OR “mid life” OR “mid age” OR “aged 40–65” OR “middle years”) OR AB (“middle age*” OR middleaged or midlife OR “mid life” OR “mid age” OR “aged 40–65” OR “middle years”)

S12 OR S13

(MH "Prospective Studies+")

(MH "Case Control Studies+")

(TI (case or cases) n5 TI (control or controls)) OR (AB (case or cases) n5 AB (control or controls))

(TI (case or cases) n3 TI (matched)) OR (AB (case or cases) n3 AB (matched)) OR TI (control group*)

TI (cohort N3 (stud* OR analysis)) OR AB (cohort N3 (stud* OR analysis))

TI (observational N3 (stud*)) OR AB (observational N3 (stud*))

TI (“longitudinal stud*”) OR AB (“longitudinal stud*”)

S15 OR S16 OR S17 OR S18 OR S19 OR S20 OR S21

S6 AND S11 AND S14 AND S22

***Supplemental Table B.1.*** Summary of all studies with negative relationships between diabetes, associated metrics, and cognitive measures at midlife.

| Author | Year | Study design | Setting | Study quality | Participants | Cognitive variables | Relationship | |
| --- | --- | --- | --- | --- | --- | --- | --- | --- |
| Almani et al. | 2021 | Retrospective cohort study | Liaquat University Hospital, Jamshoro | Low | Total (T2DM) = 278 (males = 155, females = 123)  Age (Total) = 54.7 ± 5.4 | Global Cognition | | HbA_1_c: - (Global cognition) |
| Anstey et al. | 2014 | Longitudinal | PATH, Australia | Moderate | Males= 1,017, females= 1,135, Total= 2,152  Age= 42.6 (1.6) | Memory, attention, executive function, processing speed, global cognition | | Diabetes: - (memory, executive function, global cognition, Attention) |
| Babaei et al. | 2013 | RCT | Iran | Low | n= 52 (28 patients and 24 controls) Age= 57.1 (5.9) | Memory | | MeTS: - (memory) |
| Backestrom et al. | 2015 | Retrospective, cross sectional | Betula Prospective Cohort, Sweden | Moderate | n= 291 (males= 127, females= 164) Age= 50.7 (8) [males= 50.3 (8), females= 51 (8.1)] | Memory | | FBG: - (episodic memory – women) |
| Bancks et al. | 2017 | Retrospective, longitudinal | ARIC, USA | High | Visit 2  n= 10,133 (males= 4,458, females = 5,674)  Age= 56.6 (5.7) | Memory, executive function | | FBG: - (memory, executive Function)  HbA_1_c: - (memory, executive function, global cognition) |
| Bangen et al. | 2013 | Cross sectional, longitudinal | Framingham, USA | High | n= 1,436 (males= 660, females= 775) Age= 54 (9) | Memory, executive function, global cognition, and visuospatial organisation | | Diabetes: - (memory, attention, visuospatial organisation) |
| Bayes-Marin et al. | 2020 | Longitudinal | Edad con Salud, Spain | High | n= 633 (males= 304, females= 329) Age= 56.6 | Memory | | Diabetes: - (memory) |
| Cerhan et al. | 1998 | Longitudinal | ARIC; USA | Moderate | Total: n = 13913  Age = 45-64 | Memory, executive function, psychomotor speed | | - Diabetes (Memory, executive function) |
| Cherbuin et al. | 2009 | Prospective, longitudinal | PATH, Australia | High | Wave 2  n= 2,018 (males= 1022, females= 996)  Age= 62.5 (1.5) | Global cognition | | Diabetes: - (global cognition) |
| Christman et al. | 2011 | Prospective | ARIC, USA | Moderate | n= 8,958 (males= 3,943, females= 5,015)  Age= 56.5 (5.6) | Memory and executive function | | Diabetes: - (executive function, processing speed)  HbA_1_c: - (memory, executive function) |
| Creavin et al. | 2012 | Prospective, Longitudinal | Caerphilly prospective, Wales | Moderate | Phase 1  n= 2,512  Age= 52.8 (SD) | Attention, global cognition, inductive reasoning | | Diabetes: - (inductive reasoning)  MeTS: - (global cognition, inductive reasoning) |
| Dearborn et al. | 2014 | Cross sectional, longitudinal | ARIC, USA | High | n= 10,495  Age: Males = 54.7 (5.6), Females = 54.8 (5.5) | Memory, executive function | | Diabetes: - (memory, executive function, processing speed)  FBG: - (memory, executive function, processing speed)  MeTS: - (memory, executive function, processing speed) |
| Debette et al. | 2011 | Prospective | Framingham, USA | Moderate | n= 1,352 (males= 6,634, females= 718) Age= 54 (9) | Memory and executive function | | Diabetes: - (memory, executive function) |
| Derby et al. | 2021 | Longitudinal | SWAN, USA | Moderate | N = 1139  Age = 53.4 (2.6) | Memory and executive function | | - Diabetes and FBG (Memory, executive function) |
| Dintica et al. | 2022 | Cross sectional | CARDIA, USA | Moderate | MetS = 534  No MetS = 2346 | Memory, executive function, processing speed, verbal fluency, global cognition | | MetS: - (Memory, executive function, processing speed, verbal fluency) |
| Dixon et al. | 2021 | Longitudinal | SWAN, USA | Moderate | Total = 1,953 (European American = 1,000, African American = 516, Asian American = 437)  Age: European American = 45.9 **± 2.7**, African American = 45.9 ± 2.6, Asian American = 46.1 ± 2.6 | Executive function, working and episodic memory | | 0 Diabetes (working memory)  - Diabetes (executive function, episodic memory) |
| Fava et al. | 2013 | Prospective longitudinal | Italy | Low | Total: n = 96 (Group A = 48, Group B = 48)  Age: Group A = 53 (7), Group B = 54.6 (8.1) | Memory, executive function, global cognition | | - HbA_1_c (Memory) |
| Fuh et al. | 2007 | Matched, case–control study from a population-based cohort | KIWI; Kinmen, Taiwan | Low | Normal (N = 144) Impaired glucose tolerance (N = 68) Diabetes mellitus (N = 72)  Age:  Normal = 47.9 (4.3) Impaired glucose tolerance = 46.8 (4.1) Diabetes mellitus = 47.9 (4.3) | Memory, attention, and executive function | | - Diabetes (Attention) |
| Gerber et al. | 2021 | Multicentre population-based cohort | CARDIA; USA | Low | Total: n = 2809  No NAFLD >51 HU: n=2136  Mild NAFLD >40–51 HU: n=392  Severe NAFLD ≤40 HU: n=281  Age:  Total = 50.1 (3.6)  No NAFLD >51 HU = 50 (3.7)  Mild NAFLD >40–51 HU = 50.3 (3.6)  Severe NAFLD ≤40 HU = 50.5 (3.6) | Memory, executive function, psychomotor speed, and global cognition | | - Diabetes (Memory, executive function, global cognition) |
| Gonzalez et al. | 2018 | Prospective, epidemiologic | ARIC, USA | Moderate | n= 13,720 (males= 5,873, females= 7,397) Age= 54.1 (5.7) | Memory, executive function, and global cognition | | FBG: - (global cognition) |
| Houle et al. | 2019 | Cross sectional, longitudinal | HAALSI, South Africa | Moderate | n= 2,059 (males= 2,345, females= 2,714) Age= 40-59 | Memory, executive function, attention, global cognition, temporal orientation | | Diabetes: - (global cognition) |
| Ihle-Hansen et al. | 2019 | Prospective | ACE, Norway | High | n= 3,413 (males= 1,774, females= 1,639)  Age= 63.9 (0.7) [males= 63.9 (0.7), females= 63.9 (0.6)] | Global cognition | | Diabetes: - (global cognition) |
| Kaffashian et al. | 2013 | Prospective | Whitehall II, UK | High | n= 4,374 (males= 3,162, females= 1,212)  Age= 55.2 (5.1) | Memory, executive function, attention, global cognition, and inductive reasoning | | Diabetes: - (global cognition) |
| Kaffashian et al. | 2013 | Longitudinal, | Whitehall II, UK | High | n= 7,830 (males= 4,153, females= 3,677)  Age= 55.6 (6) | Memory, executive function, attention, global cognition, inductive reasoning | | Diabetes: - (global cognition) |
| Kazlauskaite et al. | 2020 | Longitudinal | SWAN, USA | Moderate | n= 2,149 (all females)  Age= 50.7 (2.9) | Memory, psychomotor speed | | MeTS: - (psychomotor speed) |
| Knopman et al. | 2001 | Longitudinal | ARIC, USA | Low | n= 10,882 (males= 6,978, females= 3,904) Age= 56.8 (5.7) | Memory, executive function | | Diabetes: - (memory, executive function) |
| Knopman et al. | 2018 | Longitudinal | ARIC, USA | Low | n= 10,882 (males= 8,723, females= 7,137) Age= 51.4 (4.9) | Memory, executive function, global cognition | | Diabetes: - (global cognition) |
| Knopman et al. | 2009 | Longitudinal | ARIC, USA | Moderate | n= 1,130 (males= 429, females= 701) Age= 59 (4.3) | Memory, executive function | | Diabetes: - (memory, executive function)  FBG: - (memory, executive function)  MeTS: - (memory, executive function) |
| Kohde et al. | 2012 | Cross sectional, case‑control | India | Moderate | n= 120 (60 patients vs. 60 controls) Age: patients= 53.7 (6.9) vs. controls= 52.1 (6.2) | Attention | | MeTS: - (attention) |
| Kumar et al. | 2008 | Cross-sectional study | PATH Through Life Project; Australia | Moderate | Diabetics: N = 428; Non-diabetics: N = 465  Age:  Diabetics = 62.62 (1.16)  Non-diabetics = 62.55 (1.48) | Memory, attention, global cognition, and psychomotor speed | | - Diabetes (Global cognition, fine motor speed, psychomotor speed) |
| Kumari et al., | 2005 | Longitudinal | Whitehall II, UK | Moderate | NGT: males= 3,407, females= 1,334; IGT: males= 405, females= 192; Diabetes: males= 208, females= 101  Age: NGT: males= 55.1, females= 55.7; IGT: males= 58.2, females= 57.8; Diabetes: males= 57.9, females= 58.9 | Memory, inductive reasoning, executive function | | Diabetes: - (inductive reasoning, executive function) |
| Leong et al. | 2020 | Prospective, longitudinal | TILDA, Ireland | Moderate | Non hypertensive= 2,280 (males= 848, females= 1,432); Hypertensive w/o medication= 2,823 (males= 1,420, females= 1,403); Hypertensive with medication= 3,070 (males= 1,495, females= 1,595) Age: Non hypertensive= 59.5; Hypertensive w/o medication= 62.7; Hypertensive with medication= 68.1 | Global cognition | | Diabetes: - (global cognition) |
| Ma et al. | 2020 | Cross-sectional | Ganzhou, China | Moderate | Total = 56 (T2DM = 27, without T2DM = 29) | Visual search ability, Working memory, Proprioception | | - Diabetes (Visual search ability, Working memory, Proprioception) |
| Muhkerjee et al. | 2022 | Cross-sectional | Kolkata; India | Moderate | Total: n = 72 (diabetic retinopathy = 36, W/O diabetic retinopathy = 36)  Age:  Diabetic retinopathy = 55.2 (5.9)  W/O Diabetic retinopathy = 54.3 (6.8) | Memory, attention, executive function, global cognition | | - Diabetes, HbA1c (Global cognition) |
| Palacios-Mendoza et al. | 2018 | Cross sectional | Guayaquil, Ecuador | High | Diabetes= 142 (males= 65, females= 76); No Diabetes= 167 (males= 116, females= 50) Age: Diabetes= 59.9 (4.2); No Diabetes= 59.9 (3.8) | Memory, executive function, intelligence, attention | | Diabetes: - (memory, attention, processing speed, and executive function)  HbA_1_c: - (memory, attention, executive function) |
| Pan et al. | 2018 | Longitudinal | CHARLS, China | Low | n= 1,825  Age= 56.9 (8) | Memory, global cognition | | Diabetes: - (memory, global cognition) |
| Panigrahi et al. | 2021 | Cross sectional | New Delhi, India | Moderate | n= 80 (males= 31, females= 49)  Age= 51.7 (7.2) | Global cognition | | Diabetes: - (global cognition)  HbA_1_c: - (global cognition)  FBG: - (global cognition) |
| Passos et al. | 2021 | Cross-sectional | Pró-Saúde study, Rio de Janeiro, Brazil | Moderate | Total = 488 (male = 235, female = 253)  Age Group:  45-54: n = 243  55-64: n = 145 | Memory, executive function | | - Diabetes (Memory, executive function) |
| Rawlings et al., | 2014 | Prospective | ARIC, USA | Moderate | n= 13,351  Age= 48-67 | Memory, executive function, global cognition | | Diabetes: - (memory, executive function, global cognition, processing speed)  HbA_1_c: - (memory, executive function, global cognition) |
| Szczesnia et al. | 2020 | Longitudinal | PURE, Poland | High | n= 547 (males= 195, females= 352) Age= 56.2 (6.5) [males= 55.1 (6.8), females= 56.9 (6.3)] | Attention, executive function, psychomotor speed, global cognition | | Diabetes: - (psychomotor speed, executive function, global cognition) |
| Szoeke et al. | 2016 | Longitudinal, prospective | WHAP, Australia | Moderate | Baseline  n= 387  Age= 49.6 (2.5) | Memory | | FBG: - (memory) |
| Tipnis et al. | 2022 | Prospective, case control | Maharashtra, India | Moderate | Diabetes: n = 150  No Diabetes: n = 150 | Global Cognition | | Diabetes: - (Global cognition) |
| Tufvesson et al. | 2013 | Prospective | MDCS, Sweden | High | n= 933 (males= 369, females= 564) Age= 57.5 (5.7) | Global cognition | | Diabetes: - (processing speed) |
| Udayakuma et al. | 2018 | Cross-sectional | Chennai, Tamil Nadu, India | Low | Total: 100 (male)  Age = 50-60 years | Memory, executive function | | - Diabetes (Memory, executive function) |
| Veugen et al. | 2018 | Observational, prospective | Maastricht, Netherlands | High | n= 3,011 (males= 1,542, females = 1,469  Age= 52 (5) | Memory, executive function, attention | | FBG: - (memory, executive function, attention, processing speed) |
| Wang et al. | 2016 | Cross sectional | APAC, China | High | n= 3,048 (males= 1,727, females= 1,321) Age= 57.9 (11.1) | Global cognition | | Diabetes: - (global cognition) |
| Wei et al., | 2018 | Cross sectional | CHARLS, China | High | n= 6,732 | Memory, global cognition | | Diabetes: - (memory, executive function) |
| Winkler et al. | 2014 | Population based | RECALL, Germany | Moderate | n= 1,089 (males= 515, females= 574)  Age= 58.4 (4.1) | Memory, executive function, visuospatial orientation | | Diabetes: - (memory, executive function) |
| Wu et al. | 2022 | Cross sectional | CHARLS, China | Moderate | Diabetes Free: n = 7036; Treated Diabetes: n = 250; Untreated Diabetes: n = 628  Age: Diabetes Free:51.75 (4.81); Treated Diabetes: 53.35 (4.35); Untreated Diabetes: 52.05 (4.64) | Episodic memory, global cognition, mental intactness | | Diabetes: - (episodic memory, global cognition, mental intactness) |
| Yaffe et al. | 2014 | Longitudinal, prospective | CARDIA, USA | Moderate | Year 25 examination  n= 3,381 (males= 1,475, females= 1,906)  Age = 50.2 (3.6) | Memory, executive function, psychomotor speed | | FBG: - (memory, executive function, attention) |
| Yang et al. | 2018 | Prospective | MACS, USA | Moderate | n= 900 (all males) | Psychomotor speed, attention, executive function, memory | | Diabetes: - (memory, executive function, attention, psychomotor speed)  FBG: - (executive function, attention, psychomotor speed)  HbA_1_c: - (memory, attention, executive function) |
| Yulug et al. | 2020 | Prospective | Istanbul Medipol University; Turkey | Low | T2DM = 15  Prediabetes = 16  New-onset diabetes = 15  Age = 50.68 ± 8.43 | Global cognition, orientation, memory, executive function | | - Diabetes (Memory) |
| Zhang et al. | 2019 | Cross sectional | CHARLS, China | Low | No Diabetes= 7,151; Controlled Diabetes= 232; Untreated Diabetes= 185; Treated Diabetes= 241 Age: No Diabetes= 59.5 (9.5) | Memory, executive function, global cognition | | Diabetes, FBG and HbA1c: - (memory, executive function) |

0 = No relationship; - = negative relationship; + = Positive relationship

**Abbreviations:** *ACE*, Akershus Cardiac Examination; *APAC,* Asymptomatic Polyvascular Abnormalities Community; *ARIC, Atherosclerosis Risk in Communities*; *ASCEND*, A Study of Cardiovascular Events in Diabetes; *Barcelona-AsIA*, Asymptomatic Intracranial Atherosclerosis; *BHS*, Bogalusa Heart Study; *BIP,* Bezafibrate Infarction Prevention; *BP,* blood pressure; *CARDIA,* Coronary Artery Risk Development in Young Adults; *CHARLS*, China Health and Retirement Longitudinal Study; *DBP*, diastolic blood pressure; *ELSA*, English Longitudinal Study of Ageing; ELSA, Brazilian Longitudinal Study of Adult Health *HAALSI*, Health and Aging in Africa; *HANDLS, healthy Aging in Neighborhoods of Diversity Across the Life Span*; *HHP*, Honolulu Heart Program; *KALS,* Kaohsiung Atherosclerosis Longitudinal Study ; *KEEPSCog*, Kronos Early Estrogen Prevention cognitive; *KIHD,* Kuopio Ischaemic Heart Disease Risk Factor Study; *MACS,* Multicentre AIDS Cohort Study; *MADT*, Middle-Aged Danish Twins; *MDCS,* Malmö Diet and Cancer Study; *MORGEN*, Monitoring Project on Cardiovascular Disease Risk Factors; *MRC,* Medical Research Council; *NSHD,* National Survey of Health and Development; *PATH,* Population Assessment of Tobacco and Health*; PURE,* prospective Urban and Rural Epidemiological; *RECALL*, Risk Factors, Evaluation of Coronary Calcium and Lifestyle; *SBP*, systolic blood pressure; *Swan,* Study of Women’s Health Across the Nation; TILDA, The Irish Longitudinal Study on Ageing; *VETSA,* Vietnam Era Twin Study of Aging and; *WHAP,* Women’s Health Aging Project.

***Supplemental Table B.2.*** Summary of studies with no relationship between diabetes, associated metrics, and cognitive measures at midlife.

| Author | Year | Study design | Setting | Study quality | Participants | Cognitive Variables | Relationship |
| --- | --- | --- | --- | --- | --- | --- | --- |
| Albanese et al. | 2012 | Longitudinal | UK | Moderate | n= 2,083 (males= 1,013, females= 1,070)  Age= 43-53 | Memory, attention, executive function, global cognition | HbA_1_c: 0 |
| Aliberti et al | 2020 | Cross sectional | ELSA, Brazil | High | n= 5,275 Age= 56.3 | Memory, executive function, temporal orientation, global cognition | Diabetes: 0 |
| Alves de Moraes | 2002 | Longitudinal | ARIC, USA | Low | n= 8,058  Age= 56.7 (5.6) | Memory, executive function | Diabetes and FBG: 0 |
| Babaei et al. | 2013 | RCT | Iran | Low | n= 52 (28 patients vs. 24 controls) Age= 57.1 (5.9) | Memory | Diabetes and FBG: 0 |
| Bancks et al. | 2017 | Retrospective, longitudinal | ARIC, USA | High | Visit 2  n= 10,133 (males= 4,458, females= 5,674)  Age = 56.6 (5.7) | Memory, executive function | Diabetes: 0 |
| Bangen et al. | 2013 | Cross sectional, longitudinal | Framingham, USA | High | n= 1,436 (males= 660, females= 775) Age= 54 (9) | Memory, executive function, global cognition, visuospatial organisation | FBG: 0 |
| Blodgett et al. | 2020 | Longitudinal follow-up | MRC NSHD, UK | Moderate | Total: n = 3,111 (male = 1,550, female = 1,561)  Age 53 (Total) = 2,897 (Male = 1421, Female = 1476)  Age 60-64 (Total) = 2,203 (Male = 1,055, Female = 1,148)  Age 69 (Total) = 2,116 (1,037, Female = 1,079) | Verbal Memory | DM: 0 |
| Boots et al. | 2015 | Cross sectional | WRAP, USA | High | n= 315 (males= 102, females= 213) Age= 58.6 (6.3) | Memory, executive function, visuospatial organisation, global cognition | Diabetes and FBG: 0 |
| Bressler et al. | 2013 | Prospective | ARIC, USA | Low | White: n= 8,364 (males= 3,859, females= 4,505); African American: n= 2,083 (males= 716, females= 1,367) Age: White= 57 (5.6); African American= 55.8 (5.7) | Memory, executive function | Diabetes and FBG: 0 |
| Brunner et al. | 2017 | Cross sectional, longitudinal | Whitehall II, UK | Low | Visit 5 and 6  n= 175  Age= (55-64 | Global cognition | Diabetes and FBG: 0 |
| Carmichael et al. | 2019 | Observational, longitudinal | BHS; USA | Low | Total: n = 50 (male = 26, female = 14)  Age = 48.8 (8.7) | Memory, executive function | FBG: 0 |
| Chen et al. | 2018 | Longitudinal | ARIC-NCS, USA | High | n= 12,515 (males= 5,334, females= 6,981) Age= 56.9 (5.7) | Memory, executive function | Diabetes and FBG: 0 |
| Christman et al. | 2011 | Prospective | ARIC, USA | Moderate | n= 8,958 (males= 3,943, females= 5,015)  Age= 56.5 (5.6) | Memory, executive function | FBG: 0 |
| Chuang et al. | 2023 | Retrospective | Taiwan | Moderate | n = 618 (males = 298, females = 320)  Age: males = 53.4 (5.7), females = 52.9 (5.7) | Global Cognition | FBG: 0 |
| Cohen-Manheim et al. | 2016 | Cross sectional | Jerusalem, Israel | High | n= 507 (males= 343, females= 164) Age= 49.9 (0.8) | Memory, executive function, attention | FBG: 0 |
| Creavin et al. | 2012 | Prospective, longitudinal | Caerphilly, Wales | Moderate | Phase 1  n= 2,512  Age= 52.8 | Attention, global cognition, inductive reasoning | FBG: 0 |
| Cui et al. | 2016 | Case-control | Guangzhou, China | Moderate | Hypertensive: n= 278; Controls= 155 Age: Hypertensive= 54.2 (4.2); Controls= 55.8 (5.5) | Intelligence, global cognition | FBG: 0 |
| de Menezes et al. | 2021 | Longitudinal | ELSA, Brazil | High | n= 7,063  Age= 58.9 (5.9) | Memory, executive function, global cognition | Diabetes and FBG: 0 |
| Dearborn-Tomazos et al. | 2019 | Longitudinal observational | ARIC, USA | Low | n= 13,588 (males= 3,000, females= 7,588) Age= 54.6 (5.7) | Memory, executive function, global cognition | Diabetes and FBG: 0 |
| Debette et al. | 2011 | Prospective | Framingham, USA | Moderate | n= 1,352 (males= 6,634, females= 718) Age= 54 (9) | Memory, executive function | FBG: 0 |
| Del Vecchio et al. | 2023 | Retrospective, Case Control | Rome, Italy | Moderate | n = 112 (males = 81, females = 31)  Control Group: n = 31 (males = 20, females = 11)  Study Group: n = 81 (males = 61, females = 41) | Global Cognition | Diabetes: 0 |
| Dixon et al. | 2021 | Longitudinal | SWAN, USA | Moderate | Total = 1,953 (European American = 1,000, African American = 516, Asian American = 437)  Age: European American = 45.9 **± 2.7**, African American = 45.9 ± 2.6, Asian American = 46.1 ± 2.6 | Executive function, working and episodic memory | 0 Diabetes (working memory) |
| Elbaz et al. | 2014 | Longitudinal | Whitehall II, UK | High | n= 4,699 (males= 3,324, females= 1,375) Age= 48.6 (5.8) | Inductive reasoning | Diabetes and FBG: 0 |
| Elkins et al. | 2005 | Prospective | ARIC, USA | High | n= 12,096 (males= 12,039, females= 57) Age= 57 (5.7) | Memory, executive function | Diabetes and FBG: 0 |
| Fava et al. | 2013 | Prospective longitudinal | Italy | Low | Total: n = 96 (Group A = 48, Group B = 48)  Age: Group A = 53 (7), Group B = 54.6 (8.1) | Memory, executive function, global cognition | 0 FBG, MeTS |
| Ferguson et al. | 2018 | Cross sectional | CARDIA, USA | Moderate | n= 634 (males= 305, females=329)  Age= 50.4 (3.5) | Memory, executive function | Diabetes and FBG: 0 |
| Ford et al. | 2010 | Longitudinal | SWAN, USA | Moderate | n= 2,003 Age= 50 (2.6) | Memory, psychomotor speed | Diabetes and FBG: 0 |
| Giugliano et al. | 2018 | RCT | Pozzilli, Italy | Low | Active Treatment: n= 18; Control: n=18  Age: Active Treatment= 58.2 (8); Control= 57.9 (6.7) | Executive function, global cognition | FBG: 0 |
| Gonzalez et al. | 2018 | Prospective, epidemiologic | ARIC, USA | Moderate | n= 13,720 (males= 5,873, females= 7,397) Age= 54.1 (5.7) | Memory, executive function, global cognition | Diabetes: 0 |
| Gottesman et al. | 2017 | Prospective | ARIC, USA | High | n= 15,744 (males= 7,054, females= 8,690) Age= 54.2 (5.8) | Memory, executive function, global cognition | Diabetes and FBG: 0 |
| Gottesman et al. | 2014 | Prospective | ARIC, USA | High | Normal BP= 5,101 (males= 2,195, females= 2,908)  Pre- HT= 2,875 (males= 1,388, females= 1,487)  HT= 5,500 (males= 2,401, females= 3,099) Age: Normal BP= 55(7), Pre-hypertensive= 56 (8), Hypertensives 57 (8) | Memory, executive function, global cognition | Diabetes and FBG: 0 |
| Gourley et al. | 2020 | Cross sectional | Texas, USA | Moderate | n= 132 (males= 59, females= 73) Age= 49 (6) | Memory, executive function, intelligence, attention | Diabetes: 0 |
| Gwizdala et al. | 2023 | Cross sectional | Texas, USA | Moderate | n = 1292 (males = 530, females = 762)  Age: 48.2 (5.24) | Memory, executive function, global cognition | FBG: 0 |
| Hajjar et al. | 2018 | Observational | USA | Low | n= 511 (males = 163, females = 348)  Age= 49.1 (0.5) | Memory, attention, executive function, global cognition, visuospatial ability | Diabetes: 0 |
| Hajjar et al. | 2016 | Longitudinal | USA | Moderate | n= 291 (males= 191, females= 400)  Age= 48.8 (0.4) | Memory, executive function, attention, global cognition, visuospatial organisation | Diabetes: 0 |
| Hakamada-Taguchi et al. | 2002 | Observational | Tokyo, Japan | Low | n= 26 (all females)  Age = 57.7 (1.1) | Memory, attention, executive function, intelligence | FBG: 0 |
| Haley et al. | 2010 | Cross sectional | USA | Moderate | n= 38 Age= 50 (6.4) | Global cognition, intelligence, memory, attention, executive function, psychomotor speed | FBG and MeTS: 0 |
| Hawkins et al. | 2018 | Cross sectional | USA | Low | n= 67 (males = 19, females = 48)  Age= 55.8 (5.5) [males= 55.8 (5), females= 55.8 (5.8)] | Memory, attention, executive function, global cognition, processing speed | HbA_1_c: 0 |
| Hoffmann et al. | 2021 | Longitudinal | Recall, Germany | High | Normal BP: n= 692 (males= 242, females= 450); Incident Hypertension T1: n= 366 (males= 175, females= 191); Incident Hypertension T2: n= 245 (males= 109, females= 136); Temporary Hypertension: n= 329 (males= 183, females= 209); Prevalent Hypertension: n= 1,145 (males= 635, females= 510).  Age: Normal BP= 55.2 (6.6); Incident Hypertension T1= 57.8 (7.1); Incident Hypertension T2: 56.5 (6.6); Temporary Hypertension= 57.6 (7.1); Prevalent Hypertension= 60.2 (7.1) | Memory, executive function, visuospatial organisation | Diabetes and FBG: 0 |
| Hossain et al. | 2020 | Cross sectional, longitudinal | HANDLS, USA | High | n= 128 (males= 102, females= 126)  Age: males= 57.1 (0.5), females= 56 (0.8) | Memory, attention, executive function, global cognition | Diabetes: 0 |
| Houle et al. | 2019 | Cross sectional, longitudinal | HAALSI, South Africa | Moderate | n= 2,059 (males= 2,345, females= 2,714) Age= 40- 59 | Memory, executive function, attention, global cognition, temporal orientation | FBG: 0 |
| Ihle-Hansen et al. | 2019 | Prospective | ACE, Norway | High | n= 3,413 (males= 1,774, females= 1,639)  Age= 63.9 (0.7) [males= 63.9 (0.7), females= 63.9 (0.6)] | Global cognition | FBG and HbA_1_c: 0 |
| Jia et al. | 2021 | Cross-sectional | China | Moderate | Total = 4,923 (male = 2,162, female = 2,761)  Age:  55-64 years = 2,042  65-74 years = 2,004  75+ years = 877 | Global Cognition | DM: 0 |
| John et al. | 2021 | Longitudinal cohort | NCDS; UK | High | N = 3730  Age = 44 | Memory, and executive function | 0 HbA1c |
| Kaffashian et al. | 2013 | Prospective | Whitehall II, UK | High | n= 4,374 (males= 3,162, females= 1,212)  Age= 55.2 (5.1) | Memory, executive function, attention, global cognition, inductive reasoning | FBG: 0 |
| Kaffashian et al. | 2011 | Prospective | Whitehall II, UK | High | n= 4,827 (males= 3,486, females= 1,341) Age: males= 55.1 (5.9), females= 55.3 (5.9) | Memory, executive function, attention, global cognition, inductive reasoning | Diabetes and FBG: 0 |
| Kalmijn et al. | 2002 | Prospective | MORGEN, The Netherlands | Moderate | n= 1,927 (males= 905, females= 989)  Age: males= 56.6 (7.1), females= 56.2 (7.1) | memory, executive function, global cognition, psychomotor speed | Diabetes and FBG: 0 |
| Kazlauskaite et al. | 2020 | Longitudinal | SWAN, USA | Moderate | n= 2,149 (all females); No MetS= 1,514, MetS= 635)  Age= 50.7 (2.9); No MetS= 50.6 (2.8), MetS= 51.1 (3.2) | Memory, psychomotor speed | FBG: 0 |
| Knopman et al. | 2001 | Longitudinal | ARIC, USA | Low | n= 10,882 (males= 6,978, females= 3,904) Age= 56.8 (5.7) | Memory, executive function | FBG: 0 |
| Knopman et al. | 2018 | Longitudinal | ARIC, USA | Low | n= 10,882 (males= 8,723, females= 7,137) Age= 51.4 (4.9) | Memory, executive function, global cognition | FBG: 0 |
| Kohde et al. | 2012 | Cross sectional, case‑control | India | Moderate | n= 120 (60 patients vs. 60 controls) Age: patients= 53.7 (6.9), controls= 52.1 (6.2) | Attention | Diabetes and FBG: 0 |
| Kovacs et al. | 2014 | Cross sectional | Hungary | Moderate | Hypertensive= 72; controls= 85  Age: Hypertensive= 43.6; controls= 43.6 | Memory, executive function, attention, psychomotor speed, visuospatial organisation | FBG: 0 |
| Kumar et al., | 2020 | Longitudinal | ASCEND, UK | Low | n= 80 Age= 59 | Global cognition, attention, executive function, memory | Diabetes: 0 |
| Kumari et al. | 2005 | Longitudinal | Whitehall II, UK | Moderate | n: NGT: males= 3,407, females= 1,334; IGT: males= 405, females= 192; Diabetes: males= 208, females= 101  Age: NGT: males= 55.1, females= 55.7; IGT: males= 58.2, females= 57.8; Diabetes: males= 57.9, females= 58.9 | Memory, inductive reasoning, executive function | FBG: 0 |
| Launer et al., | 2015 | Cross sectional | CARDIA, USA | Low | n= 680 Age= 50.3 (3.5) | Memory, executive function | Diabetes and HbA_1_c: 0 |
| Lin et al. | 2020 | Longitudinal | KALS, Taiwan | High | n= 528 Age= 53.9 (8.4) | Global cognition, memory, executive function, visuospatial orientation, attention | Diabetes and FBG: 0 |
| Liu et al. | 2022 | Prospective | Neck-Shoulder and Lumbocrural Pain Hospital and the Affiliated Hospital of Shandong University of TCM; China | Moderate | Overall: n = 156; Controls = 64, SCI = 92  Age: Controls = 57.1 (6.3); SCI = 57.6 (6.7) | General Cognition | 0 Diabetes and FBG |
| Lopez-Oloriz et al. | 2014 | Population-based | AsIA, Spain | Low | n= 95 Age= 59.9 (3.3) | Executive function, psychomotor speed, global cognition | Diabetes: 0 |
| Lutski et al. | 2019 | Longitudinal | BIP, Israel | High | n= 337  Age= 56.6 (6.4) | Memory, executive function, attention, global cognition, visuospatial organisation | Diabetes and FBG: 0 |
| Mahinrad et al. | 2020 | Longitudinal | CARDIA, USA | Moderate | n= 191 (males= 104, females= 87)  Age= 56 (4) | Memory, executive function, attention | Diabetes and FBG: 0 |
| Masi et al. | 2018 | Prospective | MRC NSHD, UK | Moderate | n= 1,249 (males= 578, females= 671)  Age= 60–64 | Memory, attention, executive function | Diabetes and HbA_1_c: 0 |
| Mattei et al. | 2019 | Longitudinal, observational | Boston Puerto Rican Health Study, USA | Moderate | n = 1,499 (Without T2DM= 711 [male = 188, female = 523], With T2DM = 465 [male = 128, female = 337], Controlled T2DM= 118 [male = 36, female = 82], Uncontrolled T2DM= 339 [male = 90, female = 249])  Age: Without T2DM= 56.0 ± 7.7, With T2DM = 58.9 ± 7.2, Controlled T2DM= 58.2 ± 6.8, Uncontrolled T2DM= 59.1 ± 7.3 | Memory, attention, executive function, global cognition | Diabetes and FBG: 0 |
| Mefford et al. | 2021 | Multicentre longitudinal, prospective | CARDIA study, USA | Moderate | N = 3328  Time-averaged LDL-C levels over follow-up, mg/dL: <100 (n = 519) 100–129 (n = 1,094) 130–159 (n = 961) ≥160 (n = 754)  Age:  Time-averaged LDL-C levels over follow-up, mg/dL: <100 = 46.9 (3.2); 100–129 = 49.2 (3.5); 130–159 = 51.1 (3.1); ≥160 = 52.6 (2.5) | Memory, attention, and executive function | 0 Diabetes |
| Meyer et al. | 2020 | Cross-sectional analysis of longitudinal | CARDIA study; USA | Moderate | Total = 597 (male = 267, female = 330)  Age = 55.2 ± 3.5 | Memory, executive function, global cognition, | DM: 0 |
| Nunley et al. | 2017 | Prospective, observational | Pittsburgh Epidemiology of Diabetes Complications Study; USA | High | N = 108  Age = 49.52 (7.04) | Memory, attention, executive function, global cognition, intelligence and psychomotor speed | 0 Diabetes, HbA_1_c |
| Olaya et al. | 2017 | Longitudinal | ELSA, UK | High | n= 5,523 Age= 50-64 | Memory | Diabetes: 0 |
| Olaya et al. | 2019 | Longitudinal | ELSA, UK | High | n= 4,372 (males= 2,023, females= 2,349) Age= 56.8 (4.1) | Memory | Diabetes: 0 |
| Palacios-Mendoza et al. | 2018 | Cross sectional | Guayaquil, Ecuador | High | Diabetes: n= 142 (males= 65, females= 76); No Diabetes: n= 167 (males= 116, females= 50) Age: Diabetes= 59.9 (4.2); No Diabetes= 59.9 (3.8) | Memory, executive function, intelligence, attention | FBG: 0 |
| Palta et al. | 2019 | Prospective | ARIC, USA | Moderate | No PA: n= 1996 (males= 795, females= 1,201); Low: n= 774 (males= 247, females= 497); Middle: n= 669 (males= 295, females= 404); High: n=1,194 (males= 733, females= 461) Age: No PA= 59.1 (5.4); Low= 59.4 (5.6) Middle= 60.6 (5.9); High= 60.2 (5.8) | Memory, executive function | Diabetes and FBG: 0 |
| Patel et al. | 2019 | Prospective, observational, interventional | New Civil Hospital Surat, India | Moderate | Total: n = 60 (male = 34, female = 26)  Age = 45.88 ± 1.70 | Memory, attention, verbal fluency, language, and visuospatial abilities | + Insulin on DM (Memory, attention, verbal fluency, language, and visuospatial abilities) |
| Pokharel et al., | 2019 | Prospective | ARIC, USA | Moderate | n= 18,222  Age= ? | Memory, executive function | Diabetes and FBG: 0 |
| Power et al., | 2017 | Prospective | ARIC, USA | High | n= 15,792 Age= 57.5 (5.7) | Memory, executive function | Diabetes and FBG: 0 |
| Ravona-springer et al. | 2020 | Prospective longitudinal | Israel Registry for Alzheimer Prevention (IRAP) study; Israel | Moderate | Total: N = 483; FH+ = 379, FH- = 104  Age: FH+ = 54.55 (6.76), FH- = 56.42 (6.19) | Memory, executive function, and global cognition | 0 Diabetes, FBG, HbA_1_c |
| Rawlings et al., | 2014 | Prospective | ARIC, USA | Moderate | n= 13,351  Age= 48-67 | Memory, executive function, global cognition | FBG: 0 |
| Razavi et al. | 2020 | Epidemiological | Bogalusa Heart, USA | High | n= 960  Age= 48.4 (5.1) | Memory, attention, executive function, processing speed | Diabetes and FBG: 0 |
| Reis et al. | 2013 | Cross-sectional | CARDIA study; USA | Moderate | Total: N = 2510; Coronary artery calcified plaque: Present = 686, Absent = 1824; Abdominal aortic calcified plaque: Present = 1297, Absent = 1213  Age:  Coronary artery calcified plaque: Present = 51.1 (3.3), Absent = 49.6 (3.7); Abdominal aortic calcified plaque: Present = 50.6 (3.6), Absent = 49.5 (3.7) | Memory, attention, and executive function | 0 Diabetes, HbA_1_c |
| Richards et al. | 2005 | Longitudinal | MRC NSHD, UK | Low | n= 1,764  Age= 43-53 | Memory, executive function | Diabetes: 0 |
| Ritchie et al. | 2017 | Cross sectional | PREVENT, UK | Low | Non-FH: n= 107 (males= 35, females= 71); FH: n= 103 (males= 29, females= 73) Age: Non-FH= 52.7; FH= 53.3 | Memory, executive function, visuospatial organisation, attention | Diabetes: 0 |
| Root et al. | 2015 | Prospective, epidemiological | ARIC, USA | Moderate | n= 10,041  Age= 53.5 | Memory, executive function | Diabetes and FBG: 0 |
| Rose et al. | 2010 | Prospective, epidemiologic | ARIC, USA | Low | OH No= 12,050; OH Yes= 652 Age: OH No= 53.9; OH Yes= 57.3 | Memory, executive function | Diabetes and FBG: 0 |
| Sadahiro et al. | 2019 | Cross-sectional | Japan | Low | Diabetes = 117  No diabetes = 1,127  Age:  Diabetes = 73.5 ± 5.0  No diabetes = 73.0 ± 5.5 | Global cognition | 0 Diabetes |
| Salzwedel et al. | 2019 | Prospective, observational | Germany | High | n= 401 (Males= 321, Females= 80) Age= 54.5 (6.3) | Global cognition | Diabetes: 0 |
| Sierra et al. | 2004 | Cross sectional | Barcelona, Spain | High | Without WML: n= 37 (males= 24, females= 13); With WML: n= 23 (males= 14, females= 9) Age: Without WML= 53.9 (3.5); With WML= 55.2 (4.2) | Intelligence, memory, attention | FBG: 0 |
| Singh-Manoux et al. | 2003 | Longitudinal | Whitehall II, UK | Moderate | n= 10,308 (males = 6,896, females= 3,411)  Age = 44.5 | Memory, executive function, inductive reasoning | FBG: 0 |
| Singh-Manoux et al. | 2005 | Cross sectional, longitudinal | Whitehall II, UK | Moderate | n= 5,838  Age: males= 43.9 (5.9), females= 44.4 (6) | Memory, executive function, inductive reasoning | Diabetes: 0 |
| Singh-Manoux et al. | 2009 | Cross-sectional, longitudinal | Whitehall II, UK | Moderate | n= 5,292 (males= 3,810, females= 1,481)  Age: CHD= 59.4 (5.5); No CHD= 55.2 (5.9) | Memory, executive function, inductive reasoning | Diabetes and FBG: 0 |
| Solomon et al. | 2009 | Prospective | CAIDE, Finland | Low | Low TC: n= 123 (males= 40, females= 83);  Intermediate TC: n= 365 (males= 151, females= 216;  High TC: n= 894 (males= 332, females= 566)  Age: Low TC= 49.1; Intermediate TC= 48.8; High TC= 50.4 | Memory, executive function, global cognition | Diabetes: 0 |
| Sun et al. | 2020 | Prospective | CARDIA, USA | Moderate | Year 25 examination  n= 1,369 (males= 630, females= 739)  Age= 50.8 (3.3) | Memory, executive function, psychomotor speed | Diabetes: 0 |
| Suvila et al. | 2021 | Prospective | CARDIA, USA | High | n= 2,496 (males= 534, females= 1,689) Age= 55.1 (3.6) | Memory, executive function, psychomotor speed, global cognition | Diabetes: 0 |
| Szczesnia et al. | 2020 | Longitudinal | PURE, Poland | High | n= 547 (males= 195, females= 352) Age= 56.2 (6.5) [males= 55.1 (6.8), females = 56.9 (6.3)] | Attention, executive function, psychomotor speed, global cognition | FBG: 0 |
| Tufvesson et al. | 2013 | Prospective | MDCS, Sweden | High | n= 933 (males= 369, females= 564) Age= 57.5 (5.7) | Global cognition | FBG: 0 |
| Tuligenga et al. | 2014 | Prospective, longitudinal | Whitehall II study; UK | Moderate | Total: N = 5653; Normoglycaemia (n=4703); Prediabetes (n=648); Newly diagnosed diabetes (n=115); Known diabetes (n=187)  Age: Total = 54.4; Normoglycaemia = 55.1 (5.9); Prediabetes = 57.5 (6.1); Newly diagnosed diabetes 59.0 (6.1); Known diabetes = 57.4 (6.3) | Memory, executive function, and inductive reasoning |  |
| Veugen et al. | 2018 | Observational, prospective | Maastricht, Netherlands | High | n= 3,011 (males= 1,542, females = 1,469  Age= 52 (5) | Memory, executive function, attention | Diabetes and HbA1c: 0 |
| Walker et al. | 2019 | Prospective | ARIC, USA | High | n= 3,012 (males= 1,382, females= 1,630)  Age= 55.5 (5.4) | Memory, executive function, psychomotor speed | Diabetes: 0 |
| Walker et al. | 2019 | Prospective | ARIC, USA | High | n= 3,012 (males= 1,382, females= 1,630)  Age= 55.5 (5.4) | Memory, executive function, psychomotor speed | FBG: 0 |
| Wang et al. | 2016 | Cross sectional | APAC, China | High | n= 3,048 (males= 1,727, females= 1,321) Age= 57.9 (11.1) | Global cognition | FBG: 0 |
| Wang et al. | 2018 | Prospective epidemiological | ARIC, USA | High | n= 13,720  Age= 55.5 (5.4) | Memory, executive function, global cognition | Diabetes and FBG: 0 |
| Wharton et al. | 2014 | Cross sectional | KEEPSCog, USA | High | n= 571 (all females)  Age= 42–59 | Memory, attention, executive function, global cognition | FBG: 0 |
| Whitaker et al. | 2021 | Longitudinal | CARDIA, USA | Moderate | n= 1,970 (males = 822, females = 1,148)  Age = 45.3 (3.6) | Memory, executive function | Diabetes: 0 |
| Wieczorek et al. | 2016 | Prospective | Poland | Moderate | n= 74 (males= 44, females= 30) Age= 59 (50–63) | Global cognition | Diabetes: 0 |
| Winkler et al. | 2014 | Population based | RECALL, Germany | Moderate | n= 1,089 (males= 515, females= 574)  Age= 58.4 (4.1) | Memory, executive function, visuospatial orientation | FBG and HbA1c: 0 |
| Yaffe et al. | 2014 | Longitudinal, prospective | CARDIA, USA | Moderate | Year 25 Examination  n= 3,381 (males= 1,475, females = 1,906)  Age= 50.2 (3.6) | Memory, executive function, psychomotor speed | Diabetes and FBG: 0 |
| Yano et al. | 2018 | Prospective | MACS, USA | High | n= 900 (all males)  Age= 54 | Memory, attention, executive function, psychomotor speed | Diabetes and FBG: 0 |
| Ylilauri et al. | 2017 | Prospective | KIHD, Finland | High | n= 2,497 (all males)  Age= 42–60 | Global cognition, attention  executive function, memory | Diabetes and FBG: 0 |
| Young et al. | 2006 | Longitudinal, observational | ARIC, USA | Moderate | n= 7,148 (males= 3,173, females= 3,975) Age= 53.7 | Memory, executive function | Diabetes and FBG: 0 |
| ZekiAlHazzouri et al. | 2015 | Prospective | CARDIA, USA | Moderate | n= 2618 (males= 1,125, females= 1,493) Age= 45.3 (3.6) | Memory, executive function | Diabetes and FBG: 0 |

0, no association

**Abbreviations:** *ACE*, Akershus Cardiac Examination; *APAC,* Asymptomatic Polyvascular Abnormalities Community; *ARIC, Atherosclerosis Risk in Communities*; *ASCEND*, A Study of Cardiovascular Events in Diabetes; *Barcelona-AsIA*, Asymptomatic Intracranial Atherosclerosis; *BHS*, Bogalusa Heart Study; *BIP,* Bezafibrate Infarction Prevention; *BP,* blood pressure; *CARDIA,* Coronary Artery Risk Development in Young Adults; *CHARLS*, China Health and Retirement Longitudinal Study; *DBP*, diastolic blood pressure; *ELSA*, English Longitudinal Study of Ageing; ELSA, Brazilian Longitudinal Study of Adult Health *HAALSI*, Health and Aging in Africa; *HANDLS, healthy Aging in Neighborhoods of Diversity Across the Life Span*; *HHP*, Honolulu Heart Program; *KALS,* Kaohsiung Atherosclerosis Longitudinal Study ; *KEEPSCog*, Kronos Early Estrogen Prevention cognitive; *KIHD,* Kuopio Ischaemic Heart Disease Risk Factor Study; *MACS,* Multicentre AIDS Cohort Study; *MADT*, Middle-Aged Danish Twins; *MDCS,* Malmö Diet and Cancer Study; *MORGEN*, Monitoring Project on Cardiovascular Disease Risk Factors; *MRC,* Medical Research Council; *NSHD,* National Survey of Health and Development; *PATH,* Population Assessment of Tobacco and Health*; PURE,* prospective Urban and Rural Epidemiological; *RECALL*, Risk Factors, Evaluation of Coronary Calcium and Lifestyle; *SBP*, systolic blood pressure; *Swan,* Study of Women’s Health Across the Nation; TILDA, The Irish Longitudinal Study on Ageing; *VETSA,* Vietnam Era Twin Study of Aging and; *WHAP,* Women’s Health Aging Project.

***Supplemental Table B.3.*** Summary of negative relationships between diabetes metrics and cognition at midlife by study design and quality.

| Study Design | Memory | Attention | Executive Function | Global Cognition | Psychomotor Speed | Inductive Reasoning | Intelligence | Visuospatial Organisation | Temporal Orientation |
| --- | --- | --- | --- | --- | --- | --- | --- | --- | --- |
| Diabetes |  |  |  |  |  |  |  |  |  |
| Individual Study Cohorts | (Bayes-Marin et al., 2020, Palacios-Mendoza et al., 2018, Winkler et al., 2014, Debette et al., 2011, Derby et al., 2021, Fuh et al., 2007, Ma et al., 2020, Passos et al., 2021, Udayakumar and Muthupandian, 2018, Yang et al., 2018, Yulug et al., 2020, Wu et al., 2022) | (Palacios-Mendoza et al., 2018, Fuh et al., 2007, Yang et al., 2018) | (Palacios-Mendoza et al., 2018, Tufvesson et al., 2013, Winkler et al., 2014, Debette et al., 2011, Derby et al., 2021, Fuh et al., 2007, Ma et al., 2020, Passos et al., 2021, Udayakumar and Muthupandian, 2018, Yang et al., 2018) | (Houle et al., 2019, Ihle-Hansen et al., 2019, Leong et al., 2020, Panigrahi and Chaudhury, 2021, Wang et al., 2016, Almani et al., 2021, Mukherjee et al., 2022, Del Vecchio et al., 2023, Wu et al., 2022) | (Yang et al., 2018) | (Creavin et al., 2012) | - | **-** | - |
| Longitudinal Study Cohorts | (Anstey et al., 2014, Bangen et al., 2013, Zhang et al., 2019, Carmichael et al., 2019) | (Anstey et al., 2014, Bangen et al., 2013) | (Anstey et al., 2014, Zhang et al., 2019, Carmichael et al., 2019) | (Anstey et al., 2014) | - | - | - | (Bangen et al., 2013) | - |
| Study Quality (n=) | Low: 5  Moderate: 7  High: 3 | Low: 1  Moderate: 2  High: 2 | Low: 4  Moderate: 7  High: 2 | Low: 1  Moderate: 8  High: 2 | Low: -  Moderate: 1  High: - | Low: -  Moderate: 1  High: - | Low: -  Moderate: -  High: - | Low: **-**  Moderat**e**: **-**  High: 1 | Low: -  Moderate: -  High: - |
| FBG |  |  |  |  |  |  |  |  |  |
| Individual Study Cohorts | (Backestrom et al., 2015, Veugen et al., 2018, Derby et al., 2021, Fava et al., 2013, Fuh et al., 2007) | (Veugen et al., 2018, Fuh et al., 2007, Yang et al., 2018) | (Veugen et al., 2018, Derby et al., 2021, Fava et al., 2013, Fuh et al., 2007, Yang et al., 2018) | (Gonzalez et al., 2018, Panigrahi and Chaudhury, 2021, Fava et al., 2013, Tipnis et al., 2022) | (Yang et al., 2018) | - | - | **-** | - |
| Longitudinal Study Cohorts | (Zhang et al., 2019, Szoeke et al., 2016) | - | (Zhang et al., 2019) | - | - | - | - | **-** | - |
| Study Quality (n=) | Low: 3  Moderate: 3  High: 1 | Low: 1  Moderate: 1  High: 1 | Low: 3  Moderate: 2  High: 1 | Low: 1  Moderate: 3  High: - | Low: -  Moderate: 1  High: - | Low: -  Moderate: -  High: - | Low: -  Moderate: -  High: - | Low: -  Moderate: -  High: - | Low: -  Moderate: -  High: - |
| HbA1c |  |  |  |  |  |  |  |  |  |
| Individual Study Cohorts | (Palacios-Mendoza et al., 2018, Fava et al., 2013, Yang et al., 2018) | (Palacios-Mendoza et al., 2018, Yang et al., 2018) | (Palacios-Mendoza et al., 2018, Fava et al., 2013, Yang et al., 2018, Otsuka et al., 2019) | (Panigrahi and Chaudhury, 2021, Almani et al., 2021, Fava et al., 2013, Mukherjee et al., 2022) | - | - | - | - | - |
| Longitudinal Study Cohorts | (Zhang et al., 2019) | - | (Zhang et al., 2019) | - | - | - | - | - | - |
| Study Quality (n=) | Low: 2  Moderate: 1  High: 1 | Low: 0  Moderate: 1  High: 1 | Low: 2  Moderate: 1  High: 2 | Low: 2  Moderate: 2  High: 0 | Low: -  Moderate: -  High: - | Low: -  Moderate: -  High: - | Low: -  Moderate: -  High: - | Low: -  Moderate: -  High: - | Low: -  Moderate: -  High: - |
| MeTS |  |  |  |  |  |  |  |  |  |
| Individual Study Cohorts | (Babaei et al., 2013, Fava et al., 2013) | (Khode et al., 2012) | (Fava et al., 2013) | (Creavin et al., 2012, Fava et al., 2013) | - | (Creavin et al., 2012) | - | - | - |
| Longitudinal Study Cohorts | (Dintica et al., 2022) | - | (Dintica et al., 2022) | - | (Kazlauskaite et al., 2020) | - | - | - | - |
| Study Quality (n=) | Low: 2  Moderate: 1  High: - | Low: -  Moderate: 1  High: - | Low: 1  Moderate: 1  High: - | Low: 1  Moderate: 1  High: - | Low: -  Moderate: 1  High: - | Low: -  Moderate: 1  High: - | Low: -  Moderate: -  High: - | Low: -  Moderate: -  High: - | Low: -  Moderate: -  High: - |

***Supplemental Table B.4.*** Critical appraisal of included studies using the AXIS tool.

| **Question** | Albanese et al. | Aliberti et al., | Almani et al. | Alves de Moraes et al. | Anstey et al. | Babaei et al. | Backestrom et al. | Bancks et al. | Bangen et al. | Bayes-Marin et al. | Blodgett et al. | Boots et al. | Bressler et al. | Britton et al. |
| --- | --- | --- | --- | --- | --- | --- | --- | --- | --- | --- | --- | --- | --- | --- |
| **Introduction** | | | | | | | | | | | | | | |
| Question 1 | **Y** | **Y** | **Y** | **N** | **Y** | **Y** | **Y** | **Y** | **Y** | **Y** | **Y** | **Y** | **N** | **Y** |
| **Methods** | | | | | | | | | | | | | | |
| Question 2 | **Y** | **Y** | **Y** | **Y** | **Y** | **Y** | **Y** | **Y** | **Y** | **Y** | **Y** | **Y** | **Y** | **Y** |
| Question 3 | **N** | **N** | **Y** | **N** | **N** | **N** | **N** | **N** | **N** | **N** | **N** | **N** | **N** | **N** |
| Question 4 | **Y** | **Y** | **Y** | **Y** | **Y** | **Y** | **Y** | **Y** | **Y** | **Y** | **Y** | **Y** | **Y** | **Y** |
| Question 5 | **Y** | **Y** | **Y** | **Y** | **Y** | **Y** | **Y** | **Y** | **Y** | **Y** | **Y** | **Y** | **Y** | **Y** |
| Question 6 | **Y** | **Y** | **Y** | **Y** | **Y** | **U** | **Y** | **Y** | **Y** | **Y** | **Y** | **Y** | **Y** | **Y** |
| Question 7 | **N** | **Y** | **N** | **N** | **Y** | **U** | **N** | **N** | **Y** | **N** | **Y** | **N** | **N** | **N** |
| Question 8 | **Y** | **Y** | **Y** | **Y** | **Y** | **Y** | **Y** | **Y** | **Y** | **Y** | **Y** | **Y** | **Y** | **Y** |
| Question 9 | **Y** | **Y** | **Y** | **Y** | **Y** | **Y** | **Y** | **Y** | **Y** | **Y** | **Y** | **Y** | **Y** | **Y** |
| Question 10 | **U** | **Y** | **Y** | **Y** | **Y** | **Y** | **Y** | **Y** | **Y** | **Y** | **Y** | **Y** | **Y** | **N** |
| Question 11 | **Y** | **Y** | **Y** | **Y** | **Y** | **N** | **Y** | **Y** | **Y** | **Y** | **Y** | **Y** | **Y** | **N** |
| **Results** | | | | | | | | | | | | | | |
| Question 12 | **Y** | **Y** | **Y** | **Y** | **Y** | **N** | **Y** | **Y** | **Y** | **Y** | **Y** | **Y** | **Y** | **Y** |
| Question 13 | **U** | **N** | **N** | **N** | **N** | **U** | **N** | **N** | **N** | **N** | **N** | **N** | **N** | **N** |
| Question 14 | **N** | **U** | **Y** | **Y** | **Y** | **U** | **N** | **N** | **Y** | **N** | **N** | **N** | **N** | **N** |
| Question 15 | **Y** | **Y** | **Y** | **Y** | **Y** | **Y** | **Y** | **Y** | **Y** | **Y** | **Y** | **Y** | **Y** | **Y** |
| Question 16 | **Y** | **Y** | **N** | **Y** | **Y** | **Y** | **Y** | **Y** | **Y** | **Y** | **Y** | **Y** | **Y** | **Y** |
| **Discussion** | | | | | | | | | | | | | | |
| Question 17 | **Y** | **Y** | **Y** | **Y** | **Y** | **N** | **Y** | **Y** | **Y** | **Y** | **Y** | **Y** | **Y** | **Y** |
| Question 18 | **N** | **Y** | **N** | **Y** | **Y** | **N** | **Y** | **Y** | **Y** | **Y** | **Y** | **Y** | **N** | **Y** |
| **Other** | | | | | | | | | | | | | | |
| Question 19 | **N** | **N** | **N** | **U** | **N** | **N** | **N** | **N** | **N** | **N** | **N** | **N** | **N** | **U** |
| Question 20 | **Y** | **Y** | **Y** | **U** | **Y** | **Y** | **Y** | **Y** | **U** | **Y** | **Y** | **Y** | **Y** | **Y** |

| **Question** | Brunner et al. | Carmichael et al. | Cerhan et al. | Chen et al. | Chen et al. | Cherbuin et al. | Chosy et al. | Christman et al. | Chuang et al. | Cohen-Manheim et al. | Creavin et al. | Cui et al. | de Menezes et al. | Dearborn et al. | Dearborn-Tomazos et al. |  |
| --- | --- | --- | --- | --- | --- | --- | --- | --- | --- | --- | --- | --- | --- | --- | --- | --- |
| **Introduction** | | | | | | | | | | | | | | | | |
| Question 1 | **U** | **Y** | **Y** | **Y** | **Y** | **Y** | **Y** | **Y** | **Y** | **Y** | **Y** | **Y** | **Y** | **Y** | **Y** |  |
| **Methods** | | | | | | | | | | | | | | | | |
| Question 2 | **U** | **Y** | **Y** | **Y** | **Y** | **Y** | **Y** | **Y** | **Y** | **Y** | **Y** | **Y** | **Y** | **Y** | **Y** |  |
| Question 3 | **U** | **N** | **Y** | **N** | **N** | **N** | **N** | **N** | **N** | **Y** | **N** | **N** | **N** | **N** | **N** |  |
| Question 4 | **Y** | **Y** | **Y** | **Y** | **Y** | **Y** | **Y** | **Y** | **Y** | **Y** | **Y** | **Y** | **Y** | **Y** | **Y** |  |
| Question 5 | **Y** | **Y** | **Y** | **Y** | **Y** | **Y** | **Y** | **Y** | **Y** | **Y** | **Y** | **Y** | **Y** | **Y** | **Y** |  |
| Question 6 | **Y** | **Y** | **Y** | **Y** | **Y** | **Y** | **Y** | **Y** | **Y** | **Y** | **Y** | **Y** | **Y** | **Y** | **Y** |  |
| Question 7 | **N** | **N** | **N** | **N** | **Y** | **N** | **N** | **N** | **N** | **N** | **N** | **N** | **N** | **N** | **N** |  |
| Question 8 | **Y** | **Y** | **Y** | **Y** | **Y** | **Y** | **Y** | **Y** | **Y** | **Y** | **Y** | **Y** | **Y** | **Y** | **Y** |  |
| Question 9 | **Y** | **Y** | **Y** | **Y** | **Y** | **Y** | **Y** | **Y** | **Y** | **Y** | **Y** | **Y** | **Y** | **Y** | **Y** |  |
| Question 10 | **U** | **N** | **N** | **Y** | **Y** | **Y** | **N** | **Y** | **Y** | **Y** | **N** | **Y** | **Y** | **Y** | **Y** |  |
| Question 11 | **Y** | **Y** | **Y** | **Y** | **Y** | **Y** | **Y** | **Y** | **Y** | **Y** | **Y** | **Y** | **Y** | **Y** | **N** |  |
| **Results** | | | | | | | | | | | | | | | | |
| Question 12 | **Y** | **Y** | **Y** | **Y** | **Y** | **Y** | **Y** | **Y** | **Y** | **Y** | **Y** | **Y** | **Y** | **Y** | **N** |  |
| Question 13 | **N** | **N** | **N** | **N** | **N** | **N** | **N** | **N** | **N** | **N** | **N** | **N** | **N** | **N** | **N** |  |
| Question 14 | **N** | **N** | **N** | **N** | **N** | **N** | **N** | **N** | **N** | **N** | **N** | **N** | **N** | **N** | **N** |  |
| Question 15 | **Y** | **Y** | **Y** | **Y** | **Y** | **Y** | **Y** | **Y** | **Y** | **Y** | **Y** | **Y** | **Y** | **Y** | **Y** |  |
| Question 16 | **Y** | **Y** | **Y** | **Y** | **Y** | **Y** | **Y** | **Y** | **Y** | **Y** | **Y** | **Y** | **Y** | **Y** | **Y** |  |
| **Discussion** | | | | | | | | | | | | | | | | |
| Question 17 | **Y** | **Y** | **Y** | **Y** | **Y** | **Y** | **Y** | **Y** | **Y** | **Y** | **Y** | **Y** | **Y** | **Y** | **Y** |  |
| Question 18 | **N** | **Y** | **Y** | **Y** | **Y** | **Y** | **U** | **Y** | **Y** | **N** | **Y** | **N** | **Y** | **Y** | **Y** |  |
| **Other** | | | | | | | | | | | | | | | | |
| Question 19 | **N** | **N** | **N** | **N** | **N** | **N** | **N** | **N** | **N** | **N** | **N** | **N** | **N** | **N** | **N** |  |
| Question 20 | **N** | **Y** | **Y** | **Y** | **Y** | **Y** | **Y** | **U** | **Y** | **Y** | **Y** | **Y** | **Y** | **Y** | **Y** |  |

| **Question** | Debette et al. | Del Vecchio et al. | Derby et al. | Dintica et al. | Dixon et al. | Elbaz et al. | Elkins et al. | Elmassry et al. | Fava et al. | Ferguson et al. | Ford et al. | Fuh et al. | Gerasimenko et al. | Gerber et al. | Giugliano et al. | Gonzalez et al. |
| --- | --- | --- | --- | --- | --- | --- | --- | --- | --- | --- | --- | --- | --- | --- | --- | --- |
| Question 1 | **Y** | **Y** | **Y** | **Y** | **Y** | **Y** | **Y** | **Y** | **Y** | **Y** | **Y** | **Y** | **Y** | **N** | **N** | **Y** |
| Question 2 | **Y** | **Y** | **Y** | **Y** | **Y** | **Y** | **Y** | **Y** | **Y** | **Y** | **Y** | **Y** | **Y** | **N** | **N** | **Y** |
| Question 3 | **N** | **N** | **Y** | **N** | **N** | **N** | **N** | **N** | **N** | **N** | **N** | **N** | **N** | **N** | **N** | **N** |
| Question 4 | **Y** | **Y** | **N** | **Y** | **Y** | **Y** | **Y** | **Y** | **Y** | **Y** | **Y** | **N** | **Y** | **Y** | **Y** | **Y** |
| Question 5 | **Y** | **Y** | **Y** | **Y** | **Y** | **Y** | **Y** | **Y** | **Y** | **Y** | **Y** | **Y** | **U** | **Y** | **U** | **Y** |
| Question 6 | **Y** | **Y** | **Y** | **Y** | **Y** | **Y** | **Y** | **Y** | **Y** | **Y** | **Y** | **Y** | **U** | **Y** | **Y** | **Y** |
| Question 7 | **N** | **N** | **N** | **N** | **N** | **N** | **N** | **N** | **N** | **N** | **N** | **N** | **N** | **Y** | **N** | **N** |
| Question 8 | **Y** | **Y** | **Y** | **Y** | **Y** | **Y** | **Y** | **Y** | **Y** | **Y** | **Y** | **Y** | **Y** | **Y** | **Y** | **Y** |
| Question 9 | **Y** | **Y** | **Y** | **Y** | **Y** | **Y** | **Y** | **Y** | **Y** | **Y** | **Y** | **Y** | **Y** | **Y** | **Y** | **Y** |
| Question 10 | **N** | **Y** | **N** | **Y** | **Y** | **Y** | **Y** | **Y** | **Y** | **N** | **N** | **Y** | **N** | **Y** | **Y** | **Y** |
| Question 11 | **Y** | **Y** | **Y** | **Y** | **Y** | **Y** | **Y** | **Y** | **Y** | **Y** | **Y** | **Y** | **N** | **Y** | **Y** | **Y** |
| Question 12 | **Y** | **Y** | **Y** | **Y** | **Y** | **Y** | **Y** | **Y** | **Y** | **Y** | **Y** | **Y** | **N** | **Y** | **Y** | **Y** |
| Question 13 | **N** | **N** | **N** | **N** | **N** | **N** | **N** | **N** | **N** | **N** | **N** | **N** | **N** | **N** | **N** | **N** |
| Question 14 | **N** | **N** | **N** | **N** | **N** | **N** | **N** | **N** | **N** | **N** | **N** | **N** | **N** | **N** | **N** | **N** |
| Question 15 | **Y** | **Y** | **Y** | **Y** | **Y** | **Y** | **Y** | **Y** | **Y** | **Y** | **Y** | **Y** | **Y** | **Y** | **Y** | **Y** |
| Question 16 | **Y** | **Y** | **Y** | **Y** | **Y** | **Y** | **Y** | **Y** | **Y** | **Y** | **Y** | **Y** | **Y** | **Y** | **Y** | **Y** |
| Question 17 | **Y** | **Y** | **Y** | **Y** | **Y** | **Y** | **Y** | **Y** | **Y** | **Y** | **Y** | **Y** | **Y** | **Y** | **Y** | **Y** |
| Question 18 | **Y** | **Y** | **Y** | **Y** | **Y** | **Y** | **Y** | **N** | **Y** | **Y** | **Y** | **N** | **N** | **N** | **N** | **Y** |
| Question 19 | **N** | **N** | **N** | **N** | **N** | **N** | **N** | **N** | **N** | **N** | **N** | **N** | **U** | **N** | **N** | **N** |
| Question 20 | **U** | **Y** | **Y** | **Y** | **Y** | **Y** | **Y** | **Y** | **N** | **Y** | **Y** | **Y** | **N** | **Y** | **Y** | **U** |

| **Question** | Gottesman et al. | Gottesman et al. | Gourley et al. | Gupta et al. | Gwizdala et al. | Hajjar et al. | Hajjar et al. | Hakamada-Taguchi et al. | Haley et al. | Hawkins et al. | Henriksen et al. | Hitesh et al. | Hoffmann et al. | Hossain et al. | Houle et al. | Ihle-Hansen et al. |
| --- | --- | --- | --- | --- | --- | --- | --- | --- | --- | --- | --- | --- | --- | --- | --- | --- |
| **Introduction** | | | | | | | | | | | | | | | | |
| Question 1 | **Y** | **Y** | **Y** | **Y** | **Y** | **Y** | **Y** | **Y** | **Y** | **Y** | **Y** | **Y** | **Y** | **Y** | **Y** | **Y** |
| **Methods** | | | | | | | | | | | | | | | | |
| Question 2 | **Y** | **Y** | **Y** | **Y** | **Y** | **Y** | **Y** | **Y** | **Y** | **Y** | **Y** | **Y** | **Y** | **Y** | **Y** | **Y** |
| Question 3 | **N** | **N** | **N** | **N** | **N** | **N** | **N** | **N** | **N** | **N** | **N** | **N** | **N** | **N** | **N** | **N** |
| Question 4 | **Y** | **Y** | **Y** | **Y** | **Y** | **Y** | **Y** | **Y** | **Y** | **Y** | **Y** | **Y** | **Y** | **Y** | **Y** | **Y** |
| Question 5 | **Y** | **Y** | **Y** | **Y** | **Y** | **Y** | **Y** | **Y** | **Y** | **N** | **Y** | **Y** | **Y** | **Y** | **Y** | **Y** |
| Question 6 | **Y** | **Y** | **Y** | **Y** | **Y** | **Y** | **U** | **U** | **Y** | **N** | **Y** | **Y** | **Y** | **Y** | **Y** | **Y** |
| Question 7 | **N** | **N** | **N** | **N** | **N** | **N** | **N** | **N** | **N** | **N** | **N** | **N** | **N** | **N** | **N** | **N** |
| Question 8 | **Y** | **Y** | **Y** | **Y** | **Y** | **Y** | **Y** | **Y** | **Y** | **Y** | **Y** | **Y** | **Y** | **Y** | **Y** | **Y** |
| Question 9 | **Y** | **Y** | **Y** | **Y** | **Y** | **Y** | **U** | **Y** | **Y** | **Y** | **Y** | **Y** | **Y** | **Y** | **Y** | **Y** |
| Question 10 | **Y** | **Y** | **N** | **N** | **N** | **N** | **N** | **N** | **N** | **N** | **Y** | **Y** | **Y** | **Y** | **N** | **Y** |
| Question 11 | **Y** | **Y** | **Y** | **Y** | **Y** | **Y** | **N** | **N** | **Y** | **N** | **Y** | **Y** | **Y** | **Y** | **Y** | **Y** |
| **Results** | | | | | | | | | | | | | | | | |
| Question 12 | **Y** | **Y** | **Y** | **Y** | **Y** | **Y** | **Y** | **Y** | **Y** | **N** | **Y** | **Y** | **Y** | **Y** | **Y** | **Y** |
| Question 13 | **N** | **N** | **N** | **N** | **N** | **N** | **N** | **N** | **N** | **N** | **N** | **N** | **N** | **N** | **N** | **N** |
| Question 14 | **N** | **N** | **N** | **N** | **N** | **N** | **N** | **N** | **N** | **N** | **N** | **N** | **N** | **N** | **N** | **N** |
| Question 15 | **Y** | **Y** | **Y** | **Y** | **Y** | **Y** | **Y** | **Y** | **Y** | **Y** | **Y** | **Y** | **Y** | **Y** | **Y** | **Y** |
| Question 16 | **Y** | **Y** | **Y** | **Y** | **Y** | **Y** | **Y** | **Y** | **Y** | **U** | **Y** | **Y** | **Y** | **Y** | **Y** | **Y** |
| **Discussion** | | | | | | | | | | | | | | | | |
| Question 17 | **Y** | **Y** | **Y** | **Y** | **Y** | **Y** | **Y** | **Y** | **Y** | **U** | **Y** | **Y** | **Y** | **Y** | **Y** | **Y** |
| Question 18 | **Y** | **Y** | **Y** | **Y** | **Y** | **Y** | **Y** | **Y** | **Y** | **Y** | **Y** | **Y** | **Y** | **Y** | **Y** | **Y** |
| **Other** | | | | | | | | | | | | | | | | |
| Question 19 | **N** | **N** | **N** | **N** | **N** | **N** | **N** | **N** | **N** | **N** | **N** | **N** | **N** | **N** | **N** | **N** |
| Question 20 | **Y** | **Y** | **Y** | **Y** | **Y** | **Y** | **Y** | **Y** | **Y** | **Y** | **Y** |  | **Y** | **Y** | **Y** | **Y** |

| **Question** | Janseen et al. | Jia et al. | John et al. | Kaffashian et al. | Kaffashian et al. | Kaffashian et al. | Kalmijn et al. | Kazlauskaite et al. | Knopman et al. | Knopman et al.7 | Knopman et al. | Kohde et al. | Kovacs et al. | Kumar et al. | Kumar et al. |  |
| --- | --- | --- | --- | --- | --- | --- | --- | --- | --- | --- | --- | --- | --- | --- | --- | --- |
| **Introduction** | | | | | | | | | | | | | | | | |
| Question 1 | **Y** | **Y** | **Y** | **Y** | **Y** | **Y** | **Y** | **Y** | **Y** | **Y** | **Y** | **Y** | **Y** | **Y** | **Y** |  |
| **Methods** | | | | | | | | | | | | | | | |  |
| Question 2 | **Y** | **Y** | **Y** | **Y** | **Y** | **Y** | **Y** | **Y** | **Y** | **Y** | **Y** | **Y** | **Y** | **Y** | **Y** |  |
| Question 3 | **N** | **N** | **Y** | **N** | **N** | **N** | **N** | **N** | **N** | **N** | **N** | **Y** | **N** | **N** | **N** |  |
| Question 4 | **Y** | **Y** | **Y** | **Y** | **Y** | **Y** | **Y** | **Y** | **Y** | **Y** | **Y** | **Y** | **Y** | **Y** | **Y** |  |
| Question 5 | **Y** | **Y** | **Y** | **Y** | **Y** | **Y** | **Y** | **Y** | **Y** | **Y** | **Y** | **Y** | **Y** | **Y** | **Y** |  |
| Question 6 | **Y** | **Y** | **Y** | **Y** | **Y** | **Y** | **Y** | **Y** | **Y** | **Y** | **Y** | **U** | **Y** | **Y** | **Y** |  |
| Question 7 | **N** | **N** | **Y** | **N** | **N** | **N** | **N** | **N** | **N** | **N** | **N** | **N** | **N** | **N** | **N** |  |
| Question 8 | **Y** | **Y** | **Y** | **Y** | **Y** | **Y** | **Y** | **Y** | **Y** | **Y** | **Y** | **Y** | **Y** | **Y** | **Y** |  |
| Question 9 | **Y** | **Y** | **Y** | **Y** | **Y** | **Y** | **Y** | **Y** | **Y** | **Y** | **Y** | **Y** | **Y** | **Y** | **Y** |  |
| Question 10 | **Y** | **Y** | **N** | **Y** | **Y** | **Y** | **N** | **N** | **N** | **N** | **N** | **Y** | **Y** | **N** | **Y** |  |
| Question 11 | **Y** | **Y** | **Y** | **Y** | **Y** | **Y** | **Y** | **Y** | **Y** | **Y** | **Y** | **Y** | **Y** | **Y** | **Y** |  |
| **Results** | | | | | | | | | | | | | | | |  |
| Question 12 | **Y** | **Y** | **Y** | **Y** | **Y** | **Y** | **Y** | **Y** | **Y** | **N** | **Y** | **Y** | **Y** | **Y** | **Y** |  |
| Question 13 | **N** | **N** | **N** | **N** | **N** | **N** | **N** | **N** | **N** | **N** | **N** | **N** | **N** | **N** | **N** |  |
| Question 14 | **N** | **N** | **N** | **N** | **N** | **N** | **N** | **N** | **N** | **N** | **N** | **N** | **N** | **N** | **N** |  |
| Question 15 | **Y** | **Y** | **Y** | **Y** | **Y** | **Y** | **N** | **Y** | **Y** | **Y** | **Y** | **Y** | **Y** | **Y** | **Y** |  |
| Question 16 | **Y** | **Y** | **Y** | **Y** | **Y** | **Y** | **Y** | **Y** | **Y** | **Y** | **Y** | **Y** | **Y** | **Y** | **Y** |  |
| **Discussion** | | | | | | | | | | | | | | | |  |
| Question 17 | **Y** | **Y** | **Y** | **Y** | **Y** | **Y** | **Y** | **Y** | **Y** | **Y** | **Y** | **Y** | **Y** | **Y** | **Y** |  |
| Question 18 | **Y** | **Y** | **Y** | **Y** | **Y** | **Y** | **Y** | **Y** | **Y** | **N** | **Y** | **Y** | **N** | **N** | **Y** |  |
| **Other** | | | | | | | | | | | | | | | |  |
| Question 19 | **N** | **N** | **N** | **N** | **N** | **N** | **N** | **N** | **N** | **N** | **N** | **N** | **N** | **N** | **N** |  |
| Question 20 | **Y** | **Y** | **Y** | **Y** | **Y** | **Y** | **Y** | **Y** | **Y** | **Y** | **N** | **Y** | **Y** | **N** | **Y** |  |

| **Question** | Kumari et al. | Launer et al. | Launer et al. | Leong et al. | Li et al. | Lin et al. | Liu et al. | Lopez-Oloriz et al. | Lutski et al. | Ma et al. | Mahinrad et al. | Masi et al. | Masi et al. | Mattei et al. | Mefford et al. | Meyer et al. |
| --- | --- | --- | --- | --- | --- | --- | --- | --- | --- | --- | --- | --- | --- | --- | --- | --- |
| **Introduction** | | | | | | | | | | | | | | | | |
| Question 1 | **Y** | **Y** | **Y** | **Y** | **Y** | **Y** | **Y** | **Y** | **Y** | **Y** | **Y** | **Y** | **Y** | **Y** | **Y** | **Y** |
| **Methods** | | | | | | | | | | | | | | | | |
| Question 2 | **Y** | **Y** | **Y** | **Y** | **Y** | **Y** | **Y** | **Y** | **Y** | **Y** | **Y** | **Y** | **Y** | **Y** | **Y** | **Y** |
| Question 3 | **N** | **N** | **N** | **N** | **N** | **N** | **N** | **N** | **N** | **N** | **N** | **N** | **N** | **N** | **N** | **N** |
| Question 4 | **Y** | **Y** | **Y** | **Y** | **Y** | **Y** | **Y** | **Y** | **Y** | **Y** | **Y** | **Y** | **Y** | **Y** | **Y** | **Y** |
| Question 5 | **Y** | **Y** | **Y** | **Y** | **Y** | **Y** | **Y** | **Y** | **Y** | **Y** | **Y** | **Y** | **U** | **Y** | **Y** | **Y** |
| Question 6 | **Y** | **Y** | **Y** | **Y** | **Y** | **Y** | **Y** | **Y** | **Y** | **Y** | **Y** | **Y** | **U** | **Y** | **Y** | **Y** |
| Question 7 | **N** | **N** | **N** | **N** | **N** | **Y** | **N** | **N** | **Y** | **N** | **N** | **N** | **N** | **N** | **N** | **N** |
| Question 8 | **Y** | **Y** | **Y** | **Y** | **Y** | **Y** | **Y** | **Y** | **Y** | **Y** | **Y** | **Y** | **Y** | **Y** | **Y** | **Y** |
| Question 9 | **Y** | **Y** | **Y** | **Y** | **Y** | **Y** | **Y** | **Y** | **Y** | **Y** | **Y** | **Y** | **Y** | **Y** | **Y** | **Y** |
| Question 10 | **Y** | **N** | **N** | **Y** | **Y** | **Y** | **Y** | **N** | **N** | **N** | **Y** | **N** | **N** | **Y** | **Y** | **Y** |
| Question 11 | **Y** | **Y** | **Y** | **Y** | **Y** | **Y** | **Y** | **N** | **Y** | **Y** | **Y** | **Y** | **N** | **Y** | **Y** | **Y** |
| **Results** | | | | | | | | | | | | | | | | |
| Question 12 | **Y** | **N** | **N** | **N** | **Y** | **Y** | **Y** | **Y** | **Y** | **Y** | **Y** | **Y** | **N** | **Y** | **Y** | **Y** |
| Question 13 | **N** | **N** | **N** | **N** | **N** | **N** | **N** | **N** | **N** | **N** | **N** | **N** | **N** | **N** | **N** | **N** |
| Question 14 | **N** | **N** | **N** | **N** | **N** | **N** | **N** | **N** | **Y** | **N** | **N** | **N** | **N** | **N** | **N** | **N** |
| Question 15 | **Y** | **Y** | **Y** | **Y** | **Y** | **Y** | **Y** | **Y** | **Y** | **Y** | **Y** | **Y** | **U** | **Y** | **Y** | **Y** |
| Question 16 | **Y** | **Y** | **Y** | **Y** | **Y** | **Y** | **Y** | **Y** | **Y** | **Y** | **Y** | **Y** | **Y** | **Y** | **Y** | **Y** |
| **Discussion** | | | | | | | | | | | | | | | | |
| Question 17 | **Y** | **Y** | **Y** | **Y** | **Y** | **Y** | **Y** | **Y** | **Y** | **Y** | **Y** | **Y** | **U** | **Y** | **Y** | **Y** |
| Question 18 | **Y** | **N** | **N** | **Y** | **Y** | **Y** | **Y** | **Y** | **Y** | **Y** | **Y** | **Y** | **N** | **N** | **Y** | **Y** |
| **Other** | | | | | | | | | | | | | | | | |
| Question 19 | **N** | **N** | **N** | **N** | **N** | **N** | **N** | **N** | **N** | **N** | **N** | **N** | **U** | **N** | **N** | **N** |
| Question 20 | **N** | **Y** | **Y** | **Y** | **Y** | **Y** | **Y** | **Y** | **Y** | **Y** | **Y** | **Y** | **N** | **Y** | **Y** | **Y** |

| **Question** | Moore et al. | Muhkerjee et al. | Nunley et al. | Olaya et al. | Olaya et al. | Otsuka et al. | Palacios-Mendoza et al. | Palta et al. | Pan et al. | Panigrahi et al. | Passos et al. | Patel et al. | Pokharel et al. | Power et al | Ravona-Springer et al. |
| --- | --- | --- | --- | --- | --- | --- | --- | --- | --- | --- | --- | --- | --- | --- | --- |
| **Introduction** | | | | | | | | | | | | | | | |
| Question 1 | **Y** | **Y** | **Y** | **Y** | **Y** | **Y** | **Y** | **Y** | **Y** | **Y** | **Y** | **Y** | **Y** | **Y** | **Y** |
| **Methods** | | | | | | | | | | | | | | | |
| Question 2 | **Y** | **Y** | **Y** | **Y** | **Y** | **Y** | **Y** | **Y** | **Y** | **Y** | **Y** | **Y** | **Y** | **Y** | **Y** |
| Question 3 | **N** | **Y** | **N** | **N** | **N** | **Y** | **N** | **N** | **N** | **N** | **N** | **N** | **N** | **N** | **N** |
| Question 4 | **Y** | **Y** | **Y** | **Y** | **Y** | **Y** | **Y** | **Y** | **Y** | **Y** | **Y** | **Y** | **Y** | **Y** | **Y** |
| Question 5 | **Y** | **Y** | **Y** | **Y** | **Y** | **Y** | **Y** | **Y** | **Y** | **Y** | **Y** | **Y** | **Y** | **Y** | **Y** |
| Question 6 | **Y** | **Y** | **Y** | **Y** | **Y** | **Y** | **Y** | **Y** | **Y** | **Y** | **Y** | **Y** | **Y** | **Y** | **Y** |
| Question 7 | **N** | **N** | **Y** | **N** | **N** | **N** | **N** | **N** | **N** | **N** | **N** | **N** | **N** | **N** | **N** |
| Question 8 | **Y** | **Y** | **Y** | **Y** | **Y** | **Y** | **Y** | **Y** | **Y** | **Y** | **Y** | **Y** | **Y** | **Y** | **Y** |
| Question 9 | **Y** | **Y** | **Y** | **Y** | **Y** | **Y** | **Y** | **Y** | **Y** | **Y** | **Y** | **Y** | **Y** | **Y** | **Y** |
| Question 10 | **Y** | **N** | **Y** | **Y** | **Y** | **Y** | **Y** | **N** | **N** | **N** | **N** | **Y** | **N** | **Y** | **N** |
| Question 11 | **Y** | **Y** | **Y** | **Y** | **Y** | **Y** | **Y** | **Y** | **Y** | **Y** | **Y** | **Y** | **Y** | **Y** | **Y** |
| **Results** | | | | | | | | | | | | | | | |
| Question 12 | **Y** | **Y** | **Y** | **Y** | **Y** | **Y** | **Y** | **Y** | **Y** | **Y** | **Y** | **Y** | **Y** | **Y** | **Y** |
| Question 13 | **N** | **N** | **N** | **N** | **N** | **N** | **N** | **N** | **N** | **N** | **N** | **N** | **N** | **N** | **N** |
| Question 14 | **Y** | **N** | **N** | **N** | **N** | **N** | **N** | **N** | **N** | **N** | **N** | **N** | **N** | **N** | **N** |
| Question 15 | **Y** | **Y** | **Y** | **Y** | **Y** | **Y** | **Y** | **Y** | **Y** | **Y** | **Y** | **Y** | **Y** | **Y** | **Y** |
| Question 16 | **Y** | **Y** | **Y** | **Y** | **Y** | **Y** | **Y** | **Y** | **Y** | **Y** | **Y** | **Y** | **Y** | **Y** | **Y** |
| **Discussion** | | | | | | | | | | | | | | | |
| Question 17 | **Y** | **Y** | **Y** | **Y** | **Y** | **Y** | **Y** | **Y** | **Y** | **Y** | **Y** | **Y** | **Y** | **Y** | **Y** |
| Question 18 | **Y** | **Y** | **Y** | **Y** | **Y** | **Y** | **Y** | **Y** | **Y** | **Y** | **Y** | **N** | **Y** | **Y** | **Y** |
| **Other** | | | | | | | | | | | | | | | |
| Question 19 | **N** | **N** | **N** | **N** | **N** | **N** | **N** | **N** | **N** | **N** | **N** | **N** | **N** | **N** | **N** |
| Question 20 | **Y** | **Y** | **Y** | **Y** | **Y** | **Y** | **Y** | **Y** | **N** | **Y** | **Y** | **Y** | **Y** | **Y** | **Y** |

| **Question** | Rawlings et al. | Razavi et al. | Reis et al. | Richards et al. | Ritchie et al. | Root et al. | Rose et al. | Sadahiro et al. | Salzwedel et al. | Sierra et al. | Singh-Manoux et al. | Singh-Manoux et al. | Singh-Manoux et al. | Smith et al. |
| --- | --- | --- | --- | --- | --- | --- | --- | --- | --- | --- | --- | --- | --- | --- |
| **Introduction** | | | | | | | | | | | | | | |
| Question 1 | **Y** | **Y** | **Y** | **Y** | **Y** | **Y** | **Y** | **Y** | **Y** | **Y** | **Y** | **Y** | **Y** | **Y** |
| **Methods** | | | | | | | | | | | | | | |
| Question 2 | **Y** | **Y** | **Y** | **Y** | **Y** | **Y** | **Y** | **Y** | **Y** | **Y** | **Y** | **Y** | **Y** | **Y** |
| Question 3 | **N** | **N** | **N** | **N** | **N** | **N** | **N** | **N** | **N** | **N** | **N** | **N** | **N** | **N** |
| Question 4 | **Y** | **Y** | **Y** | **Y** | **Y** | **Y** | **Y** | **N** | **Y** | **Y** | **Y** | **Y** | **Y** | **Y** |
| Question 5 | **Y** | **Y** | **Y** | **Y** | **Y** | **Y** | **Y** | **U** | **Y** | **Y** | **Y** | **Y** | **Y** | **U** |
| Question 6 | **Y** | **Y** | **Y** | **Y** | **U** | **Y** | **Y** | **U** | **Y** | **Y** | **Y** | **Y** | **Y** | **U** |
| Question 7 | **N** | **N** | **N** | **N** | **N** | **N** | **N** | **N** | **N** | **N** | **Y** | **N** | **N** | **N** |
| Question 8 | **Y** | **Y** | **Y** | **Y** | **Y** | **Y** | **Y** | **Y** | **Y** | **Y** | **Y** | **Y** | **Y** | **Y** |
| Question 9 | **Y** | **Y** | **Y** | **Y** | **U** | **Y** | **Y** | **Y** | **Y** | **Y** | **Y** | **Y** | **Y** | **Y** |
| Question 10 | **N** | **Y** | **Y** | **N** | **N** | **N** | **N** | **Y** | **Y** | **Y** | **Y** | **Y** | **N** | **Y** |
| Question 11 | **Y** | **Y** | **Y** | **Y** | **Y** | **Y** | **Y** | **N** | **Y** | **Y** | **Y** | **Y** | **Y** | **Y** |
| **Results** | | | | | | | | | | | | | | |
| Question 12 | **Y** | **Y** | **Y** | **N** | **Y** | **Y** | **Y** | **N** | **Y** | **Y** | **N** | **Y** | **Y** | **Y** |
| Question 13 | **N** | **N** | **N** | **N** | **N** | **N** | **N** | **N** | **N** | **N** | **N** | **N** | **N** | **N** |
| Question 14 | **N** | **N** | **N** | **N** | **N** | **N** | **N** | **N** | **N** | **N** | **N** | **N** | **N** | **N** |
| Question 15 | **Y** | **Y** | **Y** | **Y** | **Y** | **Y** | **Y** | **Y** | **Y** | **Y** | **Y** | **Y** | **Y** | **Y** |
| Question 16 | **Y** | **Y** | **Y** | **Y** | **Y** | **Y** | **Y** | **Y** | **Y** | **Y** | **Y** | **Y** | **Y** | **Y** |
| **Discussion** | | | | | | | | | | | | | | |
| Question 17 | **Y** | **Y** | **Y** | **Y** | **Y** | **Y** | **Y** | **Y** | **Y** | **Y** | **Y** | **Y** | **Y** | **Y** |
| Question 18 | **Y** | **Y** | **Y** | **Y** | **N** | **Y** | **Y** | **Y** | **Y** | **Y** | **Y** | **Y** | **Y** | **Y** |
| **Other** | | | | | | | | | | | | | | |
| Question 19 | **N** | **N** | **N** | **N** | **N** | **N** | **N** | **N** | **N** | **N** | **N** | **N** | **N** | **N** |
| Question 20 | **N** | **Y** | **Y** | **N** | **Y** | **Y** | **N** | **Y** | **Y** | **Y** | **Y** | **Y** | **Y** | **N** |
|  |  |  |  |  |  |  |  |  |  |  |  |  |  |  |

| **Question** | Solomon et al. | Sun et al. | Suvila et al. | Szczesnia et al. | Szoeke et al. | Tipnis et al. | Tufvesson et al. | Tuligenga et al. | Udayakumar et al. | Veugen et al. | Walker et al. | Wang et al. | Wang et al. | Wei et al. |
| --- | --- | --- | --- | --- | --- | --- | --- | --- | --- | --- | --- | --- | --- | --- |
| Question 1 | **Y** | **Y** | **Y** | **Y** | **Y** | **Y** | **Y** | **Y** | **Y** | **Y** | **Y** | **Y** | **Y** | **Y** |
| Question 2 | **Y** | **Y** | **Y** | **Y** | **Y** | **Y** | **Y** | **Y** | **Y** | **Y** | **Y** | **Y** | **Y** | **Y** |
| Question 3 | **N** | **N** | **N** | **Y** | **N** | **N** | **N** | **N** | **N** | **N** | **N** | **N** | **N** | **N** |
| Question 4 | **Y** | **Y** | **Y** | **Y** | **Y** | **Y** | **Y** | **Y** | **Y** | **Y** | **Y** | **Y** | **Y** | **Y** |
| Question 5 | **U** | **Y** | **Y** | **Y** | **Y** | **Y** | **Y** | **Y** | **Y** | **Y** | **Y** | **Y** | **Y** | **Y** |
| Question 6 | **U** | **U** | **Y** | **Y** | **Y** | **Y** | **Y** | **Y** | **Y** | **Y** | **Y** | **Y** | **Y** | **U** |
| Question 7 | **N** | **N** | **N** | **N** | **Y** | **N** | **Y** | **N** | **N** | **N** | **Y** | **N** | **N** | **N** |
| Question 8 | **Y** | **Y** | **Y** | **Y** | **Y** | **Y** | **Y** | **Y** | **Y** | **Y** | **Y** | **Y** | **Y** | **Y** |
| Question 9 | **Y** | **Y** | **Y** | **Y** | **Y** | **Y** | **Y** | **Y** | **Y** | **Y** | **Y** | **Y** | **Y** | **Y** |
| Question 10 | **Y** | **Y** | **Y** | **Y** | **Y** | **Y** | **Y** | **Y** | **Y** | **Y** | **Y** | **Y** | **Y** | **Y** |
| Question 11 | **Y** | **Y** | **Y** | **Y** | **Y** | **Y** | **Y** | **Y** | **N** | **Y** | **Y** | **Y** | **Y** | **Y** |
| Question 12 | **Y** | **Y** | **Y** | **Y** | **Y** | **Y** | **Y** | **Y** | **N** | **Y** | **Y** | **Y** | **Y** | **Y** |
| Question 13 | **N** | **N** | **N** | **N** | **N** | **N** | **N** | **N** | **N** | **N** | **N** | **N** | **N** | **N** |
| Question 14 | **N** | **N** | **N** | **N** | **N** | **N** | **N** | **N** | **N** | **N** | **N** | **N** | **N** | **N** |
| Question 15 | **Y** | **Y** | **Y** | **Y** | **Y** | **Y** | **Y** | **Y** | **Y** | **Y** | **Y** | **Y** | **Y** | **Y** |
| Question 16 | **Y** | **Y** | **Y** | **Y** | **Y** | **Y** | **Y** | **Y** | **Y** | **Y** | **Y** | **Y** | **Y** | **Y** |
| Question 17 | **Y** | **Y** | **Y** | **Y** | **Y** | **Y** | **Y** | **Y** | **Y** | **Y** | **Y** | **Y** | **Y** | **Y** |
| Question 18 | **N** | **Y** | **Y** | **Y** | **Y** | **N** | **N** | **Y** | **N** | **Y** | **Y** | **Y** | **Y** | **Y** |
| Question 19 | **N** | **N** | **N** | **N** | **N** | **N** | **N** | **N** | **N** | **N** | **N** | **N** | **N** | **N** |
| Question 20 | **Y** | **Y** | **Y** | **Y** | **N** | **Y** | **Y** | **Y** | **Y** | **Y** | **Y** | **Y** | **Y** | **N** |

| **Question** | Wharton et al. | Whitaker et al. | Wieczorek et al. | Winkler et al. | Wod et al. | Wolf et al. | Wu et al. | Yaffe et al. | Yang et al. | Yano et al. | Yano et al. | Ylilauri et al. | Young et al. | Yulug et al. | ZekiAlHazzouri et al. | Zhang et al. |  |
| --- | --- | --- | --- | --- | --- | --- | --- | --- | --- | --- | --- | --- | --- | --- | --- | --- | --- |
| **Introduction** | | | | | | | | | | | | | | | | | |
| Question 1 | **Y** | **Y** | **Y** | **Y** | **Y** | **Y** | **Y** | **Y** | **Y** | **Y** | **Y** | **Y** | **Y** | **Y** | **Y** | **N** |  |
| **Methods** | | | | | | | | | | | | | | | | | |
| Question 2 | **Y** | **Y** | **Y** | **Y** | **Y** | **Y** | **Y** | **Y** | **Y** | **Y** | **Y** | **Y** | **Y** | **Y** | **Y** | **Y** |  |
| Question 3 | **N** | **N** | **N** | **N** | **N** | **N** | **N** | **N** | **N** | **N** | **N** | **N** | **N** | **N** | **N** | **N** |  |
| Question 4 | **Y** | **Y** | **Y** | **Y** | **Y** | **Y** | **Y** | **Y** | **Y** | **Y** | **Y** | **Y** | **Y** | **Y** | **Y** | **Y** |  |
| Question 5 | **Y** | **Y** | **Y** | **Y** | **Y** | **Y** | **Y** | **Y** | **Y** | **Y** | **Y** | **Y** | **Y** | **U** | **Y** | **Y** |  |
| Question 6 | **Y** | **Y** | **Y** | **Y** | **Y** | **Y** | **Y** | **Y** | **Y** | **Y** | **Y** | **Y** | **Y** | **U** | **Y** | **Y** |  |
| Question 7 | **N** | **N** | **N** | **N** | **N** | **N** | **N** | **N** | **N** | **N** | **N** | **N** | **N** | **N** | **N** | **N** |  |
| Question 8 | **Y** | **Y** | **Y** | **Y** | **Y** | **Y** | **Y** | **Y** | **Y** | **Y** | **Y** | **Y** | **Y** | **Y** | **Y** | **Y** |  |
| Question 9 | **Y** | **Y** | **Y** | **Y** | **Y** | **Y** | **Y** | **Y** | **Y** | **Y** | **Y** | **Y** | **Y** | **Y** | **Y** | **Y** |  |
| Question 10 | **Y** | **N** | **Y** | **N** | **N** | **N** | **Y** | **Y** | **N** | **Y** | **Y** | **Y** | **Y** | **Y** | **N** | **Y** |  |
| Question 11 | **Y** | **Y** | **Y** | **Y** | **Y** | **N** | **Y** | **Y** | **Y** | **Y** | **Y** | **Y** | **Y** | **Y** | **Y** | **Y** |  |
| **Results** | | | | | | | | | | | | | | | | | |
| Question 12 | **Y** | **Y** | **Y** | **Y** | **Y** | **N** | **Y** | **Y** | **Y** | **Y** | **Y** | **Y** | **Y** | **Y** | **Y** | **Y** |  |
| Question 13 | **N** | **N** | **N** | **N** | **N** | **N** | **N** | **N** | **N** | **N** | **N** | **N** | **N** | **N** | **N** | **N** |  |
| Question 14 | **N** | **N** | **N** | **N** | **N** | **N** | **N** | **N** | **N** | **N** | **N** | **N** | **N** | **N** | **N** | **N** |  |
| Question 15 | **Y** | **Y** | **Y** | **Y** | **Y** | **Y** | **Y** | **Y** | **Y** | **Y** | **Y** | **Y** | **Y** | **Y** | **Y** | **Y** |  |
| Question 16 | **Y** | **Y** | **Y** | **Y** | **Y** | **U** | **Y** | **Y** | **Y** | **Y** | **Y** | **Y** | **Y** | **Y** | **Y** | **Y** |  |
| **Discussion** | | | | | | | | | | | | | | | | | |
| Question 17 | **Y** | **Y** | **Y** | **Y** | **Y** | **Y** | **Y** | **Y** | **Y** | **Y** | **Y** | **Y** | **Y** | **Y** | **Y** | **Y** |  |
| Question 18 | **Y** | **Y** | **Y** | **Y** | **Y** | **Y** | **Y** | **Y** | **Y** | **Y** | **Y** | **Y** | **Y** | **Y** | **Y** | **Y** |  |
| **Other** | | | | | | | | | | | | | | | | | |
| Question 19 | **N** | **N** | **U** | **N** | **N** | **N** | **N** | **N** | **N** | **N** | **N** | **N** | **N** | **N** | **N** | **N** |  |
| Question 20 | **Y** | **Y** | **Y** | **Y** | **Y** | **N** | **Y** | **U** | **Y** | **Y** | **Y** | **Y** | **N** | **Y** | **Y** | **N** |  |

*The colour of the included text is representative of the following: *green* indicates a positive impact on the measure of study quality; *red* indicates a negative impact on the measure of study quality; *orange* indicates an unknown impact on the measure of study quality. N: *no*; U: *unsure*; Y: *yes*.

**Supplemental Table B.5.** List of reference studies used for longitudinal cohort studies.

| Study Cohort | Reference Study |
| --- | --- |
| ARIC Cohort | Dearborn-Tomazoset al. (2019) |
| CARDIA Cohort | Suvila et al. (2021) |
| Whitehall Cohort | Brunner et al. (2017) |
| PATH Cohort | Anstey et al. (2014) |
| BHS Cohort | Carmichael et al. (2019) |
| WHAP Cohort | Szoeke et al. (2016) |
| Framingham Cohort | Bangen et al. (2013) |
| SWAN Cohort | Kazlavskaite et al. (2020) |
| Honolulu Cohort | Chosy et al. (2019) |
| MRC-NSHD Cohort | Masi et al. (2018) |
| ELSA-UK Cohort | Olaya et al. (2019) |
| CHARLS Cohort | Zhang et al. (2019) |

**Supplemental Table B.6.** Diabetes status, associated metrics, and cognitive measures across all included studies.

| Author | Year | Fasting Blood Glucose (mg/dl) | HbA1c (mmol/L) | Diabetes Status (N = ) | MeTS (N = ) | Cognitive Variables (Mean and SD Available) |
| --- | --- | --- | --- | --- | --- | --- |
| Albanese et al. | **2012** | - | Total Cohort: Males = 5.6 (0.7), N = 897; Females = 5.6 (0.6), N = 920 | - | - | - |
| Aliberti et al | **2020** | - | - | Aged 50-64 (Total): Diabetes = 220,  No Diabetes = 504 | - | - |
| Almani et al. | **2021** | - | HbA1c range overall (%): 4.1 – 13.2  HbA1c in cognitively impaired patients (%): 8.2 – 12.7 | Total = 278 (males = 155, females = 123) | - | - |
| Alves de Moraes | **2002** | - | - | Baseline (Total):  Diabetes = 943  No Diabetes = 7115 | - | Verbal Memory: Delayed Recall: 3.8 (SE = 0.01), 6.7 (SE = 0.01), 6.6 (SE = 0.02), 6.4 (SE = 0.02)  Working Memory:  DSST (Total): Normotensive = 48.9 (0.13), Incident Hypertension = 45.5 (0.26), Controlled Hypertension = 43.5 (0.32), Partially Controlled Hypertension = 42.8 (0.33), Uncontrolled Hypertension = 42.4 (0.38) |
| Anstey et al. | **2014** | - | - | Baseline (Total - Persisting to Wave 3): Diabetes = 38  No Diabetes = 3114 | - | Verbal Memory: Immediate Recall (Total): 7.818 (SE = 0.04) Delayed Recall (Total): 6.987 (SE = 0.05) Spot the Word (Total): 50.463 (SE = 0.11) |
| Babaei et al. | **2013** | Total:  HC = 94.28 (3.72), N = 10  MC = 212.57 (15.41), N = 10  HE = 119 (47.13), N = 11  ME = 130.87 (27.12), N = 11 | - | - | Total:  MeTS = 21  No MeTS = 21 | - |
| Backestrom et al. | **2015** | Male = 95.4 (10.08), N = 127  Female = 93.6 (9.72), N = 164  Total = 95.4 (9.9), N = 291 | - | - | - | Episodic memory: Summary Score: Males = 6.62 (1.3), Females = 7.29, Total = 7 (1.39)  Semantic Memory Score: Males (Total): 15.2 (3.03), 40 Years: 15.56 (2.07), 50 Years: 15.67 (2.9), 60 Years: 14.33 (3.72) Females (Total): 15.96 (2.82), 40 Years: 16.86 (2.1), 50 Years: 15.86 (3.07), 60 Years: 15.39 (2.93) Total: 15.6 (2.94), 40 Years: 16.27 (2.17), 50 Years: 15.77 (2.98), 60 Years: 14.96 (3.3) |
| Bancks et al. | **2017** | Total = 100.8 (9), N = 10,133 | - | Total:  Diabetes = 1723  No Diabetes = 8202 | - | Verbal Memory: Delayed Recall (Total): 6.8 (1.5)  Working Memory: DSST (Total): 46.9 (13.3) |
| Bangen et al. | **2013** | - | - | Total:  Diabetes = 72  No Diabetes = 1364 | - | Verbal Memory: Delayed Recall (Total): 11 (4)  Attention TMT-A (Total): 0.5 (0.2)  Intelligence WAIS (Male): 17 (3)  Visuospatial Organisation Hooper Visual Organisation Test (Total): 25 (3) |
| Bayes-Marin et al. | **2020** | - | - | Baseline (50-64 yrs Total): Diabetes = 73  No Diabetes = 560 | - | Verbal Memeory: Immediate Recall (Total): 17.36 Delayed Recall: 5.35 |
| Blodgett et al. | **2020** | - | - | Age 53:  Male = 57; Female = 43  Age 60–64:  Male = 129; Female = 99  Age 69:  Male = 175; Female = 136 | - | Verbal Memory  Age 53: Male = 23.0 (6.2), n = 1,397; Female = 24.9 (6.2), n = 1473  Age 60–64: Male = 23.0 (5.9), n = 1,023; Female = 25.4 (6.1), n = 1,127  Age 69: Male = 21.2 (6.0), n = 1,005; Female = 23.1 (6.0), n = 1057 |
| Boots et al. | **2015** | Wave 2 Total Participants = 96.38 (13.75), N = 315 | - | Wave 2 Participants (Total): Diabetes = 10  No Diabetes = 341 | - | Global Cognition MMSE (Total): 29.47 (0.88) IQCODE (Total): 43.38 (3.01) |
| Bressler et al. | **2013** | Total Whites = 108 (30.6), N = 8360 Total African American = 122.4 (52.2), N = 2069 | - | Total Whites:  Diabetes = 864,  No Diabetes = 7490  Total African American:  Diabetes = 423  No Diabetes = 1633 | - | Verbal Memory: Delayed Recall (Total) White (rs9939609): TT: 6.8 (1.4); AT: 6.8 (1.4); AA: 6.9 (1.4) White ( rs17817449): TT: 6.8 (1.4); GT: 6.8 (1.4); GG: 6.9 (1.4) White (rs805136): CC: 6.8 (1.4); AC: 6.8 (1.4); AA: 6.9 (1.4) White ( rs1421085): TT: 6.8 (1.4); CT: 6.8 (1.4) ; CC:6.9 (1.4) African American (rs9939609): TT: 6.3 (1.5); AT: 6.3 (1.6); AA: 6.4 (1.6) African American ( rs17817449): TT: 6.3 (1.6); GT: 6.3 (1.6); GG: 6.4 (1.6) African American (rs805136): CC: 6.3 (1.5); AC: 6.3 (1.6); AA: 6.3 (1.6) African American ( rs1421085): TT: 6.4 (1.6); CT: 6.2 (1.6) ; CC:6.3 (1.4)  Working Memory DSST (Total) White (rs9939609): TT: 49.5 (11.2); AT: 49.9 (11.2); AA: 49.9 (11.3) White ( rs17817449): TT: 49.5 (11.2); GT: 49.9 (11.2); GG: 49.9 (11.4) White (rs805136): CC: 49.4 (11.1); AC: 49.9 (11.2); AA: 49.9 (11.4) White ( rs1421085): TT: 49.5 (11.2); CT: 49.9 (11.2) ; CC:49.9 (11.3) African American (rs9939609): TT: 33.6 (13.1); AT: 32.6 (13.1); AA: 33.8 (13) African American ( rs17817449): TT: 33.1 (13.2); GT: 32.7 (13.0); GG: 34.3 (13.2) African American (rs805136): CC: 33.0 (13.3); AC: 32.9 (13.1); AA: 34.1 (13.2) African American ( rs1421085): TT: 33.2 (13.2); CT: 32.8 (13.0); CC: 33.6 (11.9) |
| Britton et al. | **2004** | - | - | - | - | Working Memory Composite Score: Males = 6.85 (2.33), Females = 6.87 (2.72)  Inductive Reasoning AH-4: Male = 48.61 (10.07), Female = 41.21 (12.41) |
| Brunner et al. | **2017** | Clinical visits 5 & 6: 55-64 years = 66.6 (151.2), N = 441; 203 (11.3), N = 1790 | - | - | - | - |
| Carmichael et al. | **2019** | Above age 40:  Low-Normal Mean FPG (n = 6) = 79.3 ± 3.13  High-Normal or Impaired Mean FPG (n = 42) = 97.4 ± 12.9 | - | - | - | Memory:  Logical memory  This study = 17.2 (6.9), Recent cognitive assessment = 16.0 (7.3)  Executive function:  Digit Coding  This study = 65.6 (16.4)  Recent cognitive assessment = 58.8 (17.7)  DSBT  This study = 7.7 (2.2)  Recent cognitive assessment = 7.7 (2.5)  TMT-B  This study = 57.4 (28.8)  Recent cognitive assessment = 62.3 (30.1) |
| Cerhan et al. | **1998** | - | - | T2DM: Total = 12,279  (Male = 703, Female = 861)  Without T2DM: Total = 1564  (Male = 5,449, Female = 6,830) | - | Memory  Delayed Word Recall  Male  Age 45-49 = 6.7 ± 1.6  Age 50-54 = 6.6 ± 1.4  Age 55-59 = 6.4 ± 1.4  Age 60-64 = 6.1 ± 1.5  Age 65-69 = 5.9 ± 1.6  Diabetes = 6.00  No Diabetes = 6.34  Female  Age 45-49 = 7.3 ± 1.4  Age 50-54 = 7.1 ± 1.4  Age 55-59 = 6.9 ± 1.5  Age 60-64 = 6.6 ± 1.5  Age 65-69 = 6.3 ± 1.6  Diabetes = 6.64  No Diabetes = 6.90  DSST  Male  Age 45-49 = 46.6 ± 12.9  Age 50-54 = 45.0 ± 13.2  Age 55-59 = 43.1 ± 13.1  Age 60-64 = 40.6 ± 12.6  Age 65-69 = 37.8 ± 13.3  Diabetes = 40.2  No Diabetes = 41.8  Female  Age 45-49 = 51.7 ± 14.2  Age 50-54 = 49.2 ± 14.4  Age 55-59 = 46.8 ± 14.4  Age 60-64 = 43.8 ± 13.6  Age 65-69 = 40.1 ± 13.8  Diabetes = 44.8  No Diabetes = 47.4  Executive Function  WFT  Male  Age 45-49 = 34.8 ± 12.6  Age 50-54 = 33.2 ± 12.5  Age 55-59 = 32.6 ± 12.7  Age 60-64 = 32.0 ± 12.6  Age 65-69 = 31.6 ± 13.0  Diabetes = 31.6  No Diabetes = 32.3  Female  Age 45-49 = 35.8 ± 12.0  Age 50-54 = 34.6 ± 12.4  Age 55-59 = 33.6 ± 12.1  Age 60-64 = 32.7 ± 12.1  Age 65-69 = 31.8 ± 12.6  Diabetes = 32.4  No Diabetes = 34.3 |
| Chen et al. | **2015** | - | - | - | - | Psychomotor Speed SDMT: Female: Normotensive = 52.8, Pre-hypertensive = 50, Hypertensive = 47.1 |
| Chen et al. | **2018** | - | - | Total: Diabetes = 1818  No Diabetes = 10,697 | - | Verbal Memory Delayed Recall: Total: 6.7 (1.5); No Incident AF: 6.7 (1.5); Incident AF: 6.5 (1.5)  Working Memory DSST (Total): 45.3 (13.8) |
| Cherbuin et al. | **2009** | - | - | Normal at Wave 2 (Total): Diabetes = 135,  No Diabetes = 1863  MCI at Wave 2 (Total): Diabetes = 1,  No Diabetes = 17  Any-MCD at Wave 2 (Total): Diabetes = 9,  No Diabetes = 55 | - | Global Cognition MMSE (Total): 29.37 (0.895) |
| Chosy et al. | **2019** | - | - | - | - | - |
| Christman et al. | **2011** | Total = 109.8 (34.2), N = 8958 | Total = 5.4, N = 8958  Non-diagnosed Diabetes  HbA1c <5.7% = 5.3, N = 6589  HbA1c 5.7-6.5% = 5.9, N = 1542  HbA1c ≥6.5% = 6.8, N = 311  Diagnosed Diabetes  HbA1c <7.0% = 6.3, N = 144  HbA1c 7.0-8.0% = 7.7, N = 107  HbA1c ≥8.0% = 9.8, N = 265 | Total: Diabetes = 516  No Diabetes = 8442 | - | Verbal Memory Delayed Recall: Total = 6.7 (1.5)  Working Memory DSST (Total): 47.4 (12.8) |
| Chuang et al. | **2023** | Males = 103.3 (27.1)  Females = 101.6 (29.2) | - | Total  Diabetes = 44  No Diabetes = 574 | - | Global Cognition  MMSE: Male = 28.5 (1.9), Female = 27.9 (2.8) |
| Cohen-Manheim et al. | **2016** | Total = 91.8 (9), N = 50 | - | - | - | - |
| Creavin et al. | **2012** | Phase 1 (Total) = 86.4, N = 2512 | - | Phase 1 (Total):  Diabetes = 75  No Diabetes = 2437  Phase 2 (Total):  Diabetes = 168  No Diabetes = 2382  Phase 3 (Total):  Diabetes = 184  No Diabetes = 1862 | Phase 1 (Total):  MeTS = 75  No MeTS = 2437  Phase 2 (Total):  MeTS = 168  No MeTS = 2230  Phase 3 (Total):  MeTS = 205  No MeTS = 1841 | Inductive Reasoning AH-4 (Total): 73.1  Global Cognition MMSE (Total): 27 CAMCOG (Total): 90 NART (Total): 28 |
| Cui et al. | **2016** | Control = 90 (7.74), N = 155  Hypertensive = 89.28 (10.44, N = 278 | - | - | - | Intelligence WAIS (Total): Control (Normotensive): 99.23 (8.64), Non-Treated Hypertension: 98.77 (9.46), Treated Hypertension: 104.53 (6.68), Compliant Hypertension: 105.31 (9.02), Non-Compliant Hypertension: 102.77 (9.85)  Woking Memory CMS Score (Total): Control (Normotensive): 84.11 (9.96), Non-Treated Hypertension: 70.02 (16.53), Treated Hypertension: 74.91 (14.23), Compliant Hypertension: 81.86 (11.85), Non-Compliant Hypertension: 65.12 (12.43)  Global Cognition MMSE (Total): Control (Normotensive): 27.59 (2.25), Non-Treated Hypertension: 26.98 (3.04), Treated Hypertension: 728.91 (1.06), Compliant Hypertension: 29.04 (1.62), Non-Compliant Hypertension: 27.65 (2.18) |
| de Menezes et al. | **2021** | - | - | Baseline (Total): Diabetes = 1617  No Diabetes = 5446 | - | Working Memory DSST (Total): 36.8 (6.1) |
| Dearborn et al. | **2014** | No MetS: Males = 99.8 (15.9), Females = 95.2 (14.9) MetS: Males = 115.6 (32.5), Females = 116 (41.2) | - | No MetS: Diabetes:  Males = 102, Females = 66  MetS: Diabetes:  Males = 289, Females = 395 | MeTS = 3830  No MeTS = 7036 | Verbal Memory Immediate Recall:  MetS: Males = 6.5 (1.5), Females = 7.1 (1.4) No MetS: Males = 6.3 (1.4), Females = 6.8 (1.4)  Working Memory DSST: MetS: Males = 44.5 (12.7), Females = 50.4 (12.9) No MetS: Males = 43.9 (11.9), Females = 45 (14.1) |
| Dearborn-Tomazos et al. | **2019** | - | - | Total:  Diabetes = 1437  No Diabetes = 12,151 | - | Means and SD not available for extraction |
| Debette et al. | **2011** | - | - | No Diabetes Data | - | Verbal Memory Delayed Recall (Total): 10.83 (3.5)  Visuospatial Organisation  VR-d Test (Total): 8.62 (3.24) |
| Del Vecchio et al. | **2023** | - | - | Diabetes  Total: n = 7; Control: n = 1; Study: n = 6  No Diabetes  Total: n = 105; Control: n = 30; Study: n = 75 | - | MMSE  Total: 25.8 (2.2); Control: 26.5 (2.3); Study: 25.6 (2.2)  MoCA  Total: 25.2 (2.7); Control: 26.5 (2.9); Study: 24.6 (2.4) |
| Derby et al. | **2021** | Glucose≥100mg/dL: n = 188 (16.5%) | - | Diabetes: n = 82  No diabetes: n = 1,057 | - | SDMT: 58.8 (10.3)  High glucose (Yes, No): 57.1 (11.3), 59.1(10.1)  Diabetes (Yes, No): 53.9 (11.6), 59.2 (10.1)  DSBT: 7.0 (2.3)  High glucose (Yes, No): 6.5 (2.2), 7.1 (2.3)  Diabetes (Yes, No): 6.1 (2.0), 7.0 (2.3)  EBMT: 10 (10, 12)  High glucose (Yes, No): 2 (612), 1 (562)  Diabetes (Yes, No): 2 (634), 1 (565) |
| Dintica et al. | **2022** | No MetS: 93.7 (17.8)  MetS: 119.5 (43.9) | - | - | No MetS: 2346  MetS: 534 | RAVLT Delay  No MetS: 8.7 (3.4)  MetS: 7.8 (3.4)  DSST  No MetS: 69.1 (16.6)  MetS: 63.7 (16.6)  Stroop Test  No MetS: 22.2 (11.1)  MetS: 24.8 (14.1)  Verbal Fluency  No MetS: 31.5 (8.3)  MetS: 29.6 (7.9)  MoCA  No MetS: 24.1 (3.9)  MetS: 23.1 (3.9)  Global Composite Score  No MetS: 0.06 (0.7)  MetS: -0.18 (0.7) |
| Dixon et al. | **2021** | - | - | European American = 94  African American = 102  Asian American = 93 | - | Executive Function  SDMT  European American = 59.95 ± 9.55  African American = 51.83 ± 11.18  Asian American = 61.58 ± 8.8  Working Memory  DSB  European American = 7.55 ± 2.32  African American = 6.02 ± 2.27  Asian American = 6.77 ± 1.91  Verbal Memory  EBMT  European American = 10.54 ± 1.58  African American = 9.74 ± 1.97  Asian American = 10.21 ± 1.58 |
| Elbaz et al. | **2014** | - | - | Follow-up 1 (Total): Diabetes = 95  No Diabetes = 4604 | - | Inductive Reasoning AH-4 (Total): 47 (10) |
| Elkins et al. | **2005** | - | - | Baseline (Visit 1 – Total):  Diabetes = 14  No Diabetes = 12,082 | - | Verbal Memory Delayed Recall (Total): 7 (1.5)  Working Memory DSST (Total): 45 (14) |
| Elmassry et al. | **2015** | - | - | - | - | Attention TMT-A (Total): Hypetension Patients = 50.6 (4.2), Controls (Normotensive) = 43.8 (3.1) 5-Choice Movement Time (Total): Hypertension Patients = 371.3, Controls (Normotensive) = 369.4  Global Cognition MMSE (Total): Hypertension Patients = 19.8 (2.3), Controls (Normotensive) = 24.8 (1.7) |
| Fava et al. | **2013** | Group A = 85.4 ± 9.2  Group B = 84.8 ± 9.2 | Group A = 4 ± 0.4  Group B = 3.8 ± 0.5 | - | Group A = 9  Group B = 9 | Corsi  Group A = 2.1 ± 0.9  Group B = 0.6 ± 0.4  DSB  Group A = 1.8 ± 0.5  Group B = 0.5 ±0.2  CVLT (Free recall)  Group A = 1.2 ± 0.7  Group B = 0.6 ± 0.3  CVLT (Recognition)  Group A = 1.9 ± 0.6  Group B = 0.9 ± 0.3  RVDLT (Free recall)  Group A = 2.1 ± 0.5  Group B = 0.5 ± 0.2  RVDLT (Recognition)  Group A = 1.4 ± 0.6  Group B = 0.4 ± 0.1  TMT  Group A = 1.9 ± 0.7  Group B = 0.6 ± 0.2  IQ  Group A = 1.7 ± 0.5  Group B = 0.5 ± 0.2 |
| Ferguson et al. | **2018** | - | - | Total: Diabetes = 66  No Diabetes = 568 | - | - |
| Ford et al. | **2010** | - | - | - | Female (Total):  MeTS = 393  No MeTS = 1610 | Verbal Memory East Boston Memory (Females) = 10.2  Working Memory Digit Span Backwards (Females) = 6.6 |
| Fuh et al. | **2013** | Normal = 86.4 ± 12.6  Impaired glucose tolerance = 91.8 ± 14.4  Diabetes mellitus = 156.6 ± 63 | - | Normal (N = 144)  Impaired glucose tolerance (N = 68)  Diabetes mellitus (N = 72) | - | RAVLT  Normal: 9.9 (2.5)  Impaired Glucose Control:10.6 (2.2)  Diabetes: 9.8 (2.8)  Continuous Recognition Paradigm of Kimura  Normal: 69.4 (5.7)  Impaired Glucose Control: 69.9 (5.5)  Diabetes: 68.2 (6.4)  VFT  Normal: 13.6 (3.9)  Impaired Glucose Control: 14.5 (4.0)  Diabetes: 13.7 (4.1)  TMT-A  Normal: 83.7 (46.2)  Impaired Glucose Control: 74.8 (30.8)  Diabetes: 91.4 (43.1)  TMT-B  Normal: 116.1 (43.2)  Impaired Glucose Control: 129.0 (60.9)  Diabetes: 135.8 (62.7)  DSFT  Normal: 9.2 (2.5)  Impaired Glucose Control: 9.4 (2.6)  Diabetes: 7.9 (2.7)  DSBT  Normal: 2.9 (1.6)  Impaired Glucose Control:3.3 (1.9)  Diabetes: 2.7 (1.8) |
| Gerasimenko et al. | **2017** | - | - | - | - | Working Memory McNair Survey (Total): Control Patients: 20.05 (2.13); Patient Group 2 (45-65 years): 27.64 (1.42); Patient Group 3 (>56 years): 28.78 (2.81); Patient Group 1 (<5 years): 30.07 (2.61; Patient Group 2 (5-10 years): 27.4 (1.25); Patient Group 3 (>10 years): 30.07 (2.61)  Global Cognition MoCA (Total): Control Patients: 26.95 (0.38); Patient Group 2 (45-65 years): 25.59 (0.29); Patient Group 3 (>56 years): 24.87 (0.43); Patient Group 1 (<5 years): 24.43 (0.26); Patient Group 2 (5-10 years): 25.5 (0.35); Patient Group 3 (>10 years): 24.23 (0.28) |
| Gerber et al. | **2021** | - | - | Overall: 391  Liver attenuation: No NAFLD >51 HU: 187  Mild NAFLD >40–51 HU: 82  Severe NAFLD ≤40 HU: 122 | - | DSST  Overall: 70.0 (16.0)  Liver attenuation: No NAFLD >51 HU: 70.6 (16.3)  Mild NAFLD >40–51 HU: 67.6 (15.6)  Severe NAFLD ≤40 HU: 68.9 (14.4)  RAVLT  Overall: 8.3 (3.3)  Liver attenuation: No NAFLD >51 HU: 8.4 (3.3)  Mild NAFLD >40–51 HU: 7.9 (3.1)  Severe NAFLD ≤40 HU:8.0 (3.1)  Stroop  Overall: 22.9 (10.8)  Liver attenuation: No NAFLD >51 HU: 22.7 (10.7)  Mild NAFLD >40–51 HU: 24.3 (11.6)  Severe NAFLD ≤40 HU:22.9 (10.2) |
| Giugliano et al. | **2018** | Control (Baseline - Total): 95.7 (11.2), N = 18 Active Treatment (Baseline - Total): 93.1 (7.5), N = 18 | - | - | - | - |
| Gonzalez et al. | **2018** | Total = 107.21 (36.68), N = 7397  White = 104.63 (29.54), N = 5467  Black = 116.06 (53.52), N = 1930 | - | Total:  Diabetes = 583  No Diabetes = 6814 | - | - |
| Gottesman et al. | **2017** | - | - | Total: Diabetes = 1889  No Diabetes = 13,855 | - | - |
| Gottesman et al. | **2014** | - | - | White (Normotensive): Diabetes = 239  No Diabetes = 4083  White (Prehypertensive): Diabetes = 255  No Diabetes = 2019  White (Hypertensive): Diabetes = 720  No Diabetes = 2931  Black (Normotensive): Diabetes = 114  No Diabetes = 665  Black (Prehypertensive): Diabetes = 124  No Diabetes = 477  Black (Hypertensive): Diabetes = 567  No Diabetes = 1282 | - | Verbal Memory Delayed Recall (Total) White (Normotensive): 6.92 (1.42); White (Pre-hypertensive): 6.73 (1.49); White (Hypertension): 6.63 (1.42) Black (Normotensive): 6.34 (1.65); Black (Pre-hypertensive): 6.19 (1.62); Black (Hypertension): 6 (1.65)  Working Memory DSST (Total) White (Normotensive): 50.6 (11.3); White (Pre-hypertensive): 48.9 (11.6); White (Hypertension):46.9 (11.5) Black (Normotensive): 34.1 (14.2); Black (Pre-hypertensive): 32.1 (12.7); Black (Hypertension): 29.6 (12.9) |
| Gourley et al. | **2020** | - | - | Total: Diabetes = 18  No Diabetes = 114 | - | - |
| Gupta et al. | **2008** | - | - | - | - | Global Cognition MMSE (Total):  Not taking drugs: 26.94±2.53  β-Blockers: 24.5±1.58  Diuretics: 28±1.58  CCB: 27±1.82  CCB+ β-Blockers: 25.35±4.18  CCB+ nitrates: 28±1.58  Treatment Compliance <25%: 27.08±2.55  Treatment Compliance 51-75%: 22.5±3.97  Treatment Compliance 100%: 26.41±2.92 |
| Gwizdala et al. | **2023** | Total: 85.87 ± 18.06, n = 1108  Males: 89.03 ± 19.09, n = 437  Females: 83.81 ± 17.07, n = 671 | - | - | - | Global Cognition: Total = 0.01 ± 5.36, Males = −0.75 ± 5.12, Females = 0.53 ± 5.47  Logical memory I: Total = 0 ± 1 , Males = −0.08 ± 1, Females = 0.06 ± 0.99  Logical memory II: Total = 0 ±1, Males = −0.11 ± 1, Females = 0.08 ± 0.99  Logical memory II r: Total = 0 ± 0.99, Males = −0.11 ± 1.05, Females = 0.09 ± 0.95  DSF: Total = 0 ± 1, Males = 0.02 ± 0.99, Females = -0.01 ± 1.01  DSB: Total = 0 ± 1, Males = -0.04 ± 1.02, Females = 0.03 ± 0.99  TMT-A: Total = 0 ± 1, Males = 0.08 ± 0.93, Females = -0.06 ± 1.03  TMT-B: Total = 0 ± 1, Males = 0.07 ± 0.96, Females = -0.05 ± 1.02 |
| Hajjar et al. | **2016** | - | - | Total: Diabetes = 34  No Diabetes = 557 | - | Working Memory DSST (Total): 54.5 (0.4) |
| Hajjar et al. | **2018** | - | - | Total: Diabetes = 20  No Diabetes = 491 | - | Working Memory DSST (Total): 55 (0.4) |
| Hakamada-Taguchi et al. | **2002** | Total = 108 (27), N = 26 | - | - | - | Working Memory DSST (Total): 67.4 (1.07) |
| Haley et al. | **2010** | Control (Total): 97.2 (17.9), N = 25 Metabolic Syndrome (Total) = 119.6 (48.8), N = 13 | - | - | Total:  MeTS = 13  No MeTS = 25 | Intelligence WAIS (Total): 112.6 (11.5) |
| Hawkins et al. | **2018** | - | - | - | - | Working Memory WAIS-IV Digit Sequencing: Males = 38.6 (3.9), Females = 38.9 (4.7) |
| Henriksen et al. | **2017** | - | - | - | - | Global Cognition MMSE (Total): No Decline = 30, Decline = 29 BPP (Total): No Decline = 46, Decline = 48 IST (Total): No Decline = 21, Decline = 44 ACE (Total): No Decline = 93, Decline = 97 |
| Hitesh et al. | **2023** | - | Total = 8.2 ± 2.33 | Diabetes = 200  No Diabetes = 200 | - | MMSE  Diabetes = 23.20 ± 1.65  No Diabetes = 28.99 ± 0.86 |
| Hoffmann et al. | **2021** | - | - | Normal BP (T0 - Total): Diabetes = 40  No Diabetes = 652  Prevelant Hypertension (>10 yrs, T0 - Total):  Diabetes = 264  No Diabetes = 881  Incident Hypertension T1 (>5 yrs, T0 - Total):  Diabetes = 54  No Diabetes = 312  Incident Hypertension T2 (<5 yrs, T0 - Total):  Diabetes = 28  No Diabetes = 189  Temporary Hypertension (T0 - Total):  Diabetes = 38  No Diabetes 291 | - | - |
| Hossain et al. | **2020** | - | - | Males:  Diabetes = 22  No Diabetes = 80  Females:  Diabetes = 38  No Diabetes = 88 | - | - |
| Houle et al. | **2019** | - | - | Males: Diabetes = 207,  No diabetes = 1933  Females: Diabetes = 288, No Diabetes = 2220 Total: Diabetes = 495, No Diabetes = 4153 | - | Global Cognition HRS Composite Score: Males = 14.2 (4.15), Females = 14.44 (3.96), Total = 14.31 (4.06) |
| Ihle-Hansen et al. | **2019** | - | - | Males: Diabetes = 195, No Diabetes = 1579 Females: Diabetes = 81, No Diabetes = 1558 Total: Diabetes = 276, No Diabetes = 3173 | - | Global Cognition  MoCA: Males = 25 (2.9), Females = 25.5 (2.9), Total = 25.3 (2.9) |
| Janssen et al. | **2024** | Total = 88.6 ± 22.4  Black = 85.5 ± 24.9  White = 91.4 ± 19.6 | - | - | - | Processing speed  Total = 58.2 ± 11.1, Black = 55.9 ± 12.2, White = 60.3 ± 9.7  Working memory  Total = 6.7 ± 2.1, Black = 6.3 ± 2.0, White = 7.1 ± 2.0 |
| Jia et al. | **2021** | - | - | Total = 478 | - | Global Cognition  MMSE  Age 50-64 years: 26.5 ± 4.1  Age 65-74: 25.5 ± 4.7  Age 75+: 23.2 ± 6.0  T2DM: 25.5 ± 4.7  No T2DM: 25.5 ± 4.9  MoCA  Age 50-64 years: 23.8 ± 5.5  Age 65-74: 22.5 ± 5.9  Age 75+: 20.0 ± 6.8  T2DM: 22.6 ± 5.8  No T2DM: 22.6 ± 6.1 |
| John et al. | **2021** | - | - | - | - | - |
| Kaffashian et al. | **2013** | - | - | Total: Diabetes = 225, No Diabetes = 7605 | - | - |
| Kaffashian et al. | **2013** | - | - | No Diabetes Data | - | - |
| Kaffashian et al. | **2011** | - | - | Male:  Diabetes = 133,  No Diabetes = 3353  Females:  Diabetes = 46,  No Diabetes = 1295 | - | Verbal Memory Delayed Word Recall: Males = 6.9 (2.3), Females = 7.1 (2.7)  Inductive Reasoning AH-4: Male = 49.2 (9.5), Female = 42.9 (11.6) |
| Kalmijn et al. | **2002** | - | - | No Diabetes Data | - | Verbal Memory Delayed Word Recall (Total): Never Smokers = 8.2, Former Smokers = 7.9, Current Smokers = 7.9 |
| Kazlauskaite et al. | **2020** | - | - | No Diabetes Data | Total:  MeTS = 635  No MeTS = 1514 | - |
| Knopman et al. | **2001** | - | - | Visit 4 (Total): Diabetes = 629  No Diabetes = 5339 | - | Verbal Memeory: Delayed Recall (Total): 6.94 (1.4)  Working Memory DSST (Total): 48.62 (13.5) |
| Knopman et al. | **2018** | - | - | Normal (Visit 1 - Total): Diabetes = 237  No Diabetes = 4506 | - | - |
| Knopman et al. | **2009** | - | - | Visit 2 (Cognitive Baseline - Total): Diabetes = 113  No Diabetes = 1017 | Visit 2 (Cognitive Baseline - Total): MeTS = 520  No MeTS = 610 | Verbal Memeory: Delayed Recall (Total): 6.6 (1.41)  Working Memory DSST (Total): 41.1 (13.24) |
| Kohde et al. | **2012** | MetS (Total) = 101.7 (14.7), N = 60 Controls (Total) = 92.5 (5.3), N = 60 | - | - | Total:  MeTS = 60  No MeTS = 60 | Attention Simple/Selective Reaction Time (Total) MetS: VSRT 309.9±74.9, WBSRTC1 424.0±89.9, WBSRTC2 752.9±147.9, WBSRTC2‑C1 328.9±87.7  No MetS: VSRT 278.2±42.3, WBSRTC1 387.3±83.2, WBSRTC2 669.5±106.1, WBSRTC2‑C1 282.2±69.1   Choice Reaction Time Simple/Selective Reaction Time (Total) MetS: VCRT 372±104.1, WBCRTC1 608.1±132.7, WBCRTC2 1022.4±183.1, WBCRTC2‑C1 413.6±109.5 No MetS: VCRT 331.9±58.5, WBCRTC1 513±76.6, WBCRTC2 852.3±98.4, WBCRTC2‑C1 339.3±76.5 |
| Kovacs et al. | **2014** | Healthy controls (free of hypertension or hyperlipidemia - Total): 90.9 (1.8), N = 44 Normotensive subjects with elevated LDL–C levels (Total): 94.5 (1.44), N = 41 Hypertensive patients with normal LDL–C levels (Total): 91.8 (1.44), N = 49 Hypertensive patients with elevated LDL–C levels (Total): 95.94 (2.16), N = 23 | - | - | - | Verbal Memory RAVLT (Summary Metric) Healthy controls (free of hypertension or hyperlipidemia - Total): 13.19 (SE = 0.38) Normotensive subjects with elevated LDL–C levels (Total): 13.05 (SE = 0.42) Hypertensive patients with normal LDL–C levels (Total): 12.28 (SE = 0.44) Hypertensive patients with elevated LDL–C levels (Total): 11.48 (SE = 0.86)  Attention TMT-A Healthy controls (free of hypertension or hyperlipidemia - Total): 32.25 (2.11) Normotensive subjects with elevated LDL–C levels (Total): 33.11 (2.49) Hypertensive patients with normal LDL–C levels (Total): 27.51 (1.67) Hypertensive patients with elevated LDL–C levels (Total): 35.95 (3)  Simple/Selective Reaction Time Healthy controls (free of hypertension or hyperlipidemia - Total): 0.62 (0.01) Normotensive subjects with elevated LDL–C levels (Total): 0.65 (0.01) Hypertensive patients with normal LDL–C levels (Total): 0.65 (0.02) Hypertensive patients with elevated LDL–C levels (Total): 0.64 (0.03)  Choice Reaction Time Healthy controls (free of hypertension or hyperlipidemia - Total): 0.52 (0.01) Normotensive subjects with elevated LDL–C levels (Total): 0.54 (0.01) Hypertensive patients with normal LDL–C levels (Total): 0.56 (0.01) Hypertensive patients with elevated LDL–C levels (Total): 0.56 (0.02)  Working Memory Digit Span Backwards  Healthy controls (free of hypertension or hyperlipidemia - Total): 11.7 (0.31) Normotensive subjects with elevated LDL–C levels (Total): 11.97 (0.42) Hypertensive patients with normal LDL–C levels (Total): 10.07 (0.3) Hypertensive patients with elevated LDL–C levels (Total): 10 (0.54)  Psychomotor Speed SDMT (Total) Healthy controls (free of hypertension or hyperlipidemia - Total): 52.77 (1.5) Normotensive subjects with elevated LDL–C levels (Total): 49.63 (1.43) Hypertensive patients with normal LDL–C levels (Total): 50.6 (1.34) Hypertensive patients with elevated LDL–C levels (Total): 44.33 (2.69)  Visuospatial Organisation Block Design Test Healthy controls (free of hypertension or hyperlipidemia - Total): 26.07 (0.53) Normotensive subjects with elevated LDL–C levels (Total): 25.28 (0.53) Hypertensive patients with normal LDL–C levels (Total): 25.67 (0.58) Hypertensive patients with elevated LDL–C levels (Total): 24.9 (0.83) |
| Kumar et al. | **2008** | - | - | Diabetes = 428  Non-Diabetes = 465 | - | Diabetics; Non-Diabetics  MMSE: 29.03 (1.65) 29.40 (0.92)  Immediate recall: 6.95 (2.29) 7.53 (2.05)  Delayed recall: 6.18 (2.24) 6.67 (2.24)  Purdue Pegboard-both hands: 9.87 (1.36) 10.66 (1.62)  Purdue Pegboard-dominant hand: 12.31 (1.38) 13.59 (1.95)  Purdue Pegboard-nondominant hand: 12.33 (1.72) 13.06 (1.85)  Mean RT (choice): 0.32 (0.03) 0.32 (0.04)  Mean RT (simple): 0.26 (0.03) 0.25 (0.05)  SDMT: 47.97 (7,94) 51.48 (8.56)  Spot-the-Word test: 51.23 (6.53) 52.49 (5.70) |
| Kumar et al., | **2020** | - | - | Total:  Diabetes = 2  No Diabetes = 78 | - | Attention TMT-A (Total): White = 70, African American = 81 Digit Span Forward (Total): White = 7, African American = 6.5  Intelligence  Mental Rotation (Total): White = 18.5, African American = 17.5  Working Memory Digit Span Backwards: Males = 5, Females = 4  Global Cognition MoCA (Total): White = 27, African American = 25 MINT (Total): White = 31, African American = 29 |
| Kumari et al., | **2005** | NGT: Male = 91.8, N = 3407, Female = 88.2, N = 1334 IGT: Male = 93.6, N = 405, Female = 93.6, N = 192 Diabetes: Male = 133.2, N = 208, Female = 117, N = 101 | - | Males:  Diabetes = 208  No Diabetes = 3407  Females:  Diabetes = 101  No Diabetes = 1334  Total:  Diabetes = 309  No Diabetes = 4741 | - | Verbal Memory Delayed Recall (Total) Male: NGT = 6.87 (1), IGT = 6.72 (1.03), Diabetes = 6.87 (1.2) Female: NGT = 6.96 (1), IGT = 6.75 (0.95), Diabetes = 6.24 (1.39)  Inductive Reasoning AH-4 Male: NGT = 48.7, IGT = 49.49, Diabetes = 47.26 Female: NGT = 41.79, IGT = 41.61, Diabetes = 39.73 |
| Launer et al., | **2015** | - | - | Total: Diabetes = 69  No Diabetes = 611 | - | Means and SD not available for extraction |
| Launer et al., | **1995** | - | - | - | - | Global Cognition CASI:  Low BP (Total): 83.9 (15.4) Normal BP (Total): 83.1 (15.6) Borderline BP (Total): 81.9 (16.3) Mixed BP (Total): 79.3 (17.2) High BP (Total): 74.9 (21.6) |
| Leong et al. | **2020** | - | - | Non hypertensive Wave 1 (Total): Diabetes = 64,  No Diabetes = 2216  Hypertensive without Medication Wave 1 (Total): Diabetes = 117,  No Diabetes = 2704  Hypertensive with Medication Wave 1 (Total):  Diabetes = 450,  No Diabetes = 2620 | - | - |
| Li et al. | **2024** | - | Normal cognitive function: 8.8 (7.5 – 10.7)  Cognitive impairment: 8.5 (7.4 – 10.5) | Total = 524  Normal cognitive function = 320  Cognitive impairment = 204 | - | MoCA  Normal cognitive function = 30 (29, 30)  Cognitive impairment = 29 (28, 29)  MMSE  Normal cognitive function = 27 (27, 28)  Cognitive impairment = 24 (22, 25) |
| Lin et al. | **2020** | - | - | Total: Diabetes = 37  No Diabetes = 491 | - | Attention Attention Index (MoCA - Total) = 16.28 (1.78)  Working Memory Memory Index Score (MoCA - Total): 12.72 (2.38)  Temporal Orientation Orientation Index Score (MoCA - Total): 5.84 (0.43)  Visuospatial Organisation Visual Index Score (MoCA - Total): 6.48 (0.92)  Global Cognition MoCA (Total): 26.1 (2.89) |
| Liu et al. | **2022** | Controls = 100.8 ± 12.6  SCI = 104.4 ± 14.4 | - | SCI, Controls  Diabetes = 11, 19  Non-Diabetes = 53, 73 | - | MoCA: Control = 27.4 (1.3), SCI: 23.3 (2.5) |
| Lopez-Oloriz et al. | **2014** | - | - | Total: Diabetes = 17  No Diabetes = 78 | - | - |
| Lutski et al. | **2019** | - | - | Apo-B ≥ 105:  Diabetes = 9  No Diabetes = 103  Apo-B < 105:  Diabetes = 25  No Diabetes = 200  Total:  Diabetes = 34  No Diabetes = 303 | - | - |
| Ma et al. | **2020** | - | - | Total = 27 | - | SWME  T2DM: 5.51 ± 3.63  Without T2DM: 0 ± 0  Visual Search  T2DM: 70.7% ± 0.2  Without T2DM: 88.3% ± 0.1  0-back  T2DM: 97.1% ± 0.0  Without T2DM: 98.1% ± 0.0  1-back  T2DM: 90.1% ± 0.1  Without T2DM: 93.7% ± 0.0 |
| Mahinrad et al. | **2020** | - | - | Baseline (Total): Diabetes = 29  No Diabetes = 162 | - | Verbal Memory Delayed Word Recall (Total): 10  Attention TMT-A (Total) = 28  Working Memory DSST (Total): 68 (16) Digit Span Backwards (Total): 18 (4) |
| Masi et al. | **2018** | - | Total = 5.8, N = 1157 | - | - | Attention Selective/Simple Reaction Time (Total): 281 (63.5) Choice Reaction Time (Total): 612 (76.5) |
| Masi et al. | **2015** | - | - | - | - | - |
| Mattei et al. | **2019** | Without T2DM (Total) = 97.0 ± 10.9  With T2DM (Total) = 154.6 ± 63.8  Controlled T2DM (Total) = 124.7 ± 46.5  Uncontrolled T2DM (Total) = 165.1 ± 65.8 | - | Without T2DM (Total) = 711  With T2DM (Total) = 465  Controlled T2DM (Total) = 118  Uncontrolled T2DM (Total) = 339 | - | Global Cognition  Overall  Without T2DM (Total) = 0.16 ± 0.54  With T2DM (Total) = −0.06 ± 0.51  Controlled T2DM (Total) = −0.004 ± 0.44  Uncontrolled T2DM (Total) = −0.08 ± 0.52  MMSE  Without T2DM (Total) = 23.6 ± 3.3  With T2DM (Total) = 22.8 ± 3.4  Controlled T2DM (Total) = 22.7 ± 3.2  Uncontrolled T2DM (Total) = 22.8 ± 3.4  Memory  Overall  Without T2DM (Total) = 0.31 ± 0.88  With T2DM (Total) = 0.03 ± 0.92  Controlled T2DM (Total) = −0.07 ± 0.92  Uncontrolled T2DM (Total) = 0.06 ± 0.91  DSB  Without T2DM (Total) = 3.4 ± 1.5  With T2DM (Total) = 3.0 ± 1.5  Controlled T2DM (Total) = 3.2 ± 1.5  Uncontrolled T2DM (Total) = 3.0 ± 1.4  Word List Learning  Without T2DM (Total) = 39.0 ± 11.6  With T2DM (Total) = 35.6 ± 10.6  Controlled T2DM (Total) = 35.5 ± 9.7  Uncontrolled T2DM (Total) = 35.7 ± 10.9  Executive Function  Overall  Without T2DM (Total) = 0.19 ± 1.0  With T2DM (Total) = −0.21 ± 0.94  Controlled T2DM (Total) = 0.04 ± 0.89  Uncontrolled T2DM (Total) = −0.29 ± 0.94  Word Recognition  Without T2DM (Total) = 30.9 ± 5.1  With T2DM (Total) = 30.0 ± 5.9  Controlled T2DM (Total) = 30.1 ± 5.6  Uncontrolled T2DM (Total) = 30.0 ± 6.1  Stroop  Without T2DM (Total) = 24.7 ± 11.2  With T2DM (Total) = 20.8 ± 9.3  Controlled T2DM (Total) = 20.8 ± 9.6  Uncontrolled T2DM (Total) = 20.9 ± 9.1  Clock Drawing  Without T2DM (Total) = 2.3 ± 1.0  With T2DM (Total) = 2.0 ± 1.1  Controlled T2DM (Total) = 2.1 ± 1.1  Uncontrolled T2DM (Total) = 1.9 ± 1.1  Figure Copying  Without T2DM (Total) = 10.6 ± 8.0  With T2DM (Total) = 8.0 ± 7.6  Controlled T2DM (Total) = 8.6 ± 7.7  Uncontrolled T2DM (Total) = 7.8 ± 7.6  Attention  DSF  Without T2DM (Total) = 7.2 ± 1.9  With T2DM (Total) = 7.0 ± 1.8  Controlled T2DM (Total) = 7.3 ± 1.9  Uncontrolled T2DM (Total) = 6.9 ± 1.8 |
| Mefford et al. | **2021** | - | - | 100 = 50  100–129 = 124  130–159 = 114  ≥160 = 123 | - | DSST  <100 = 70.6 (16.8)  100–129 = 71.1 (16.6)  130–159 = 70.4 (15.6)  ≥160 = 68.0 (15.1)  RAVLT  <100 = 9.0 (2.0)  100–129 = 9.1 (1.9)  130–159 = 9.0 (1.9)  ≥160 = 8.7 (1.9)  Stroop Test  <100 = 22.6 (11.8)  100–129 = 22.2 (10.5)  130–159 = 22.4 (9.8)  ≥160 = 24.0 (12.1) |
| Meyer et al. | **2022** | - | - | Total = 91 | - | MoCA (Total) = 24.4 ± 3.6  DSST (Total) = 69.2 ± 16.9  Stroop (Total) = 22.2 ± 11.5  RAVLT (Total) = 9.3 ± 1.9  Category fluency (Total) = 20.6 ± 5.2  Letter fluency (Total) = 42.6 ± 12.3 |
| Moore et al. | **2014** | - | - | - | - | Intelligence AFQT (Total): 61.8 (0.95) |
| Muhkerjee et al. | **2022** | - | Diabetic retinopathy: 7.1 ± 1.9  Without diabetic retinopathy: 6.8 ± 1.0 | Total = 72  (Diabetic retinopathy: 36; Without diabetic retinopathy: 36) | - | MMSE score  Diabetic retinopathy: 23.5 (3.3)  Without diabetic retinopathy: 25.3 (3.3)  Orientation domain score  Diabetic retinopathy: 8.2 (1.3)  Without diabetic retinopathy: 9.0 (1.1)  Registration domain score  Diabetic retinopathy: 2.8 (0.4)  Without diabetic retinopathy: 3.0 (0.2)  Attention/calculation domain score  Diabetic retinopathy: 3.7 (1.1)  Without diabetic retinopathy: 4.1 (1.3)  Recall domain score  Diabetic retinopathy: 2.1 (0.8)  Without diabetic retinopathy: 2.7 (0.5)  Language domain score  Diabetic retinopathy: 6.6 (1.2)  Without diabetic retinopathy: 6.6 (1.2) |
| Nunley et al. | **2017** | - | Total = 7.85 ± 1.85 | Total = 108 | - | Statin Use: Never used (n = 51); 1-6 yr (n = 25); 7-12 yr (n = 32)  Estimated verbal IQ 108.6 ± 8.2 107.7 ± 10.0 106.5 ± 6.9 0.24  Memory domain z-score 0.24 ± 0.75, -0.23 ± 0.64, -0.25 ± 0.78  Executive function z-score 0.18 ± 0.56, -0.10 ± 0.82, -0.30 ± 0.79  Psychomotor speed z-score 0.29 ± 0.66, -0.33 ± 1.10, -0.28 ± 0.89  Visuo- construction z-score 0.21 ± 0.64, -0.16 ± 0.82, -0.21 ± 1.45 |
| Olaya et al. | **2017** | - | - | - | - | Episodic memory: Summary Score: Total = 10.67 (3.15) |
| Olaya et al. | **2019** | - | - | Total: Diabetes = 476  No Diabetes = 6901  50-64 years old:  Diabetes = 205  No Diabetes = 4167 | - | Episodic memory: Summary Score: Total = 10.8 (3.1) |
| Otsuka et al. | **2019** | - | Men (n = 866)  < 5.6% (n = 549) = 5.3 ± 0.2  5.6 to < 6.0% (n = 214) = 5.8 ± 0.1  6.0 to < 6.5% (n = 67) = 6.2 ± 0.1  ≥ 6.5% (n = 36) = 7.3 ± 0.8  Women (n = 815)  < 5.6% (n = 571) = 5.3 ± 0.2  5.6 to < 6.0% (n = 175) = 5.8 ± 0.1  6.0 to < 6.5% (n = 44) = 6.2 ± 0.1  ≥ 6.5% (n = 25) = 7.3 ± 0.9 | - | - | Executive Function  Information Processing Speed  Men (n = 866)  < 5.6% (n = 549) = 54.5 ± 14.9  5.6 to < 6.0% (n = 214) = 50.1 ± 13.7  6.0 to < 6.5% (n = 67) = 49.6 ± 12.8  ≥ 6.5% (n = 36) = 47.2 ± 13.2  Women (n = 815)  < 5.6% (n = 571) = 56.7 ± 15.5  5.6 to < 6.0% (n = 175) = 51.7 ± 14.8  6.0 to < 6.5% (n = 44) = 43.6 ± 12.6  ≥ 6.5% (n = 25) = 48.2 ± 12.2 |
| Palacios-Mendoza et al. | **2018** | Diabetes (Total): 175.68 (84.95), N = 142 No Diabetes (Total): 94.67 (9.57), N = 167 | Diabetes (Total): 8.64 (2.55), N = 142 No Diabetes 5.61 (0.36, N = 167 | Total:  Diabetes = 142  No Diabetes = 167 | - | Verbal Memory RAVLT (Immediate Recall - Total): Diabetes = 6.57 (2.57), No Diabetes = 7.5 (2.68) RAVLT (Delayed Recall - Total): Diabetes = 6.35 (2.89), No Diabetes = 7.67 (2.69) RAVLT (Learning Score - Total): Diabetes = 34.38 (8.39), No Diabetes = 38.78 (8.3) ROCF (Immediate Recall - Total): Diabetes = 14.53 (7.42), No Diabetes = 17.76 (7.77) ROCF (Delayed Recall - Total): Diabetes = 13.28 (7.82), No Diabetes = 16.23 (7.82)  Attention TMT-A (Total): Diabetes = 72.92 (30.57), No Diabetes = 61.42 (30.04) Choice Reaction Time (Total): Diabetes = 1954.57 (658.59), No Diabetes = 1769.19 (580.65) Digit Span Forward Test (Total): Diabetes = 6.51 (1.72), No Diabetes = 7.1 (1.96)  Working Memory Digit Span Backwards (Total): Diabetes = 3.88 (1.73), No Diabetes = 4.41 (1.97) |
| Palta et al. | **2019** | - | - | Persistent Physical Activity ARIC Visits 1–3 (No PA - Total): Diabetes = 373, No Diabetes = 1623  Persistent Physical Activity ARIC Visits 1–3 (Low - Total): Diabetes = 86, No Diabetes = 658  Persistent Physical Activity ARIC Visits 1–3 (Middle - Total): Diabetes = 80, No Diabetes = 619  Persistent Physical Activity ARIC Visits 1–3 (High - Total): Diabetes = 101, No Diabetes = 1093 | - | - |
| Pan et al. | **2018** | - | - | Total:  Diabetes = 249, No Diabetes = 1788 45-54 years: Diabetes = 38, No Diabetes = 924 55-64 years: Diabetes = 66, No Diabetes = 797 | - | Verbal Memory Immediate Recall (Females): 3.91 (1.75)  Global Cognition Telephone interview of cognitive status (Derived from MMSE - Total): 6.82 (3.24) |
| Panigrahi et al. | **2021** | Total = 147.1 (23.2), N = 80 | Total = 7.2 (0.6), N = 80 | Total:  T2DM Patients: 80  No Diabetes = 0 | - | Global Cognition: MMSE score: 25.37 ± 3.34 |
| Passos et al. | **2021** | - | - | Never = 423  1999 and 2012 (Total) = 37  2012 (Total) = 10 | - | Learning  Age Group:  45-54 = 22  55-64 = 20  No Diabetes = 22  Diabetes 1999 and 2012 = 21  Diabetes 2012 = 19.5  Recall  Age Group:  45-54 = 7  55-64 = 7  No Diabetes = 7  Diabetes 1999 and 2012 = 7  Diabetes 2012 = 7.5  Semantic  Age Group:  45-54 = 21  55-64 = 18  No Diabetes = 20  Diabetes 1999 and 2012 = 19  Diabetes 2012 = 17  Phonemic Test  Age Group:  45-54 = 14  55-64 = 12  No Diabetes = 13  Diabetes 1999 and 2012 = 11  Diabetes 2012 = 10 |
| Patel et al. | **2019** | Total = 176.80 ± 8.58 | - | T1DM: 7  T2DM: 53 | - | ACE-lll  Attention  Baseline: 13.58±0.29  After one month: 14.03±0.26  After three months: 14.52±0.22  Memory  Baseline: 12.88±0.55  After one month: 13.48±0.40  After three months: 14.05±0.39  Verbal Fluency  Baseline: 4.38±0.28  After one month: 4.40±0.23  After three months: 4.48±0.23  Language  Baseline: 21.63±0.37  After one month: 22.28±0.25  After three months: 22.37±0.26  Visuospatial ability  Baseline: 11.65±0.34  After one month: 12.12±0.28  After three months: 12.65±0.26  Total score  Baseline: 64.13±1.50  After one month: 66.32±1.09  After three months: 68.07±0.96 |
| Pokharel et al., | **2019** | - | - | - | - | - |
| Power et al., | **2014** | - | - | Total:  Diabetes = 2076  No Diabetes = 11,858 | - | Verbal Memory Delayed Recall: Male = 6.3 (1.5), Female = 6.8 (1.5)  Working Memory DSST (Total): Hypertension = 42.4 (13.4), Normotensive = 46.4 (14.6) |
| Ravona-Springer et al. | **2020** | FH+ = 96.70 (16.14)  FH- = 95.93 (14.41) | FH+ = 5.76 (0.84)  FH- = 5.70 (0.47) | FH+ = 45  FH- = 9 | - | - |
| Rawlings et al., | **2018** | - | Total = 5.8 (1.2), N = 13,351  Diabetes = 8 (2.1), N = 1779  No Diabetes = 5.4 (0.4), N = 11,572 | Total:  Diabetes = 1799  No Diabetes = 11,572 | - | - |
| Razavi et al., | **2020** | Total: 105.5 (33), N = 960  White: Male = 106.4 (25.5), N = 270; Female = 103.4 (31.7), N = 365  Black: Male = 106.1 (29.3), N = 123; Female = 107.7 (44.4), N = 202 | - | Total:  Diabetes = 128  No Diabetes = 832 | - | - |
| Reis et al. | **2013** | Coronary artery calcified plaque  Present: 104.1 (31.2)  Absent: 96.7 (23.2)  Abdominal aortic calcified plaque  Present: 100.8 (27.4)  Absent: 96.5 (23.9) | - | Coronary artery calcified plaque  Present: 109  Absent: 186  Abdominal aortic calcified plaque  Present: 188  Absent: 107 | - | - |
| Richards et al. | **2005** | - | - | - | - | Verbal Memory Delayed Recall Males: None (0/day): 43 years = 23.45 (6.04), 53 years = 21.54 (6.44), Very light (0.1–1.0/day): 43 years = 24.6 (6.48), 53 years = 23.9 (6.42), Light (1.1-2.0/day): 43 years = 25.3 (5.41), 53 years = 24.28 (5.64), Moderate (2.1–4.0/day): 43 years = 25.47 (5.16), 53 years = 24.1 (6.39), Heavy (4.1–8.0/day): 43 years = 24.98 (5.98), 53 years = 23.99 (6.22) Females: None (0/day): 43 years = 25 (6.41), 53 years = 23.92 (6.02), Very light (0.1–1.0/day): 43 years = 26.45 (6.08), 53 years = 26.08 (5.93), Light (1.1-2.0/day): 43 years = 26.4(6.41), 53 years = 25.8 (5.99), Moderate/Heavy (2.1–8.0/day): 43 years = 28.02 (7.23), 53 years = 26.18 (5.79)  Global Cognition NART Males: None (0/day): 32.94 (9.71), Very light (0.1–1.0/day): 35.14 (9.97), Light (1.1-2.0/day): 35.98 (9.03), Moderate (2.1–4.0/day): 36.11 (9.19), Heavy (4.1–8.0/day): 34.88 (9.2) Females: None (0/day): 32.26 (9.27), Very light (0.1–1.0/day): 36.5 (8.54), Light (1.1-2.0/day): 34.67 (10.77) |
| Ritchie et al. | **2017** | - | - | Non-FH: Diabetes = 3  No Diabetes = 104  FH:  Diabetes = 7  No Diabetes = 96 | - | - |
| Root et al. | **2015** | - | - | - | - | Verbal Memory Delayed Recall: Males = 6.97 (1.42), Females = 6.42 (1.46)  Psychomotor Speed SDMT: Males = 48.2 (13.7), Females = 43.9 (12.5) |
| Rose et al. | **2010** | - | - | Total: OH No:  Diabetes = 1386  NO Diabetes = 10,664  OH Yes:  Diabetes = 121  No Diabetes = 531 | - | Verbal Memory Delayed Recall (Total): OH No = 6.6, OH Yes = 6.4  Working Memory: DSST (Total): OH No = 44.7, OH Yes = 42.6 |
| Sadahiro et al. | **2019** | - | - | Total = 117 | - | - |
| Salzwedel et al. | **2019** | - | - | Total: Diabetes = 92  NO Diabetes = 309 | - | Global Cognition MoCA (Total): Cardiac Rehabilitation admission = 25.3 (3); Cardiac Rehabilitation discharge = 25.7 (2.9) |
| Sierra et al. | **2004** | Without WML (Total): 97.2 (12.60, N = 37 With WML (Total): 95.4 (12.6), N = 23 | - | - | - | Attention Digit Span Forward Test (Total): Diabetes = 5.51 (0.97), No Diabetes = 4.86 (1.14)  Intelligence WAIS (Total): Without WML = 96 (21), With WML = 103 (26)  Working Memory Digit Span Backwards (Total): Without WML = 4.12 (0.99), With WML = 4 (0.43) |
| Singh-Manoux et al. | **2003** | - | - | - | - | Verbal Memory Delayed Recall Males: No Disease = 6.76, 6.18, 6.77, 6.76; Angina = 6.36, MI = 6.18, CHD = 6.3, IC = 6.02 Females: No Dieases = 7.27, 7.28, 7.26, 7.28, 7.24, Angina = 6.68, MI: = 6.87, CHD = 6.85, IC: = 7.65  Inductive Reasoning AH-4 Males: No Disease = 47.23, 47.3, 47.28, 47.33, 47.01; Angina = 44.31, MI = 45.36, CHD = 44.71, IC = 42.61 Females: No Dieases = 45.82, 45.79, 46.85, 46.52, 45.69, Angina = 42.42, MI: = 44.5, CHD = 43.82, IC: = 42.56 |
| Singh-Manoux et al. | **2005** | - | - | - | - | Verbal Memory Delayed Recall: Males = 6.88 (2.3), Females = 6.98 (2.64)  Inductive Reasoning AH-4: Male = 48.76 (9.94), Female = 41.68 (12.19) |
| Singh-Manoux et al. | **2009** | - | - | Total:  CHD:  Diabetes = 61  No Diabetes = 120  No CHD:  Diabetes = 777  No Diabetes = 4334 | - | Verbal Memory Delayed Recall (Total): 6.9 (2.4)  Inductive Reasoning AH-4 (Total): 47 (10.9) |
| Smith et al. | **2010** | - | - | - | - | - |
| Solomon et al. | **2009** | - | - | - | - | Episodic memory: Summary Score: Total = 5  Global Cognition MMSE (Total): 26 |
| Sun et al. | **2020** | - | - | Year 25 Examination (Total): Diabetes = 124  No Diabetes = 1245 | - | Verbal Memory RAVLT (Summary Metric - Total): 9.4 (3.1)  Working Memory DSST (Total): 75 (14.9) |
| Suvila et al. | **2021** | - | - | Total: Diabetes = 385  No Diabetes = 2561 | - | Verbal Memory RAVLT (Summary Metric - Total): 8.5 (3.4)  Working Memory DSST (Total): 68 (17)  Global Cognition MoCA (Total): 23.9 (3.9) |
| Szczesnia et al. | **2020** | - | - | Baseline, 3, and 6 year follow-up: Males: Diabetes = 161, No Diabetes - 30 Females: Diabetes = 306, No Diabetes = 446 Total: Diabetes = 76, No Diabetes = 467 | - | Attention TMT-A (Total): Healthy = 34.03 (10.38); Microbleeds = 35.14 (10.07); Low WMH = 37.27 (11.72); High WMH = 41.80 (13.71); Infarcts = 44.06 (13.98)  Working Memory DSST (Total): Healthy = 67.67 (15.37); Microbleeds = 69.36 (15.4); Low WMH = 63.76 (13.67); High WMH = 55.93 (12.37); Infarcts = 57.94 (16.63)  Global Cognition MoCA (Total): Healthy = 26.86 (2.5); Microbleeds = 26.5 (1.7); Low WMH = 26.14 (2.5); High WMH = 25.2 (2.62); Infarcts = 25.12 (3.07) |
| Szoeke et al. | **2016** | Baseline (Total): 95.22 (21.96), N = 387 | - | - | - | Verbal Memory CERAD (Delayed - Total): 7.67 (1.53) CERAD (Immediate - Total): 7.24 (1.1) CVLT (Immediate - Total): 8.77 (2.06) CVLT (Delayed - Total): 8.81 (3.23) |
| Tipnis et al. | **2022** | - | - | Diabetes = 150  No Diabetes = 150 | - | HMMSE  Diabetes = 26.81 (2.66)  No Diabetes = 27.51 (2.43) |
| Tufvesson et al. | **2013** | - | - | Baseline (Total): Diabetes = 69  No Diabetes = 864 | - | Global Cognition MMSE (Total): 28.1 (1.8) |
| Tuligenga et al. | **2014** | - | - | Normoglycaemia (n=4703); Prediabetes (n=648); Newly diagnosed diabetes (n=115); Known diabetes (n=187) | - | - |
| Udayakuma et al. | **2018** | - | - | Total = 100 | - | - |
| Veugen et al. | **2018** | Total = 108 (28.8), N = 3009  <60 yrs = 104.4 (30.6), N = 1380 | - | Total: Diabetes = 407  No Diabetes = 1135 | - | Global Cognition MMSE (Total): 29 (1.2) |
| Walker et al. | **2019** | - | - | 1st Quartile (Total): Diabetes = 130, No Diabetes = 2882 2nd Quartile (Total): Diabetes = 192, No Diabetes = 2809 3rd Quartile (Total): Diabetes = 312, No Diabetes = 2683 4th Quartile (Total): Diabetes = 597, No Diabetes = 2396 | - | - |
| Wang et al. | **2016** | Total = 102.42 )29.16), N = 3048 | - | Total: Diabetes = 415  No Diabetes = 2633 | - | - |
| Wang et al., | **2018** | - | - | Total:  Diabetes = 1994  No Diabetes = 11,726 | - | - |
| Wei et al., | **2018** | - | - | Normotensives:  Diabetes = 203  No Diabetes = 4521  Controlled Hypertensives:  Diabetes = 84  No Diabetes = 611  Untreated Hypertensives:  Diabetes = 31  No Diabetes = 312  Treated but Uncontrolled Hypertensives:  Diabetes = 108  No Diabetes = 862 | - | - |
| Wharton et al., | **2014** | Total: 89.17 (9.77), N = 571 | - | - | - | - |
| Whitaker et al. | **2021** | - | - | Male:  Diabetes = 71  No Diabetes = 751  Female:  Diabetes = 83  No Diabetes = 1065  Total:  Diabetes = 154  No Diabetes = 1816 | - | Working Memory DSST: 72.98±15.20   Verbal Memory RAVLT: 8.83±3.18  Executive Function: Stroop: 21.40±9.82 |
| Wieczorek et al. | **2016** | - | - | Total: Diabetes = 15  No Diabetes = 59 | - | Global Cognition MMSE (Total): 29 |
| Winkler et al. | **2014** | - | - | Male (Middle age): Diabetes = 64  No Diabetes = 451  Female (Middle age): Diabetes = 35  No Diabetes = 539  Total (Middle age):  Diabetes = 99 No Diabetes = 990 | - | Verbal Memory Immediate Recall: Male = 5.7 (1.1), Female = 5.82 (1.2) Delayed Recall: Male = 4.08 (1.6), Female = 4.2 (1.7)  Visuospatial Organisation Clock Drawing Test: Males = 28 (5), Females = 55 (10) |
| Wod et al. | **2018** | - | - | - | - | - |
| Wolf et al. | **2007** | - | - | - | - | - |
| Yaffe et al. | **2014** | Baseline (Total): 82 (10.9), N = 3381 | - | - | - | Verbal Memory RAVLT (Delayed Recall - Total): 8.3 (3.3)  Working Memory DSST (Total): 69.9 (16.2) |
| Wu et al. | **2022** | - | - | Diabetes-Free = 7036  Treated Diabetes = 250  Untreated Diabetes = 628 | - | - |
| Yang et al. | **2018** | HIV Uninfected (Total): 91, N = 900 | HIV Uninfected (Total) = 5.2, N = 900 | HIV Uninfected (Total): Diabetes = 63  No Diabetes = 837 | - | Verbal Memory RAVLT (Delayed Recall - Total): 9.8 (3.4) RCF (Delated Recall - Total): 21.1 (7.6)  Attention TMT-A (Total): 25.8 (10.3)  Psychomotor Speed SDMT (Total): HIV Uninfected = 53.7 (12.8) |
| Yano et al. | **2018** | - | - | Visit 1 (Total): Diabetes = 788  No Diabetes = 10,621 | - | Verbal Memory Delayed Recall (Total): 5.2 (1.9)  Working Memory DSST (Total): 43.7 (13.4) |
| Yano et al. | **2014** | Baseline (Y0 - Total): 82.1 (11.4), N = 2326 | - | - | - | - |
| Ylilauri et al. | **2017** | Cholesterol intake quartile, mg/d: 1 (<331) Total: 84.6 (18) Cholesterol intake quartile, mg/d: 2 (331–387) Total: 86.4 (25.2) Cholesterol intake quartile, mg/d: 3 (388–458) Total: 86.4 (21.6) Cholesterol intake quartile, mg/d: 4 (>458) Total: 86.4 (21.6) Egg intake quartile, g/d: 1 (<14) Total: 88.2 (23.4) Egg intake quartile, g/d: 2 (14–25) Total: 86.4 (25.2) Egg intake quartile, g/d: 3 (26–43) Total: 84.6 (14.4) Egg intake quartile, g/d: 4 (>43) Total: 86.4 (21.6) | - | Cholesterol intake quartile, mg/d: 1 (<331) Total: Diabetes = 5% Cholesterol intake quartile, mg/d: 2 (331–387) Total: Diabetes = 6% Cholesterol intake quartile, mg/d: 3 (388–458) Total: Diabetes = 6% Cholesterol intake quartile, mg/d: 4 (>458) Total: Diabetes = 7% Egg intake quartile, g/d: 1 (<14) Total: Diabetes = 8% Egg intake quartile, g/d: 2 (14–25) Total: Diabetes = 4% Egg intake quartile, g/d: 3 (26–43) Total: Diabetes = 5% Egg intake quartile, g/d: 4 (>43) Total: Diabetes = 6% | - | Verbal Memory Selective Reminding Test (Total): Cholesterol intake quartile, mg/d: <339 (284) = 34.5 Cholesterol intake quartile, mg/d: 339–406 (370) = 34.4 Cholesterol intake quartile, mg/d: >406 (456) = 34 Egg intake quartile, g/d: <16 (8) = 34 Egg intake quartile, g/d: 16–32 (23) =34  Egg intake quartile, g/d: >32 (45) = 34.9  Working Memory Visual Reproduction Test (Total):  Cholesterol intake quartile, mg/d: <339 (284) = 11.3 Cholesterol intake quartile, mg/d: 339–406 (370) = 11.3 Cholesterol intake quartile, mg/d: >406 (456) = 11.3 Egg intake quartile, g/d: <16 (8) = 11.2 Egg intake quartile, g/d: 16–32 (23) =11.1 Egg intake quartile, g/d: >32 (45) = 11.6  Global Cognition MMSE (Total): Cholesterol intake quartile, mg/d: <339 (284) = 27.1 Cholesterol intake quartile, mg/d: 339–406 (370) = 27 Cholesterol intake quartile, mg/d: >406 (456) = 27.1 Egg intake quartile, g/d: <16 (8) = 27 Egg intake quartile, g/d: 16–32 (23) = 27.1 Egg intake quartile, g/d: >32 (45) = 27.2 |
| Young et al. | **2006** | - | - | Baseline (Total): No Diabetes = 7148 | - | Verbal Memory Delayed Recall (Total) With Hyperinsulinemia Fasting insulin (cutoff 75th percentile) = 6.58 (1.42) Fasting insulin (cutoff 12.2 mU/l) = 6.59 (1.4) HOMA (cutoff 75th percentile) = 6.56 (1.41) HOMA (cutoff 2.6) = 6.64 (1.41)  Without Hyperinsulinemia Fasting insulin (cutoff 75th percentile) = 6.83 (1.42) Fasting insulin (cutoff 12.2 mU/l) = 6.85 (1.43) HOMA (cutoff 75th percentile) = 6.82 (1.43) HOMA (cutoff 2.6) = 6.86 (1.43)  Working Memory DSST (Total):  With Hyperinsulinemia Fasting insulin (cutoff 75th percentile) = 43 (13.7) Fasting insulin (cutoff 12.2 mU/l) = 43.7 (13.7) HOMA (cutoff 75th percentile) = 42.8 (13.8) HOMA (cutoff 2.6) = 44.4 (13.7)  Without Hyperinsulinemia Fasting insulin (cutoff 75th percentile) = 47.9 (13.1) Fasting insulin (cutoff 12.2 mU/l) = 48.1 (13.1) HOMA (cutoff 75th percentile) = 47.8 (13.2) HOMA (cutoff 2.6) = 48.4 (13) |
| Yulug et al. | **2020** | - | - | T2DM = 15  Prediabetes = 16  New-onset diabetes = 15 | - | - |
| ZekiAlHazzouri et al. | **2015** | - | - | Total: Diabetes = 355  No Diabetes = 2263 | - | Working Memory DSST (Total): 70.9 (15.8) |
| Zhang et al. | **2019** | No Diabetes (Total) = 100.3 (14.1), N = 7151 Controlled Diabetes (Total) = 130.8 (41.1), N = 460 Untreated Diabetes (Total) = 139.5 (41.1), N = 413 Treated but Uncontrolled Diabetes (Total) = 151.6 (69.5), N = 511 | No Diabetes (Total) = 5.1 (0.4), N = 7151 Controlled Diabetes (Total) = 5.8 (1.2), N = 460 Untreated Diabetes (Total) = 6 (1.4), N = 413 Treated but Uncontrolled Diabetes (Total) = 6.8 (1.8), N = 511 | No Diabetes (Total) = 7151 Controlled Diabetes (Total) = 460 Untreated Diabetes (Total) = 413 Treated but Uncontrolled Diabetes (Total) = 511 | - | Episodic Memory Score: No Diabetes (Total): 3.2 (1.9) Controlled Diabetes (Total): 3.1 (1.7) Untreated Diabetes (Total): 2.9 (1.9) Treated but Uncontrolled Diabetes (Total): 3.2 (1.9) |

**Supplemental Table B.7.** List of Cognitive tests used across all cognitive domains.

| Cognitive Variable | Cognitive Test(s) |
| --- | --- |
| Memory  Verbal Memory | Immediate and Delayed Verbal Memory, Spot the Word test, East Boston Memory test, Rey Auditory Verbal Learning Test (Immediate & Delayed recall, Learning & Summary Score), Selective Reminding Test, Rey–Osterreith complex figure (Immediate & Delayed), Consortium to Establish a Registry for Alzheimer’s Disease (Immediate & Delayed), California Verbal Learning Test (Immediate & Delayed), Word List Learning |
| Working Memory | Digit Symbol Substitution Test, Composite Score, Chinese Clinical Memory Scale, Digit Span Backwards, McNair Survey, WAIS-IV Digit Sequencing, Memory Index Score (MoCA), Visual Reproduction Test |
| Attention | Trail making Test Part A, Choice Reaction Time, Simple Reaction Time, Digit Span Forward Test, 5-Choice Movement Test |
| Intelligence | Wechsler Adult Intelligence Scale, IQ, Mental Rotation Test |
| Executive Function  Letter Cancellation | Letter Search Speed Test, Letter Cancellation Composite Score |
| Verbal Fluency | Word Fluency test, Boston Naming Test, Mill Hill Vocabulary Test, Phonemic Fluency Test, Semantic Fluency Test, Verbal Index Score, Benson Delay Test, Buschke Delay Test |
| Processing Speed | Trail-making Test Part-B, Trail-making Test Part B minus A, Stroop Test (Interference Time), Word Matching Test, Composite Executive Score, Rapid Visual Processing (Cambridge Neuropsychological Test Automated Battery & Isolated), Stroop Colour Word Test, Executive Index Score (MoCA), Visual Search Speed, Labyrinth Test |
| Global Cognition | Mini-Mental State Exam, Montreal Cognitive Assessment, Informant Questionnaire on Cognitive Decline in the Elderly, Cambridge Cognition Examination, National Adult Reading Test, Multilingual Naming Test, Intelligenz-Struktur-Test, Børge Priens Prøve, Addenbrooke’s cognitive examination, U.S. Health and Retirement Study Composite Score, Consortium to Establish a Registry for Alzheimer’s Disease |
| Inductive Reasoning | Alice Heim 4-I |
| Psychomotor Speed | Symbol Digits Modalities Test |
| Visuospatial Organisation | Block Design Test, Visual Index Score (MoCA), Clock Drawing Test |
